# Determining the gas-phase structures of α-helical peptides from shape, microsolvation, and intramolecular distance data

**DOI:** 10.1038/s41467-023-38463-z

**Published:** 2023-05-22

**Authors:** Ri Wu, Jonas B. Metternich, Anna S. Kamenik, Prince Tiwari, Julian A. Harrison, Dennis Kessen, Hasan Akay, Lukas R. Benzenberg, T.-W. Dominic Chan, Sereina Riniker, Renato Zenobi

**Affiliations:** 1 Laboratorium für Organische Chemie, D-CHAB, ETH Zürich, 8093 Zurich, Switzerland; 2 Laboratorium für Physikalische Chemie, D-CHAB, ETH Zürich, 8093 Zurich, Switzerland; 3 Department of Chemistry, The Chinese University of Hong Kong, Hong Kong SAR, People’s Republic of China; 4 Present Address: Laboratory of Atmospheric Chemistry, Paul Scherrer Institute, Forschungsstrasse 111, 5232 Villigen PSI, Switzerland; 5 Present Address: University of Münster, MEET Battery Research Center, Corrensstrasse 46, 48149 Münster, Germany

**Keywords:** Mass spectrometry, Bioanalytical chemistry

## Abstract

Mass spectrometry is a powerful technique for the structural and functional characterization of biomolecules. However, it remains challenging to accurately gauge the gas-phase structure of biomolecular ions and assess to what extent native-like structures are maintained. Here we propose a synergistic approach which utilizes Förster resonance energy transfer and two types of ion mobility spectrometry (i.e., traveling wave and differential) to provide multiple constraints (i.e., shape and intramolecular distance) for structure-refinement of gas-phase ions. We add microsolvation calculations to assess the interaction sites and energies between the biomolecular ions and gaseous additives. This combined strategy is employed to distinguish conformers and understand the gas-phase structures of two isomeric α-helical peptides that might differ in helicity. Our work allows more stringent structural characterization of biologically relevant molecules (e.g., peptide drugs) and large biomolecular ions than using only a single structural methodology in the gas phase.

## Abstract (web summary)

It remains challenging to gauge the gas-phase structure of biomolecular ions and assess to what extent native-like structures are maintained. Here, the authors utilize Förster resonance energy transfer and ion mobility spectrometry for more stringent structural characterization of biomolecular ions.

## Introduction

Soft ionization techniques^1, 2^ for mass spectrometry (MS) are thought to retain the structural characteristics of biomolecules when transferred from solution into the gas phase^3,4^. This ‘native MS’ approach is used to determine the stoichiometry of noncovalent complexes, binding constants, melting temperatures^5–7^, thermodynamics^8^, and folding kinetics^9^. The ability of native MS to allow structural studies in a controlled chemical environment (i.e., a solvent-free, high purity, and selected charge state) is intriguing. Other MS−based methods that rely on some form of labeling followed by fragmentation^10,11^, like hydrogen−deuterium exchange^12^, chemical cross-linking^13^, and covalent labeling methods^14^, can be combined with computational studies for structural analysis of biomolecules and their complexes. However, it is still not known to what extent the original solution-phase protein structure is retained in the gas phase. To answer this open question, structural probes, such as circular dichroism, ion mobility spectrometry or fluorescence spectroscopy, more specifically Förster resonance energy transfer (FRET), have been coupled to MS for conformational studies of biomolecular ions in the gas phase^15–19^. Such studies are complicated, and normally only a single structural methodology is applied.

Among the methodologies used, ion mobility-mass spectrometry (IM-MS), including traveling wave and drift tube types, is most common to investigate the overall shape of gas-phase ions. The collisional cross section (CCS) of an ion is proportional to its size, i.e., the rotationally-averaged surface area available for collisions with a neutral gas (e.g., He, N_2_). From IM-MS data, CCS values can be calculated directly or obtained after calibration. The CCS is then usually compared with theoretical values obtained from possible model conformations. However, multiple conformers are often indistinguishable by IM-MS, necessitating the utilization of additional spectroscopic data to properly elucidate conformational heterogeneity^20–23^. Differential ion mobility spectrometry (DMS) operates under atmospheric pressure and separates ions based on their differential mobility (Δ*K*) between low- and high-electrical fields^24^. As opposed to traveling wave and drift tube ion mobility separation, DMS separation is related to many physicochemical properties of ions, including microsolvation, the binding energy between ion and gas modifier (e.g., acetonitrile and isopropanol), and the rate of ion-modifier formation/dissociation^25–30^.

FRET, which is based on the distance dependence of a radiation-free transfer of energy from a donor to an acceptor chromophore^31^, provides an orthogonal structural information compared to IM-MS. Intramolecular distances between donor and acceptor chromophores can be derived from FRET data (fluorescence lifetimes and emission spectra). FRET is commonly used to investigate the structure of biomolecules in solution; in the gas phase, it is challenging due to low ion density^18^. Gas-phase FRET measurements are now becoming feasible in ion trap mass spectrometers, yet the acquired structural information is normally insufficient for obtaining comprehensive geometrical information of biomolecular ions^32–34^. Two research groups, the Dugourd group and ours, have started to obtain complementary information, shape and intramolecular distances, by combining IM-MS and FRET experiments, yet this approach is still in its infancy and needs further development^35–38^.

**Fig. 1:**
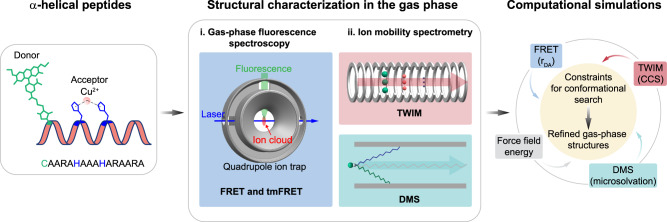
Structural characterization of an α-helical peptide in the gas phase. Donor-acceptor distances (r_DA_) of α-helical peptide ions are obtained from FRET or transition metal ion FRET (tmFRET) measurements in a modified quadrupole ion trap mass spectrometer. Collisional cross sections (CCS) of ions are determined in a traveling wave ion mobility (TWIM) spectrometer. Microsolvation data of ions are derived from differential ion mobility spectrometry (DMS) separation. Together with force field energies, this information is then combined to obtain refined gas-phase structures.

Here, we use information from a combination of DMS, traveling wave ion mobility (TWIM), and FRET measurements to guide simulations for determining gas-phase structures of biomolecular ions with a level of confidence that was not possible (Fig. 1). We show (i) that DMS provides complementary structural information to TWIM and FRET, and (ii) that microsolvation information from DMS separation can significantly improve the reliability of biomolecular structure prediction in the gas phase.

## Results

### Structural evaluation of an α-helical peptide using FRET, IM-MS, and DMS

To test the approach, we first investigated an alanine rich peptide, which is expected to form a stable α-helix in aqueous solution^39^. The α-helical peptide was labeled with carboxyrhodamine 6g (cR6G-tmP1, Fig. 1 and Supplementary Fig. 1) and then tested using transition metal ion FRET (tmFRET) in the gas phase. This is a recent extension of FRET, which enables the detection of short (e.g., 10 − 40 Å) distances in biomolecules^40^. In tmFRET, Cu^2+^ serves as an acceptor chromophore that binds noncovalently to a His-X_3_-His motif (X refers to any amino acid), which avoids the need for a second dye labeling step. By monitoring the reduction in fluorescence lifetime when the donor fluorophore is close to the acceptor Cu^2+^ ion, the FRET efficiency can be measured and then converted to donor-acceptor distance (r_DA_).

The results (Supplementary Figs. 2a and 3) showed lifetimes of 6.80 ns and 6.05 ns for the [M + 3H]^3+^ and [M+Cu+3H]^5+^ ions, respectively. Two lifetimes of 1.46 ns and 6.40 ns were deduced for the fluorescence decay curve of the [M+Cu+H]^3+^ ions from a double-exponential fit, which allows one to deduce the presence of two coexisting conformers. The two lifetime values correspond to two r_DA_ values, 20.9 Å and 41.2 Å for the [M+Cu+H]^3+^ ions (i.e., the existence of two major conformers), which is further supported by the observation of two peaks (565 Å^2^ and 630 Å^2^) in the TWIM results (Supplementary Fig. 2b). For [M+Cu+3H]^5+^ ions, one r_DA_ value of 36.8 Å and one major peak (830 Å^2^) in TWIM indicates an expanded conformation of the 5+ charge state ions due to significant Coulombic repulsion. Multiple conformers are frequently found in biomolecules, which significantly adds to the difficulty of structural studies based on FRET and IM-MS. TWIM measurements on other rhodamine 110-labeled α-helical peptides (rh110-tmP2, -tmP3, and -tmP4) also showed the existence of multiple conformations (Supplementary Fig. 4a–b, and Supplementary Table 2).

Since DMS separation is orthogonal to TWIM separation, an on-line coupling of DMS to TWIM and FRET could potentially disentangle the complexity of multiple gas-phase structures by separating conformers. The corresponding DMS separations of cR6G-tmP1, rh110-tmP2, -tmP3, and -tmP4 ions showed differences in the number of detected peaks and their distributions compared to TWIM data (Supplementary Figs. 2c, 4c−d, and 6−14). These results highlight the necessity of multiple methodologies to understand biomolecular structures in the gas phase.

### DMS coupled to fluorescence spectroscopy and IM-MS for structural characterization of isomers and conformers

In FRET studies of biomolecules, several dye labeling-site isomers may be obtained based on the labeling approach chosen. The presence of such isomers can severely complicate structural interpretation. To demonstrate the on-line coupling of our home-built DMS device (Supplementary Fig. 15) to the other two techniques for conformer separation, we used dye labeling-site isomers of a polyalanine-based α-helical peptide, similar to tmP1, with the sequence Ac-*C*AAAHAAAHAAAAHAAAHAAA*C*AK-NH_2_ (P1). The peptide was covalently labeled with Atto 532 dye in one of the two available cysteine residues using a maleimide linker (Supplementary Fig. 1). The two resulting labeling-site isomers (P1-Atto 532/Atto 532-P1) cannot be fully separated by HPLC (Supplementary Fig. 16), therefore all experiments were performed using the mixture.

**Fig. 2:**
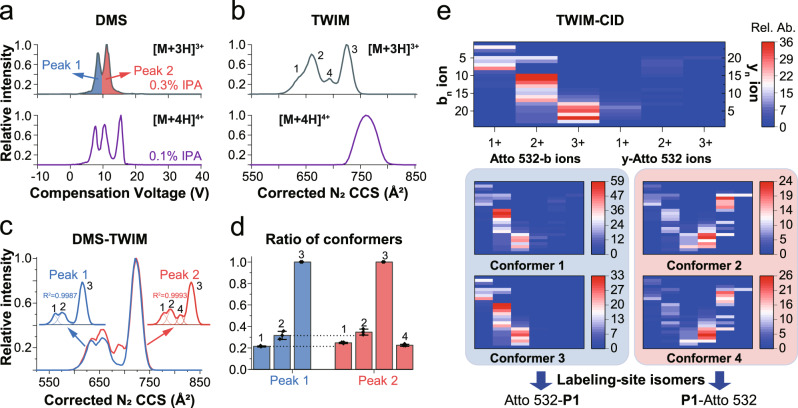
Conformational characterization of a mixture of dye-labeled peptide isomers. **a** Differential ion mobility spectrometry (DMS) ionograms (i.e., spectra), and **b** traveling wave ion mobility (TWIM) collisional cross section (CCS) distributions of the [M + 3H]^3+^ and [M + 4H]^4+^ ions of **P1**-Atto 532/Atto 532-**P1** mixture. In DMS, isopropanol (IPA), a common gaseous additive (i.e., gas modifier), was added into the carrier gas to enhance the separation of peaks 1 (blue) and 2 (red). Average CCS values of each conformer determined experimentally are given in Supplementary Table 3. **c** TWIM CCS distributions of the [M + 3H]^3+^ ion in the DMS-TWIM measurements. The compensation voltage (CV) was set to 8 V and 10.25 V for peaks 1 and 2, respectively. **d** Peak ratio of conformers differentiated by DMS-TWIM measurements extracted from Fig. 2c. The error bars denote mean ± SEM for *n* = 3 independent experiments. **e** TWIM-collision-induced dissociation (CID) experiments for all conformers together (top) and each conformer (bottom). Diagnostic b/y fragment ions with the dye attached to the N- or C-terminal cysteine are presented in the heat map. Relative abundances are normalized to the intensity of the [M + 3H]^3+^ precursor ion. Source data are provided as a Source Data file.

The DMS separation yielded two peaks (called peak 1 and peak 2) for the [M + 3H]^3+^ ion and three peaks for the [M + 4H]^4+^ ion at isopropanol (IPA) concentrations of 0.3 mol % and 0.1 mol %, respectively (Fig. 2a and Supplementary Figs. 17–18). Gas-phase fluorescence spectroscopic measurements were used to examine the structural difference between ions associated with peaks 1 and 2. The resulting emission spectra showed identical maxima and shapes (Supplementary Fig. 19), which suggests a similar solvation environment or polarization of the dye in the electric field from other charges^33,41^.

MS-based fragmentation methods can identify labeling-site isomers, since the dye is linked to either the N- or C-terminal cysteine in the mixture. However, collision-induced dissociation (CID) and photodissociation of ions associated with peaks 1 and 2 exhibit fragment ions from both labeling-site isomers (Supplementary Figs. 21–22), suggesting the presence of both labeling-site isomers in peaks 1 and 2, respectively.

The CCS distributions of both the [M + 3H]^3+^ and [M + 4H]^4+^ ions of the P1-Atto 532/Atto 532-P1 mixture were measured to obtain a better understanding of the gas-phase conformation (Fig. 2b). The TWIM CCS distribution of the [M + 3H]^3+^ ions contained four peaks (referred to as conformers 1 to 4), which suggest these ions adopt multiple conformations in the gas phase. The [M + 4H]^4+^ ions, on the other hand, exhibited only a single, broad CCS distribution.

It is worth noting that the TWIM or DMS data alone could not fully distinguish the labeling-site isomers or conformers, therefore, the DMS device was coupled on-line to TWIM for further analysis of peaks 1 and 2 of the [M + 3H]^3+^ ion (Fig. 2c). Using a compensation voltage (CV) of ~8 V (i.e., peak 1), three conformers were detected by TWIM, while four conformers were detected when the CV was set to ~10.25 V (i.e., peak 2). Conformer 4, an ion population with a CCS value of ~ 690 Å^2^, was only observed in the TWIM CCS distribution of peak 2. The relative intensities of conformers 1 and 2 are slightly lower in peak 1 than peak 2 (Fig. 2d).

CID experiments were conducted after DMS–TWIM separation (i.e., DMS–TWIM–CID) for the [M + 3H]^3+^ ions (top) and each conformer (bottom). Several coexisting conformers were differentiated and assigned to the respective labeling-site isomer by this approach (Supplementary Fig. 24 and Supplementary Tables 4–7). In CID experiments, the two labeling-site isomers are expected to show diagnostic b- or y-type fragment ions plus the dye attached to the N- or C-terminal cysteines (Supplementary Tables 4–8). A higher relative abundance of Atto 532-b ions (i.e., b type ions with the dye on the N-terminal cysteine) was found for the [M + 3H]^3+^ ions (top), which suggests that there is a higher proportion of the Atto 532-P1 labeling-site isomer in the mixture (Fig. 2e and Supplementary Fig. 24). For each conformer, a high relative abundance of Atto 532-b ions was also observed in conformers 1 and 3, while a high relative abundance of y-Atto 532 ions (i.e., y ions with the dye on the C-terminal cysteine) was observed in conformers 2 and 4. The CID results suggest that conformers 1 and 3 correspond to the Atto 532-P1 isomer, while conformers 2 and 4 originate from the P1-Atto 532 isomer. The improved selectivity of the DMS–TWIM–CID approach demonstrates the power and utility of such an orthogonal approach for conformational studies.

### Structural elucidation of two isomeric α-helical peptides with IM-MS and FRET

IM-MS and FRET can provide complementary shape and intramolecular distance constraints for molecular modeling of gas-phase ions. Previous investigations on polyalanine-based peptides showed that a C-terminal lysine stabilizes an α-helix in the gas phase by favorably interacting with the macrodipole of the helix^42–45^. Inspired by this, we applied this approach to determine the gas-phase structures of two isomeric α-helical peptides (P1 and P2). P1 has a C-terminal lysine, while P2 (Ac-*KA*CAAAHAAAHAAAAHAAAHAAAC-NH_2_) has a N-terminal lysine (Supplementary Fig. 1). These two peptides were doubly labeled on the two available cysteine residues with cR6G and QSY7 as donor fluorophore and acceptor chromophore, respectively. cR6G was selected because it has a much higher quantum yield compared to rh110 and Atto 532. Solution-phase FRET experiments of cR6G-P1-QSY7 and cR6G-P2-QSY7 (Supplementary Fig, 26 and Supplementary Table 9) suggest that cR6G-P1-QSY7, which has a C-terminal lysine, maintains much of its helical structure in solution.

**Fig. 3:**
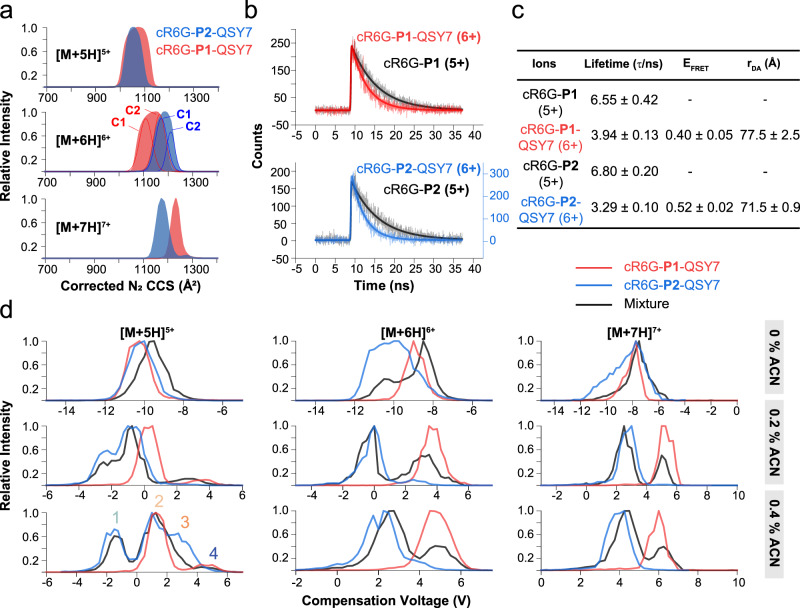
Structural characterization of two isomeric α-helical peptides in the gas phase. **a** Normalized traveling wave ion mobility (TWIM) collisional cross section (CCS) distributions of cR6G-**P1**-QSY7 and cR6G-**P2**-QSY7 ions in a 7.5 V wave height. **b** Fluorescence decay curves of the [M + 5H]^5+^ ions of cR6G-**P1** and cR6G-**P2**, and the [M + 6H]^6+^ ions of cR6G-**P1**-QSY7 and cR6G-**P2**-QSY7. **c** Table of lifetime values (τ), FRET efficiencies (E_FRET_), and donor-acceptor distance (r_DA_) values. Lifetime values are obtained from the *n* = 3 independent fluorescence decay curves fitted with GaussMod. **d** DMS ionograms of cR6G-**P1**-QSY7 (red), cR6G-**P2**-QSY7 (blue) ions, and their mixture (black) in 0, 0.2, and 0.4 mol % acetonitrile (ACN) as the gas modifier. Source data are provided as a Source Data file.

The mass spectra acquired using native conditions showed that the dominant peak of both peptides is the [M + 6H]^6+^ ion (Supplementary Fig. 28), which was then selected for conformation analysis. TWIM separations showed partial separation of the conformational populations for 5 + , 6 + , and 7+ charge states (Fig. 3a). Two distinct conformers are observed for each peptide in the 6+ charge state; we refer to these as C1 and C2. The average experimental CCS values (Supplementary Table 10) of the [M + 6H]^6+^ ion of cR6G-P1-QSY7 are smaller than those of cR6G-P2-QSY7, which suggests that the former ion is more compact, as expected.

Gas-phase FRET was then conducted to obtain complementary intramolecular distance information (Fig. 3b). Fluorescence lifetime values of the [M + 6H]^6+^ ions of cR6G-P1-QSY7 and cR6G-P2-QSY7 were significantly shortened compared with those of the [M + 5H]^5+^ ions of cR6G-P1 and cR6G-P2 that only contain the donor fluorophore. The fluorescence decay curves were fitted with GaussMod (Fig. 3c), single-exponential (Supplementary Table 11), and double-exponential fits (Supplementary Table 12), which indicate only one conformation for all ions. Because QSY7 is considered to be singly charged, the above-mentioned two charge states (5+ and 6 + ) thus must have the same number of charges on the peptide backbone and were used to calculate the FRET efficiencies (E_FRET_) in Fig. 3c. The Förster distance in the gas phase (R_*0, gas*_) was estimated to be 72.2 Å (refer to Methods). Therefore, the experimental donor-acceptor distance (r_DA_) values of the [M + 6H]^6+^ ion of cR6G-P1-QSY7 and cR6G-P2-QSY7 are 77.5 ± 2.5 Å, and 71.5 ± 0.9 Å, respectively (Fig. 3c). These FRET distance estimates thus contradict the IM-MS results in terms of the compactness of the two isomers, revealing the drawbacks of relying on only one single structural probe and the utility of hyphenating two techniques.

### Using geometrical constraints from IM-MS and FRET for computational studies to determine the gas-phase structures

**Fig. 4:**
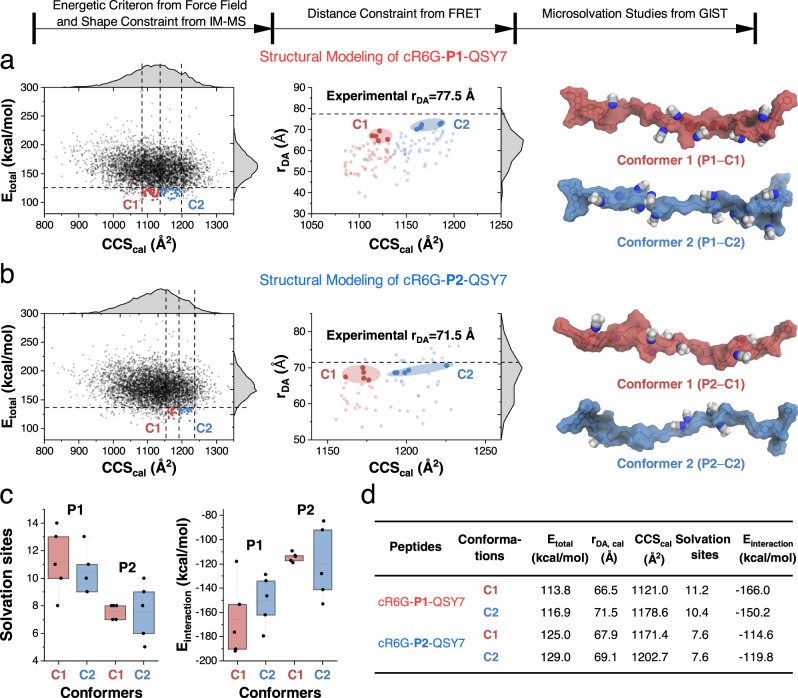
Computational simulations aid structural analysis of two isomeric α-helical peptides in the gas phase. **a** and **b** Force field total energies (E_total_) and calculated collisional cross section (CCS_cal_) values (defined as CCS_TJM* value from IMPACT) of 5000 candidate structures for the [M + 6H]^6+^ ion of cR6G-**P1**-QSY7 and cR6G-**P2**-QSY7. 20% energy cutoff, and experimental CCS values of conformer 1 (**C1**, red) and 2 (**C2**, blue) were applied to refine these structures. Further selection was achieved via experimental donor-acceptor distance (r_DA_) values for each IM-MS conformer. The representative structure (i.e., the lowest energy structure) from the refined twenty structures are presented with the solvation sites filled with ACN molecules. **c** The number of solvation sites and interaction energies (E_interaction_)of the [M + 6H]^6+^ ions are presented as box plots, where five structures per each cR6G-**P1**-QSY7 conformers have more solvation sites than cR6G-**P2**-QSY7 ions and consequently also more favorable interaction energies. The number of ACN molecules that cluster with the peptide ion depends on the available solvation sites. The error bars denote mean ± SEM for *n* = 5 independent experiments. Box plots: center at median, box bounds 25th and 75th percentiles, whiskers minima and maxima. **d** The calculated refinement parameters and microsolvation calculation results for the 5 refined structures per conformer (Supplementary Table 14). Source data are provided as a Source Data file.

To rationalize the seemingly conflicting results from FRET and IM-MS measurements, we integrated the collected geometrical data (i.e., shape and distance) as constraints in a molecular modeling cascade. The central technique in our structural modeling workflow is based on MD simulations. All simulations were carried out with the AmberTools21 simulation package^46^ and the GROMACS 2020.5 simulation engine^47–49^ using the Amber force fields 14SB^50^. An extensive conformational search was conducted via a simulated annealing protocol, which led to 5000 structural models for each peptide. These structures were then refined based on their force field energies, experimental CCS values, and r_DA_ values (Fig. 4a–b). In the model structure, the calculated r_DA_ value is defined by the distance between two carbon atoms at the para-position (C9) in the xanthene of donor and acceptor fluorophores. For each peptide we selected ten structures (five structures per IM-MS conformer).

For cR6G-P1-QSY7, all selected structures exhibit calculated r_DA_ values lower than the experimental ones (Fig. 4a). Therefore, the five structures which had the values closest to the experimental ones were selected for each conformer (Supplementary Fig. 33 and Supplementary Table 14). Note that experimental r_DA_ values (77.5 Å) are higher than the average values of the selected five structures from these two IM-MS conformers (66.5 Å for C1, 71.7 Å for C2). This indicates that the distances obtained from gas-phase FRET are overestimated by 14.1% and 7.5% for the C1 and C2 conformers, respectively. The overestimation is acceptable, and is probably largely explained by an overestimation of R_*0, gas*_ resulting from limited knowledge of gas-phase properties (e.g., free rotation of the dye)^33^.

Some candidate structures of the cR6G-P2-QSY7 ions (Fig. 4b) have higher calculated r_DA_ values than the experimental value (71.5 Å). Two conditions were then applied for the selection: (i) we required the FRET results to indicate a qualitatively shorter r_DA_ value for the [M + 6H]^6+^ ions of cR6G-P2-QSY7 than of cR6G-P1-QSY7; and (ii) a smaller r_DA_ values of candidate structures compared to the experimental r_DA_ values, which could be explained by a slight overestimation of R_*0, gas*_ in the gas phase as discussed above. Therefore, five structures with a calculated r_DA_ value close to, but below the experimental r_DA_ value were selected for each conformer (Supplementary Fig. 33 and Supplementary Table 14). The average r_DA_ values of two conformers were 67.9 Å and 69.1 Å, as discussed above for cR6G-P1-QSY7 ions, this again represents an acceptable overestimation of 12.3% and 10.8% for conformers C1 and C2, respectively.

Within these twenty selected structural models, the average force field energy of C1 structures was generally lower than that of C2 structures for both peptides (Supplementary Fig. 33 and Supplementary Table 14). The lowest energy structures from each conformer of the two peptides are depicted as representative structures in Fig. 4a–b (see pdb structures in Supplementary Tables 15–18). Both peptides were predicted to have elongated structures, yet each contained unique features: the cR6G moiety is oriented away from the peptide chain in both peptides, while QSY7 either folded onto (cR6G-P1-QSY7) or away from the peptide backbone (cR6G-P2-QSY7). The results show that cR6G-P1-QSY7 structures exhibit an α-helix (P1–C1) or minor turns (P1–C2), while cR6G-P2-QSY7 structures show large turns or random coils. These features are common among the twenty refined structures (Supplementary Fig. 33), which is in line with the initial assumption that the C-terminal lysine in P1 stabilizes the α-helix in the gas phase. The structural differences lead to a reduction in CCS values, but higher r_DA_ values for cR6G-P1-QSY7 ions. These models hence already provide reasonable structural suggestions to interpret the seemingly contradictory findings from the IM-MS and FRET experiments. In turn, this suggests that the combination of two techniques can provide complementary geometrical information for molecular modeling, which enhances the understanding of peptide conformational ensembles in the gas phase. An improved accuracy of CCS measurements in IM-MS, a more accurate estimation of R_*0, gas*_, and utilization of trajectory methods for calculating the CCS values could further facilitate the refinement of candidate structures.

### Assessing refined structures with microsolvation information from DMS measurements

To test the reliability of the refined twenty structural models, we employed gas modifier-assisted DMS separations for the cR6G-P1-QSY7 and cR6G-P2-QSY7 ions. The two peptides could not be separated when using isopropanol (IPA) as the gas modifier (Supplementary Fig. 34). Therefore, acetonitrile (ACN) was employed (Supplementary Figs. 35–37). The resulting DMS ionograms showed good separation of the [M + 5H]^5+^, [M + 6H]^6+^, and [M + 7H]^7+^ ions in the presence of 0.2 or 0.4 mol % ACN (Fig. 3d). The [M + 5H]^5+^ ions showed four peaks when using 0.4 mol % ACN as the gas modifier, of which peak 1 and 3 are likely originating only from cR6G-P2-QSY7. The [M + 6H]^6+^ and [M + 7H]^7+^ ions of the two peptides were nearly baseline-resolved. The results revealed more significant ion-molecule clustering (i.e., cluster formation) for the [M + 6H]^6+^ ions of cR6G-P1-QSY7 compared with cR6G-P2-QSY7.

The interaction potential (or binding energy) of the dynamic cluster formation and the microsolvation states of ions could be probed by DMS separation^28,51,52^. To computationally model this microsolvation process in the gas phase, we performed an analysis of the interaction potential between the twenty refined structures and ACN molecules in the gas phase. Subtle differences in the structure will affect the number of available solvation sites and interaction energies with ACN molecules^30^. In a first step, we identified the most favorable interaction sites based on grid inhomogeneous solvation theory (GIST) calculations^53,54^. Based on the GIST predictions, we modeled the microsolvated complexes and calculated the solvation interaction energies. It was observed that ACN molecules interact strongly with the peptide backbone (Fig. 4a–b). The results (Fig. 4c–d and Supplementary Table 14) indicated ≈11 solvation sites on average for the [M + 6H]^6+^ ion of cR6G-P1-QSY7. By comparison, ≈8 solvation sites on average were found for the cR6G-P2-QSY7 structures.

As we modeled the peptide structures in complex with explicit ACN molecules, it was also possible to estimate the interaction energies. Low interaction energies between ions and ACN molecules suggest a higher magnitude of cluster formation, which would induce a higher differential mobility (Δ*K*) in a DMS separation. Conformers C1 and C2 of cR6G-P1-QSY7 showed an average interaction energy of −166.0 kcal/mol and −150.2 kcal/mol, respectively. For cR6G-P2-QSY7, the average interaction energies of −114.6 kcal/mol and −119.8 kcal/mol for conformers C1 and C2. In addition, we followed an alternative modeling approach where we always included the positively charged cR6G and QSY7 as favorable interaction sites (Supplementary Table 19). Both strategies to model microsolvation revealed a more favorable cluster formation for the [M + 6H]^6+^ ion of cR6G-P1-QSY7 compared to cR6G-P2-QSY7. Consequently, this leads to a larger CV shift for the [M + 6H]^6+^ ion of cR6G-P1-QSY7 than cR6G-P2-QSY7 (Fig. 3d and Supplementary Figs. 35–37). Hence, the agreement between the microsolvation calculations and the DMS experimental results provides further support for the structural refinements when IM-MS and FRET data are used as structural constraints.

## Discussion

In this work, hyphenation of a DMS device to IM-MS and FRET allows in-depth conformational analysis of isomers. We demonstrated the utility of integrating data obtained by multiple gas-phase techniques as constraints for molecular modeling to better determine the gas-phase structure of α-helical peptides. A conformational search was conducted to refine the gas-phase structures with force field energies, intramolecular distances obtained from FRET measurements, and shape constraints obtained from IM-MS measurements. These constraints improve the reliability of the structural models significantly and allow the assessment of α-helix stability in the gas phase. The result of structural-refinements also allowed us to interpret the opposite indications from the IM-MS and FRET experiments.

Moreover, microsolvation calculations suggested a statistically significant difference of solvation sites and interaction energy for the 20 refined structures. The [M + 6H]^6+^ ions of cR6G-P1-QSY7 exhibit more solvation sites and lower interaction energies on average, which is consistent with a more favorable ion-modifier noncovalent cluster formation derived from the DMS separation. The strategy of separating conformers by DMS prior to FRET or IM-MS is particularly promising. Even without the gas-phase FRET, DMS data and the microsolvation modeling could serve as a supplement to IM-MS in the growing field of structural biology. Both ion mobility techniques are commercially available but rarely interfaced. Combining three complementary experimental techniques, DMS, TWIM, and FRET, with computational studies allows the determination of reliable conformational models, as exemplified here for α-helical peptides. This approach could be applied to answer how ions behave in the gas phase, which is of fundamental importance to the field of native MS. Utilization of this approach to characterize the gas-phase structure of biologically relevant molecules (e.g., peptide drugs) and larger biomolecules than α-helical peptides under native conditions could be valuable.

## Methods

### Materials

Atto 532 with a maleimide linker was purchased from Atto-Tec (Siegen, Germany). Carboxyrhodamine 6g (cR6G) with a different maleimide linker length was purchased from Setareh Biotech (Oregon, USA). tmP1 (CAARAHAAAHARAARA), P1 (CAAAHAAAHAAAAHAAAHAAACAK), and P2 (KACAAAHAAAHAAAAHAAAHAAAC), with N-terminal acetylation and C-terminal amidation, were purchased from GenScript Biotech (Leiden, Netherland). tmP2 (C_2_H_3_H_7_, ACHAAKHAKAAAAAKA), tmP3 (C_2_H_6_H_10_, ACAAKHAAKHAAAAKA), and tmP4 (C_2_H_10_H_14_, ACAAKAAAKHAAAHKA) are rhodamine 110 dye-labeled peptides, which were commercially obtained (Thermo Fisher Scientific, USA) in lyophilized form, with N-terminal acetylation and C-terminal amidation. These alanine rich peptides were selected because they are thought to exhibit stable α-helix formation both in the solution and gas phase^39,55^. Rhodamine 110 (rh110) was labeled to cysteine (Cys2) using a C6-maleimide linker. The labeling processes were also performed for cR6G-tmP1, P1-Atto 532, cR6G-P1-QSY7, and cR6G-P2-QSY7, according to an established protocol^56^. Briefly, before labeling cysteine groups with maleimide dyes, a reduction of the cysteine residue was performed using dithiothreitol (DTT). The excess DTT was removed by HPLC purification and the reduced peptide was lyophilized. The peptide powder was dissolved in 100 mM potassium phosphate buffer (pH = 7.1 for maleimide) at a concentration of ~2 mg/mL, and reactive dye with a maleimide linker was dissolved in DMF at a concentration of 10 mg/mL. Reactive dye solution (20 μL) was added to the sample solution, slowly vortexed, and incubated for 1 h with ice cooling. Finally, the sample was purified using an HPLC equipped with a UV/Vis detector (Supplementary Notes 4.1 and 5.1). Fully/partially separated components were collected and then lyophilized. Aliquots of the samples were analyzed by mass spectrometry (i.e., exact mass measurement and collision-induced dissociation, CID) to verify the identity of the labeling products. The dye-labeled biomolecules were dissolved in water and analyzed by UV/Vis spectroscopy at 522 nm (cR6G), 532 nm (Atto 532), and 560 nm (QSY7) to determine the quantity. The molar extinction coefficient (ε) in H_2_O for each dye are 94000, 115000, and 90000, respectively. Then samples were lyophilized again for storage. Before analysis, the samples were dissolved in water, and adjusted to a concentration of ~10−20 μM. The structure of samples used in this work are shown in Scheme S1.

### DMS instrumentation and experiments

In DMS, the ions are transported by a carrier gas orthogonal to a varying alternating current electric field, which is usually called as dispersion field. The peak-to-peak voltage of dispersion field is called dispersion voltage (DV). Depending on the differential mobility (Δ*K*) of the ion, a net-movement of ion beam towards one of the electrodes results. By utilizating a direct current voltage, called as compensation voltage (CV), to one of two parallel electrodes, ions with a specific Δ*K* are stabilized and transmitted from the DMS device. During the separation, other ions would collide with one of the electrodes and consequently discharge. DMS separation can be tuned by the electric field strength (dispersion voltage, DV), residence time, carrier gas composition, and gas modifier (type and concentration)^26,28,30,57^. Gas modifiers in DMS separations are designed to mix with the carrier gas and influence the ion transport via cluster formation. The gas modifier concentration is crucial, likely resulting from the rate of clustering/ declustering and the ion-modifier binding energy^26–30^. The interaction between the ion and gas modifier molecule(s) are qualitatively displayed as their shift of CV. DMS separation is related with many physicochemical properties of ions, including microsolvation, CCS, and pK_a_^25,58^. Direct calculation of CCS values from DMS data is possible for low m/z metabolites using machine learning^59,60^. DMS is also a highly mobile device that can be coupled to IM-MS for arrival time distribution measurements, which enable the CCS measurements^61,62^.

In this study, a home-built nanoelectrospray ionization (nanoESI or nESI) DMS device (see Supplementary Note 2.3 and Supplementary Fig. 15) modified from a previous design was used^63^. Briefly, a nanoESI source was mounted inside an aerodynamic optimized polyether ether ketone (PEEK) housing (housing 1), whose design was inspired by the CaptiveSpray source (Bruker). The nanoESI source was connected to a counter electrode with a 2.0 mm i.d. skimmer. The counter electrode was grounded during the experiments. After passing through the skimmer, the ion cloud was focused through a second circular channel (~3 mm in axial direction, housing 2) and then entered the parallel DMS electrodes. Nebulizer gas (~1.1 L/min) was introduced into housing 1 to assist the nanoESI spray. Auxiliary gas (~0.1 L/min) was introduced into housing 2 to focus the ion cloud and increase the ion transmission. The nebulizer gas and auxiliary gas mix together and are served as the carrier gas between DMS electrodes. The DMS setup comprises two hemispherical electrodes made of stainless steel and fixed towards a cylindrical PEEK housing (housing 3). The resulting dimensions of the DMS cell were 30 × 10 × 1.0 mm (length × width × gap height). The DMS device was fixed onto a 3D translation stage, and then aligned with different mass spectrometer inlet before the routine experiment. The interface between the DMS device and the mass spectrometer inlet was left open (with a distance of ~2 mm). Therefore, nebulizer gas and auxiliary gas (i.e., carrier gas), instead of the vacuum drag or throttle gas by an external pump, define the ion residence time. The ions were carried by the carrier gas to pass through the DMS device, which is different from other DMS setups (like SelexIon from AB Sciex and FAIMS Pro from Thermo Scientific). In this work, the ion residence time is around 15 ms on average.

The spray voltage was maintained at ~1.0 kV in general. The dispersion voltage (DV) was kept to 4.5 kV for all experiments. The ions could be scanned in a CV range of −100 V to 100 V (i.e., broadband scan mode) with a pre-defined step in the power supply (HV100-12, Stahl-electronics). A narrow CV range (narrowband scan mode) was usually applied to reduce the data acquisition time. LabVIEW scripts were programmed to synchronize the mass spectra acquisition and CV scan. In the gas modifier-assisted DMS experiments, a stepwise modulation of gas modifier concentration from 0 to 0.5 mol % was applied according to an established protocol^26,27^. Gas modifier, like isopropanol (IPA) or acetonitrile (ACN) solvent, was injected into a home-built porous *polytetrafluoroethylene* (PTFE) gas mixer. The gas mixer consists of a housing packed with multiple porous PTFE plates with a thickness of 2 mm each (HiPep Laboratories). The selection of the gas modifier and its concentration are still largely empirical for optimal separation of different analytes. It is worth to mention that a slight shift of the peak position in the resulting DMS ionogram is usually due to the fluctuation of modifier concentration and the asymmetric waveform (amplitude and shape). In this work, DMS was coupled with a modified quadrupole ion trap (QIT) MS for the gas-phase fluorescence spectroscopy experiments (i.e., emission spectroscopy and fluorescence lifetime). Moreover, DMS was also connected to a Synapt G2-S QTOF-MS for the tandem DMS–TWIM experiments.

### Ion mobility mass spectrometry (IM-MS)

In this work, IM-MS experiments (Supplementary Note 2.2) were performed in a Synapt G2-S QTOF-MS (Waters). For the nanoESI experiment, 5 μL of 10-20 μM peptide sample was loaded into a ~ 1.5 μm i.d. pulled glass capillary (1.0 mm o.d., 0.75 mm i.d.). The glass capillary was pulled in house by a micropipette puller (P-1000, Sutter Instrument). The spray voltage (~1 kV) was applied via a platinum wire. For IM-MS measurements, corrected N_2_ CCS values of ions were calculated by using poly-DL-alanine in the Major Mix IMS/Tof calibration kit as the calibrant, and calibration was fitted by linear fit method. 7.0 V, 7.5 V, and 8.0 V wave height voltages were applied to determine the variation of calibration. Some improved calibration approach could further reduce the uncertainty, such as a reported method with over 2500 experimental TWIM data sets^64^. Raw files were acquired by MassLynx (version 4.1, Waters) and converted into mzxml format by MSConvert (Version: 3.0.21193-ccb3e0136, ProteoWizard), further processed by Matlab scripts (R2021a, mathworks) to generate DMS ionogram. All data were then analyzed using OriginPro 2021 (OriginLab).

IM-MS experiments were also performed in a SELECT SERIES Cyclic IMS (cIM, Waters) for a better separation of P1-Atto 532 and Atto 532-P1 mixture (Supplementary Fig. 23). Three peaks could easily be identified in the resulting ion mobility spectra with one or two passes in the cIM. Conformer 4 was missing, which is likely due to a low concentration of P1-Atto 532 in the mixture sample used for cIM separation (refer to the discussion section of Fig. 2 in the manuscript). Besides, differences could also result from the instrumental conditions (in terms of softness). Nevertheless, conformational and the CCS distributions for the [M + 3H]^3+^ ion of P1-Atto 532 in two IM-MS instruments were similar.

### Fluorescence spectroscopic measurements

The instrumentation and methodology of mass-selected fluorescence measurements in the gas phase are described elsewhere^32^. Briefly, a quadrupole ion trap (QIT) MS was modified and coupled with tunable laser excitation and highly sensitive fluorescence detection systems. Gaseous ions, generated using nanoESI, are trapped in the QIT that allows optical access for laser irradiation. For the tmFRET experiments, 50-100 μM CuCl_2_ in water was added into the peptide samples to form the copper adducted ions. Ions were accumulated for 1 s, and then trapped in the ion trap (*q*_z_ = 0.75). Mass isolation of 1 s was then conducted. Meanwhile, the trapped ions were irradiated for 1 s by a laser beam from a tunable Ti:Sapphire fs laser (MaiTai, Spectra-Physics, U.S.A.) at a wavelength of 460 nm and a laser power of ~10 mW. The helium buffer gas was injected into the trap to cool the ion packets and reduce the photodissociation (PD) due to laser irradiation. Mass spectra of isolated ions were acquired during the fluorescence measurements. Mass spectrometric data were recorded by LCQ Tune Plus (version 2.0, Thermo Fisher). The emitted fluorescence was then collected from a 5.0 mm diameter hole drilled into the ring electrode of the QIT and is directed toward the detection setup. Around 1.8% of the emitted fluorescence reaches the detectors. The collected fluorescence was collimated and sent to the spectrograph with an electrothermally-cooled CCD. The time-resolved fluorescence measurement was performed with a Single-Photon Avalanche Diode (SPAD) using Time Correlated Single Photon Counting (TCSPC). Fluorescence spectra were recorded by Solis (version 4.31.30024.0, Andor), fluorescence decay curves were recorded by Time Harp 260 software (version 3.0.0.0, PicoQuant). All data were then analyzed using OriginPro 2021 (OriginLab).

Solution-phase FRET experiments of cR6G-P1-QSY7 and cR6G-P2-QSY7 (Supplementary Note 5.2, Supplementary Figs. 26–27, and Supplementary Table 9) were conducted in the home-built in-plume ESI setup^62^. As QSY7 is a dark quencher and could potentially undergo photobleaching, the experiments were conducted with freshly prepared samples in a low ~μW laser power and limited lifetime acquisition time.

### Distance estimation from FRET efficiency (E_FRET_)

The methodology to estimate the FRET efficiency (E_*FRET*_), Förster distance in the gas phase (R_*0, gas*_), and distances of donor-acceptor distance (r_DA_) were based on an established model from literature^40,65^. The donor (cR6G) emission and acceptor (QSY7) absorption spectra were acquired to calculate the spectral overlap (*J*) in the solution phase (Supplementary Fig. 33). Further, the quantum yield of the donor dye (cR6G) in the solution (*Φ* = 0.63) was incorporated into the calculation^66^. The refractive index of water and vacuum were 1.33 and 1.0, respectively. In this work, *J* is =3.848*10^15^ nm^4^/(M*cm), the solution phase R_0_ (R_*0, solution*_) for cR6G and QSY7 was 59.7 Å, and the gas phase R_0_ (R_*0, gas*_) was 72.2 Å. For tmFRET experiments, the excitation spectrum of cR6G and absorption spectrum of CuCl_2_ solution in pH ∼7 (Supplementary Fig. 32) was used to calculate *J*, which gave a value of 8.444*10^12^ nm^4^/(M*cm) in ae software (FluorTools). Therefore, R_*0, gas*_ for cR6G and Cu^2+^ pair was determined to be 26.0 Å.

Lifetimes presented in this work were determined by fitting of the experimental data using a gaussian-modified exponential function (GaussMod). Here, we explored further possibilities, such as a single-exponential fit and multi-exponential decay using the DecayFit software (version 1.4, FluorTools, www.fluortools.com). The instrument response function (IRF) was obtained from the gaussian-modified exponential fit of a high signal-to-noise data set.

### Force field parameters

The AmberTools21 simulation package^46^ was used to prepare the peptide starting structures for subsequent simulations. For the peptides’ standard amino acid residues we assigned parameters, i.e., force field charges and atom types, according to the Amber force field 14SB^50^. To derive the force field parameters for the modified amino acids that were labeled with a chromophore we used the antechamber module. Antechamber is included in the AmberTools21 software package and allows an automatic force field parametrization of organic molecules. In this procedure we applied the AM1-BCC model^67,68^ to calculate atomic charges and derived atom types from the general amber force field 2^69^.

### Conformational search

To efficiently sample a diverse conformational ensemble for each peptide, we employed an extensive simulated annealing protocol. In this protocol the temperature was elevated to 1500 K within 5000 simulation steps, from step 5001 to 400000 the peptide structure freely moved at 1500 K. The system was then cooled down again to 0 K from step 400001 to step 450000, where it was simulated for another 50000 simulation steps. The resulting conformation was then reused as starting structure for the another simulated annealing run. We first repeated this heating and cooling cycles to generate 100 conformations. From each of those structures we started 50 more simulated annealing cycles, thus totaling to 5000 conformations per peptide. For these 5000 final conformations we performed an additional energy minimization with a maximum of 5000 cycles, where the minimization algorithm switched from steepest descent to conjugate gradient after 1000 steps. The temperature in our simulations were regulated using the Langevin thermostat^70^ implemented in the sander simulation engine. All bonds involving hydrogens were constrained using the SHAKE algorithm^71^.

For subsequent microsolvation calculations, we refined the diverse conformational ensembles generated for each peptide based on the experimental CCS values and FRET distances as well as their force field energies (20% cutoff). The CCS values for each conformation were estimated using the ion mobility projection approximation calculation tool, IMPACT (version: 0.9.1, University of Oxford)^72^. Distances between the dyes were calculated using cpptraj^73^. The refined 20 structures and their exact ranges for CCS values, FRET distances, and energies are shown in Supplementary Fig. 33 and Supplementary Table 14. The trajectory method was also applied to calculate the CCS values (named as CCS_TJM) of the refined 20 structures using Collidoscope with default settings^74^.

### Microsolvation calculations

To estimate the interaction potential of each peptide with acetonitrile, we derived favorable interactions sites using grid inhomogeneous solvation theory (GIST)^53,54^. GIST was originally developed to calculate the interaction energies of biomolecular systems in explicit water simulations^75,76^ and has recently been generalized for all rigid solvents^49,77,78^. The fundamental concept was to simulate the restrained peptide conformation in an explicit acetonitrile solvent box and subsequently evaluated all energetic contributions on a grid. Acetonitrile (ACN) concentration used during the DMS experiments was higher enough to allow sufficient molecules for clustering also in the simulation. For these restrained simulations we generated solvated coordinates and topologies for the selected conformations with a minimum wall distance of 3 nm. Simulations were then performed using the GROMACS 2020.5 simulation engine^47–49^, using ParmEd to convert all required files. The acetonitrile solvent box was first minimized with the steepest descent algorithm for a maximum of 50000 minimization steps. To equilibrate the solvated system, we first heated up the system for 1 ps to 300 K in the NVT ensemble. Here we applied the LINCS^79^ algorithm to constrain all bonds involving hydrogens to allow for a time step of 2 fs. We chose the particle mesh Ewald approach for the treatment of long-range electrostatic interactions and a cutoff distance of 1 nm for short range electrostatics and van der Waals interactions. For pressure equilibration 1 ns of simulation time was acquired using the Berendsen barostat^80^ with a time constant of 2 ps to maintain atmospheric pressure. All following production runs were performed with the same conditions in the NPT ensemble using a force constant of 1000 kcal/mol per atom. For each conformation we collected a simulation time 50 ns.

The analysis of interaction energies using GIST was performed with the GPU-accelerated version of cpptraj^81^. A simulation of a pure acetonitrile solvent box was used to estimate to solvent-solvent interaction energy of acetonitrile (−7.006 kcal/mol). With the gisttools analysis package we then generated density maps of the interaction energy for each conformation. We then identified the most probable microsolvation sites based on the areas with the lowest interaction energies considering only voxels with a interaction energy below −1.5 kcal/mol. Each of the identified sites was then used to guide the placement of explicit acetonitrile molecules in the modeling of the microsolvated complexes. We estimated the interaction energy of each conformation with acetonitrile as modifier molecule using the linear interaction energy scheme implemented in cpptraj^73^.

### Reporting summary

Further information on research design is available in the Nature Portfolio Reporting Summary linked to this article.

## Supplementary information

Supplementary Information

Reporting Summary

## Data Availability

Source data are provided with this paper. PDB structures shown in the manuscript are attached to the supplementary Tables 15–18. The original data used in this publication are made available in a curated data archive at ETH Zurich (https://www.researchcollection.ethz.ch) under the DOI: 10.3929/ethz-b-000545943.
