# Supplementary material for: Determining the gas-phase structures of α-helical peptides from shape, microsolvation, and intramolecular distance data
Source: Nat Commun. 2023 May 22;14:2913. doi: 10.1038/s41467-023-38463-z (PMC10203302; doi:10.1038/s41467-023-38463-z)
Supplement: Supplementary file 1 — Supplementary Information [file 41467_2023_38463_MOESM1_ESM.pdf]

Supplementary Information

Determining the Gas-Phase Structures of α-Helical Peptides from Shape, Microsolvation, and Intramolecular Distance Data

Ri Wu,[1‡] Jonas B. Metternich,[1‡] Anna S. Kamenik,[2‡] Prince Tiwari,[1,4] Julian A. Harrison,[1] Dennis Kessen,[1,5] Hasan Akay,[1] Lukas R. Benzenberg,[1] T.-W. Dominic Chan,[3] Sereina Riniker,[2*] and Renato Zenobi[1*]

[1]Laboratorium für Organische Chemie, ETH Zürich, D-CHAB, 8093 Zürich, Switzerland.
[2]Laboratorium für Physikalische Chemie, ETH Zürich, D-CHAB, 8093 Zürich, Switzerland.
[3]Department of Chemistry, The Chinese University of Hong Kong, Hong Kong SAR, People's Republic of China.

Present addresses:
[4]Laboratory of Atmospheric Chemistry, Paul Scherrer Institute, Forschungsstrasse 111, 5232 Villigen PSI, Switzerland.
[5]University of Münster, MEET Battery Research Center, Corrensstrasse 46, 48149 Münster, Germany.

[‡]These authors contributed equally to this work

*Corresponding author's email:
Sereina Riniker: sriniker@ethz.ch
Renato Zenobi: zenobi@org.chem.ethz.ch

Contents

Supplementary Note 1. Sample preparation 2
Supplementary Note 2. DMS, IMS and tmFRET methods 2
Supplementary Note 3. DMS instrumentation and experiments 16
Supplementary Note 4. DMS, IMS and fluorescence spectroscopic measurements of singly labelled α-helical peptide 17
Supplementary Note 5. DMS, IMS and fluorescence spectroscopic measurements of doubly labelled isomeric α-helical peptides 38
Supplementary Note 6. Microsolvation studies 76
Supplementary Note 7. References 77

Supplementary Note 1. Sample preparation

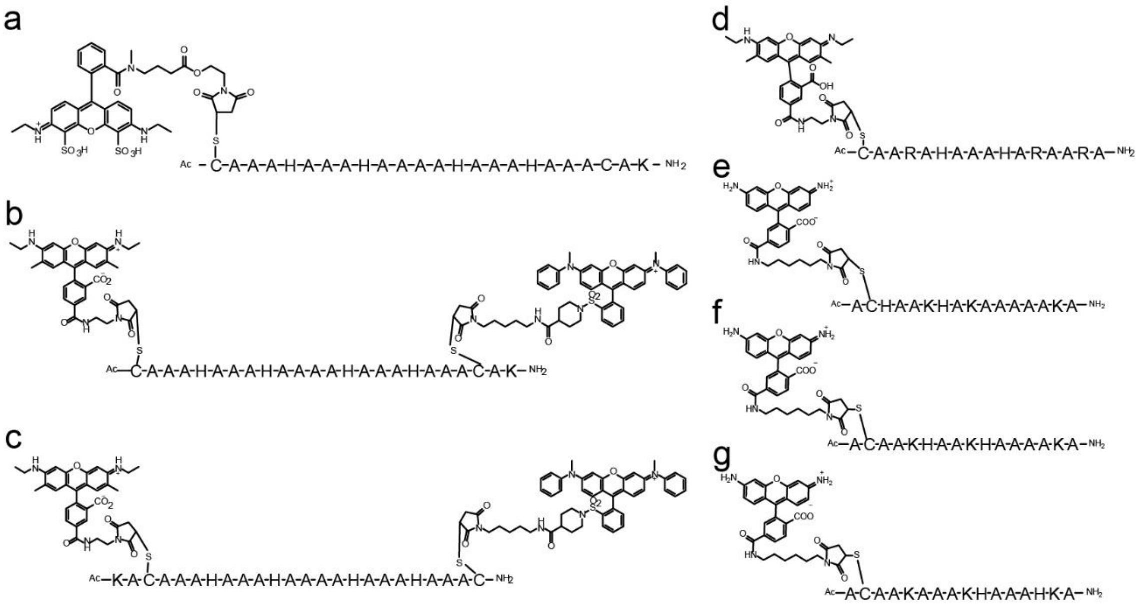

Supplementary Figure 1. Structure of, (**a**) **P1**-Atto 532, (**b**) cR6G-**P1**-QSY7, (**c**) cR6G-**P2**-QSY7, (**d**) cR6G-tm**P1**, and (**e-g**) rh110-tm**P2**, rh110-tm**P3**, and rh110-tm**P4**. The distance between the two cysteines in **P1** and **P2** was optimized to fit for the measurements of large biomolecular backbone distances by traditional FRET.

Supplementary Note 2. DMS, IMS and tmFRET methods

Transition metal ion FRET (tmFRET) is a recent extension of FRET experiment in the gas phase, which enable the measurement of shorter biomolecular backbone distances (10−40 Å) compared to traditional FRET.[1] In this work, four polyalanine-based peptides (tm**P1-4**) were labeled with rhodamine110 (rh110) or carboxyrhodamine 6g (cR6G) as donor fluorophore. The acceptor dye was replaced with a transition metal ion (i.e., Cu^{2+}), which bind noncovalently to a His-X_3-His motif. With a simplified lifetime measurement of donor dye signal reduction, tmFRET preserves high distance sensitivity and could serve as a molecular ruler in the gas phase. tmFRET also facilitates small Förster distance (R_0) values compared to traditional FRET. Therefore, tmFRET is more sensitive to conformational changes of peptides and proteins. Fluorescence decay curves of the $[M+3H]^{3+}$, $[M+Cu+3H]^{5+}$, and $[M+Cu+H]^{3+}$ ions of cR6G-tm**P1** (see Supplementary Figures 2a and 3), together with the fluorescence decay curves of the $[M+3H]^{3+}$ and $[M+Cu+3H]^{5+}$ ions of rh110-tm**P2**, rh110-tm**P3**, and rh110-tm**P4**,[1] showed a shortened lifetime for Cu-bound species.

In the tmFRET model peptides, Cu^{2+} was found to be bound with the histidine close to the N-terminal, which was confirmed by molecular dynamics (MD) simulation and collision-induced

dissociation (CID).[1] Surface-induced dissociation (SID) experiments, which generally produce compact and native-like subcomplex fragments of multiply charged proteins, was also conducted for the tmFRET model peptides.[2] Compared with CID, SID provides better energy transfer in theory. Therefore, SID avoids a slow heating effect of ions in CID and results in a less Cu^{2+} rearrangement during fragmentation process. Overall, SID results also confirmed similar binding site for Cu^{2+}.[3]

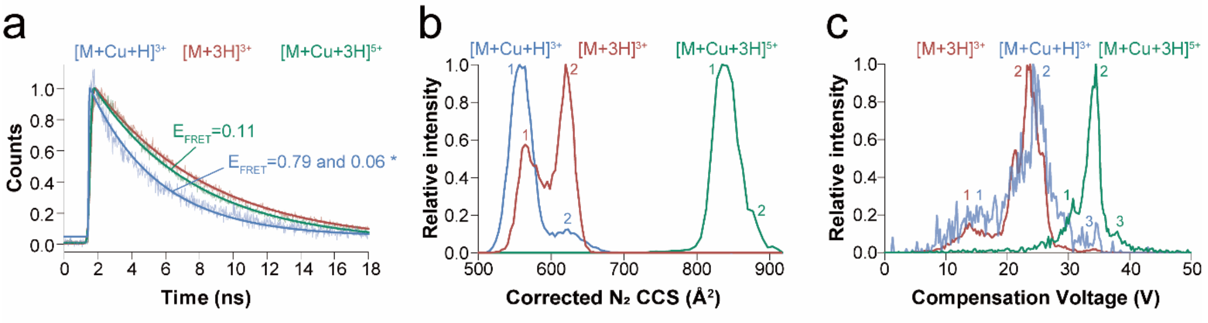

Supplementary Figure 2. **a**, Fluorescence decay curves of the $[M+3H]^{3+}$, $[M+Cu+H]^{3+}$, and $[M+Cu+3H]^{5+}$ ions of cR6G-tm**P1**. FRET efficiencies (E_{FRET}) were calculated based on the lifetime values acquired from single- or double-exponential fits (marked with *). **b**, Traveling wave ion mobility (TWIM) CCS distributions of cR6G-tm**P1** ions. **c**, DMS ionograms of cR6G-tm**P1** ions in a dispersion voltage (DV) of 4.5 kV and isopropanol (IPA) concentration of 0.3 mol % in the carrier gas.

Supplementary Note 2.1. Lifetime measurements and distance estimation from FRET efficiency (*E*)

The estimated r_{DA} from the FRET efficiencies of cR6G-tm**P1** (from Supplementary Figures 2a and 3) in the gas phase are discussed in the manuscript. The distance increasement indicated an expanded helical structure with increasing charge state, which is likely due to Coulomb repulsion. However, previous MD simulations of rh110-tm**P2**, -tm**P3**, and -tm**P4** using AMBER ff14SB force field showed a broad radial distribution of donor-acceptor distances.[1] This indicated the possibility of multiple conformations in specific charge states. Multiple conformations would lead to convoluted fluorescence decay curve, in which single-exponential fit is usually insufficient. Moreover, fluorescence decay curves acquired in the gas phase are typically in a limited quality as the low ion density and small fluorescence collection angle. Therefore, the resulting fluorescence decay curve is challenging to be analyzed even with a multi-exponential fits. Determination of distances based on FRET (or tmFRET) could be more accurate if multiple species are separated or in combination with IM-MS. Therefore, we hypothesized that an online coupling with DMS separation to fluorescence spectroscopy could potentially disentangle the complexity of data set.

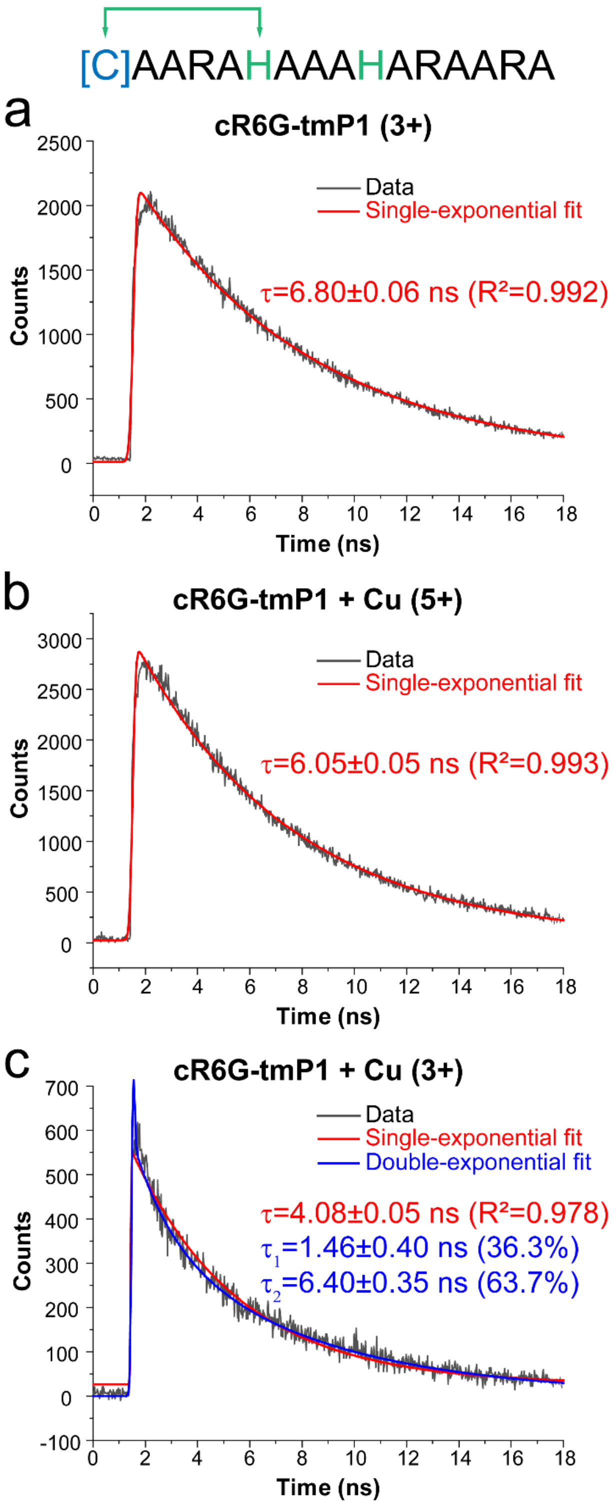

Supplementary Figure 3. Fluorescence decay curves of the (**a**) $[M+3H]^{3+}$, (**b**) $[M+Cu+3H]^{5+}$, and (**c**) $[M+Cu+H]^{3+}$ ions of cR6G-tmP1 with λ_{ex} = 460 nm, P = 10mW. $[M+Cu+3H]^{5+}$ signal was acquired with 3*3600 s of fluorescence collection, another two ions were acquired with 3*600 s of fluorescence collection. The fluorescence decay curves were fitted with a single-exponential modified Gaussian function (red) or double-exponential fit (blue). The arrow shows the tmFRET pathway between the position of labeled dye and where Cu^{2+} is likely to be bound.

Supplementary Table 1. Results obtained from a double-exponential fit using Decay Fit and a pre-defined instrument response function (IRF) by fluorescence decay curves in Supplementary Figures 2a and 3.

Peptide	T1 (ns)	T2 (ns)	rel. contribution T1
cR6G-tm**P1** (3+)	6.84	6.84	0.27
cR6G-tm**P1** + Cu (5+)	5.97	25.00	0.97
cR6G-tm**P1** + Cu (3+)	1.46 ± 0.40	6.40 ± 0.35	0.36 ± 0.05

Supplementary Note 2.2. IM-MS measurements

IM-MS measurements were conducted on the tmFRET model peptides to acquire their arrival time distribution (ATD) and further calculate the corresponding CCS distributions. TWIM CCS distributions are presented in Supplementary Figures 4a-b, together with a comparison of the DMS ionogram (Supplementary Figures 4c-d). The results showed the orthogonality between these two different types of ion mobility devices. Corrected N_2 CCS values were calculated by using poly-DL-alanine in the Major Mix IMS/Tof calibration kit as the calibrant, and calibration was fitted by the linear fit method. Datasets with poor quality are removed from the ionogram and TWIM CCS distributions. The corrected N_2 CCS values for different charge states of the tmFRET model peptides are shown in Supplementary Table 2. The CCS values of all peptide ions (i.e., the protonated ions and the Cu^{2+} complexes) increase as the protein shows unfolding due to the charge states increase. Only one conformer was identified in the TWIM spectra for some of the ions, while some other ions exhibit multiple conformers. For example, two conformations were identified for the $[M+3H]^{3+}$ ions of cR6G-tm**P1**, rh110-tm**P2**, and rh110-tm**P3**. This could be related to the unfolding upon gaining of additional charges. Low charge states (i.e., 2+) are often referred to "native" state in native MS, and peptide ions are likely to retain their folded structure. As the charge state increases, peptide ions quickly unfold (likely 3+ charge state) and stabilize into one or two conformations (4+ and 5+ charge states). As shown in Supplementary Figure 5, slightly different trends of CCS increment from 2+ to 4+ (or 5+ for cR6G-tm**P1**) upon Cu^{2+} bound were observed for these peptides. In general, the trend of cR6G-tm**P1** protonated species increase linearly, while rh110-tm**P2**, rh110-tm**P3**, and rh110-tm**P4** of dramatically from 2+ to 3+ charge state, then flattened from 3+ to 4+. Specifically, the $[M+2H]^{2+}$ ion of cR6G-tm**P1** has the CCS compared

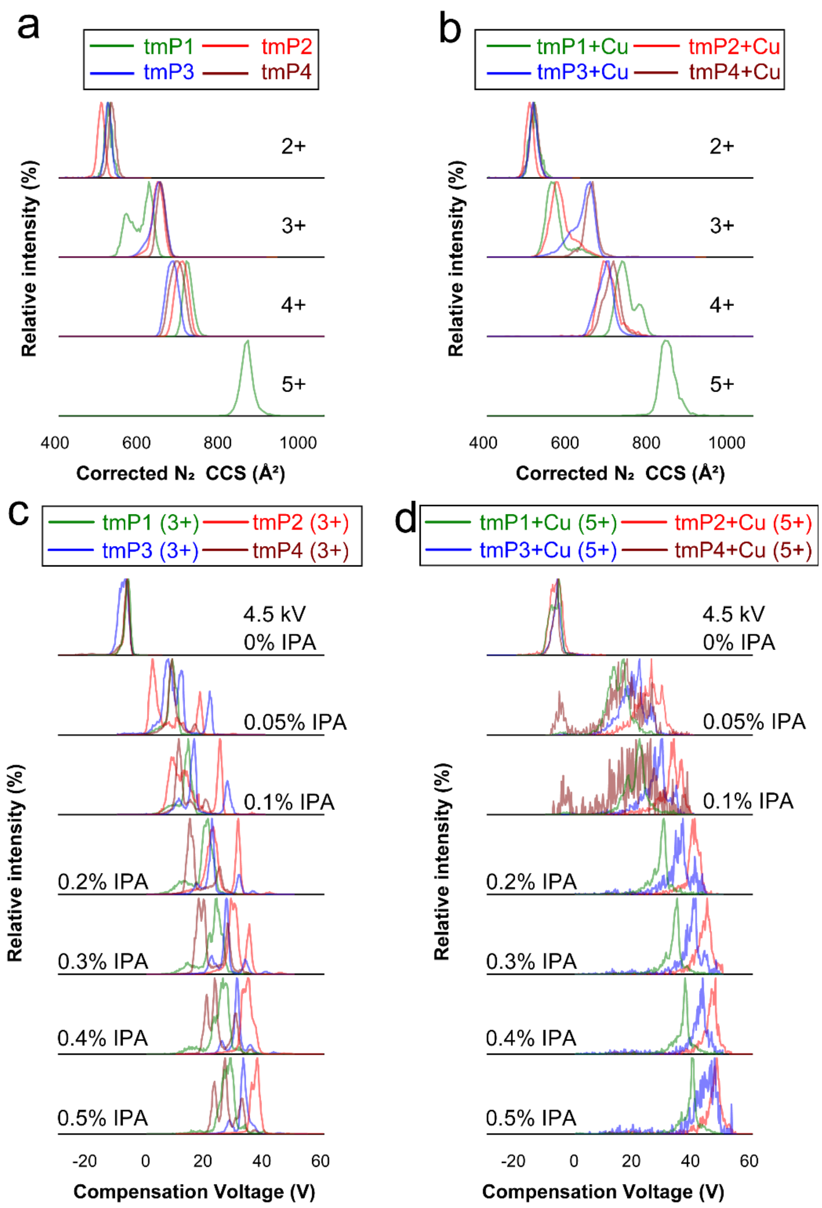

Supplementary Figure 4. DMS and TWIM separation of the tmFRET model peptides. (**a&b**) Normalized TWIM CCS distributions of the tmFRET model peptides. Protonated ($[M+nH]^{n+}$, n=2 to 5) and copper adducted ions ($[M+Cu+nH]^{(2+n)+}$, n=0 to 3) are separated with a wave height of 7.5 V. (**c&d**) Normalized DMS ionogram of the $[M+3H]^{3+}$ and $[M+Cu+3H]^{5+}$ ions of the tmFRET model peptides. Dispersion voltage was 4.5 kV. IPA concentration (0 to 0.5 mol %) was modulated in stepwise to steer the separation. Datasets with poor quality (i.e., signal intensity) were removed from the DMS ionogram and TWIM CCS distributions.

with $[M+2H]^{2+}$ of rh110-tm**P3**, while CCS of $[M+4H]^{4+}$ of cR6G-tm**P1** increased to 720 Å^2, much higher than $[M+4H]^{4+}$ of rh110-tm**P3** (678 Å^2). $[M+2H]^{2+}$ of rh110-tm**P2** (505 Å^2) is the most compact among rh110-tm**P2**, rh110-tm**P3**, and rh110-tm**P4**, while $[M+4H]^{4+}$ of rh110-tm**P2** is the most unfolded (701 Å^2). In summary, IM-MS measurements provide insights into the structural

characteristics for different charge states and upon Cu^{2+} binding.

Due to the Cu^{2+} binding to the His-X_3-His motif, the compactness (folding and unfolding) of peptide ions also showed dramatical differences. For more native charge state (i.e., 2+), binding with Cu^{2+} had the lowest CCS value (i.e., most compact). In the 3+ charge state, the $[M+Cu+H]^{3+}$ ion of cR6G-tm**P1** and rh110-tm**P2** has lower CCS values compared with the $[M+3H]^{3+}$ ion, while the trend is reversed for rh110-tm**P3** and rh110-tm**P4**. Specifically, the $[M+Cu+H]^{3+}$ ion of rh110-tm**P2** (569 Å^2) is much smaller than the $[M+3H]^{3+}$ ion (643 Å^2). While the $[M+Cu+H]^{3+}$ ion of rh110-tm**P4** (695 Å^2) is much larger than the $[M+3H]^{3+}$ ion (648 Å^2). The $[M+Cu+H]^{3+}$ ion of cR6G-tm**P1** (565 Å^2) is also much larger than the $[M+3H]^{3+}$ ion (623 Å^2). The peptide conformation in 4+ and 5+ species are rather random for each of the peptide. In summary, Cu^{2+} binding to presented model peptides would affect the conformation of the compound (either folded or unfolded), consequently changing its CCS value.

Supplementary Table 2. Experimental N_2 CCS values for the tmFRET model peptide ions. Multiple peak fitting using a Gaussian peak function was applied to fit the TWIM CCS distributions. The linear fit CCS values of the major conformer in each species are in bold. The average linear fit CCS and relative standard deviation was calculated from three replicates with wave heights of 7.0 V, 7.5 V, and 8.0 V in the TWIM separations.

Ions	Assignments	m/z	Peak number	linear fit CCS (Å²)	RSD (%)
cR6G-tm**P1**	$[M+2H]^{2+}$	1099.0793	1	**521.4**	0.1
	$[M+Cu]^{2+}$	1130.0355	1	**513.9**	1.2
	$[M+3H]^{3+}$	733.0640	1	574.3	1.8
			2	598.1	-
			3	**622.6**	0.4
	$[M+Cu+H]^{3+}$	753.6912	1	**565.3**	1.5
			2	628.7	1.7
	$[M+4H]^{4+}$	550.0521	1	**720.0**	0.8
	$[M+Cu+2H]^{4+}$	565.5279	1	**737.2**	1.0
			2	779.7	0.7
	$[M+5H]^{5+}$	440.2447	1	**858.4**	1.2
	$[M+Cu+3H]^{5+}$	452.6232	1	**829.6**	0.9
rh110-tm**P2**	$[M+2H]^{2+}$	1043.0625	1	**504.5**	0.2
	$[M+Cu]^{2+}$	1074.0117	1	**504.3**	0.2
	$[M+3H]^{3+}$	695.7160	1	602.6	0.5
			2	**643.3**	0.4
	$[M+Cu+H]^{3+}$	716.3520	1	**568.8**	0.9

			2	615.5	0.9
	$[M+4H]^{4+}$	522.0361	1	**700.8**	0.9
	$[M+Cu+2H]^{4+}$	537.5095	1	**687.7**	0.7
rh110-tm**P3**	$[M+2H]^{2+}$	1043.0625	1	**521.3**	0.2
	$[M+Cu]^{2+}$	1074.0117	1	**512.6**	0.2
			2	516.3	-
	$[M+3H]^{3+}$	695.7160	1	611.8	1.2
			2	**646.6**	0.3
	$[M+Cu+H]^{3+}$	716.3520	1	615.2	0.8
			2	**650.5**	0.3
	$[M+4H]^{4+}$	522.0361	1	**678.3**	0.6
	$[M+Cu+2H]^{4+}$	537.5095	1	659.1	0.9
			2	**691.2**	0.8
rh110-tm**P4**	$[M+2H]^{2+}$	1043.0625	1	**529.6**	0.1
	$[M+Cu]^{2+}$	1074.0117	1	488.7	0.2
			2	**514.2**	0.1
	$[M+3H]^{3+}$	695.716	1	**648.0**	0.3
	$[M+Cu+H]^{3+}$	716.3520	1	610.9	0.5
			2	652.5	0.3
			3	**695.1**	-
	$[M+4H]^{4+}$	522.0361	1	**686.2**	0.8
	$[M+Cu+2H]^{4+}$	537.5095	1	**704.6**	0.5

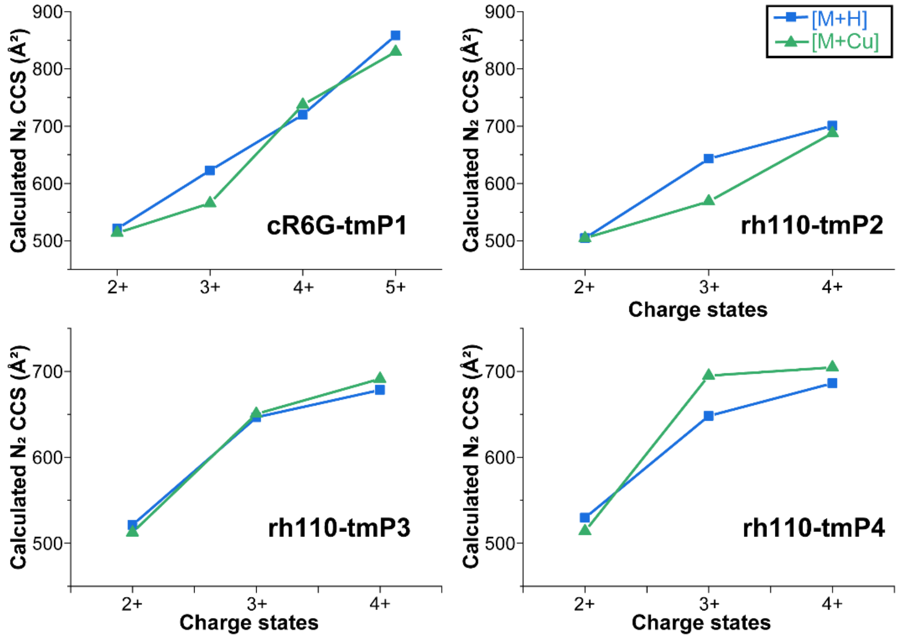

Supplementary Figure 5. Corrected N_2 CCS values for the four tmFRET model peptide ions. Only the major conformer in the ion mobility spectra for the Cu-free species (blue) and Cu-bound complexes (green) ions are plotted. Binding with Cu^{2+} induces alternation of peptide structure in the gas phase.

Supplementary Note 2.3. DMS experiments

DMS separations of four tmFRET model peptides were then conducted and results are presented in Supplementary Figures 6-14. When no gas modifier was applied, poor separation was achieved. Isopropanol (IPA), serving as a common gas modifier, was then doped into the carrier gas. Peptide ions were shifted from negative compensation voltage (CV) to positive CV due to dynamic clustering/declustering mechanisms.[4,5] As shown in Supplementary Figure 6, the $[M+3H]^{3+}$ and $[M+Cu+3H]^{5+}$ ions of each peptide were well-separated, as the $[M+Cu+3H]^{5+}$ ions were in a more positive CV. The results indicated that Cu^{2+} binding to the model peptides affects the conformation of the compound, thus affecting the interaction between gas modifier and the ion.[6] As rh110-tmP2, rh110-tmP3, and rh110-tmP4 are isomeric compounds, it is also worth to mention that these isomeric $[M+3H]^{3+}$ and $[M+Cu+3H]^{5+}$ ions could also be partially separated by DMS. Two to six peaks (or conformations) could be identified from the ionograms of 0.05 and 0.4 mol % IPA (Supplementary Figure 6). Different concentrations of IPA results in different separation behaviors for each charge state and compound, which is likely depending on the rate of dynamic clustering/declustering and ion-modifier binding energy.[7]

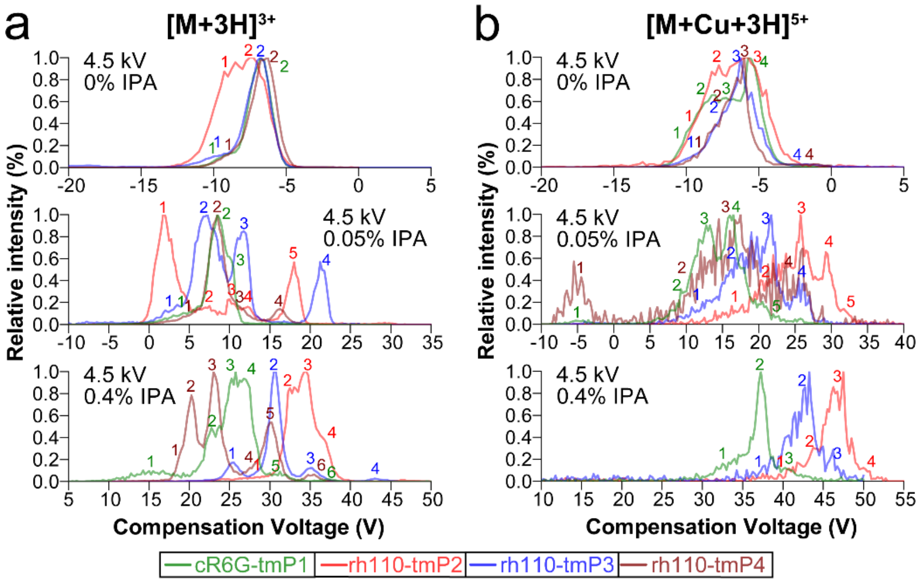

Supplementary Figure 6. Normalized DMS ionograms of, (a) $[M+3H]^{3+}$, and (b) $[M+Cu+3H]^{5+}$ of the tmFRET model peptides in 0, 0.05, and 0.4 mol % IPA. Dispersion voltage was 4.5 kV. Identified peaks in the ionogram are labeled. $[M+3H]^{3+}$ and $[M+3H+Cu]^{5+}$ ions displayed dramatically different separation behavior and allowed isomer separation.

Charge state separations of 2+ to 5+ of the tmFRET model peptides (see Supplementary Figures 7-14) are also observed, which is especially helpful for studying laser-induced fluorescence in an electrospray plume as it enables selection of specific charge state for investigation.[8] The shape of the ionograms (i.e., conformational distributions) were also dramatically different for different charge states. Compared with low charge state ions, higher charge state ions tend to occupy larger CV positions, as more charges allow a higher kinetic energy gain from asymmetric field and hence amplifying the mobility difference. Further, extra charging sites allow more binding/interaction sites for gas modifier molecules, which also leads to larger mobility difference in low and high field.

There is no direct correlation between the peptide unfolding (due to charge increasement) and the number of peaks in DMS ionogram. For example, for rh110-tmP3, $[M+2H]^{2+}$ has four peaks, $[M+3H]^{3+}$ has four peaks, $[M+4H]^{4+}$ has three peaks, and $[M+5H]^{5+}$ has two peaks in the resulting ionogram. Some minor peak of low charge states "coincidentally" has the same CV position with high charge states. For example, the more positive CV peak of the $[M+3H]^{3+}$ ion of rh110-tmP2 (CV=~30 V) overlapped with the $[M+4H]^{4+}$ ion peak in 0.2 mole % IPA. This is likely due to the charge stripping effect from ions with high to low charge states during the ion transmission in the interface of DMS and mass spectrometer.

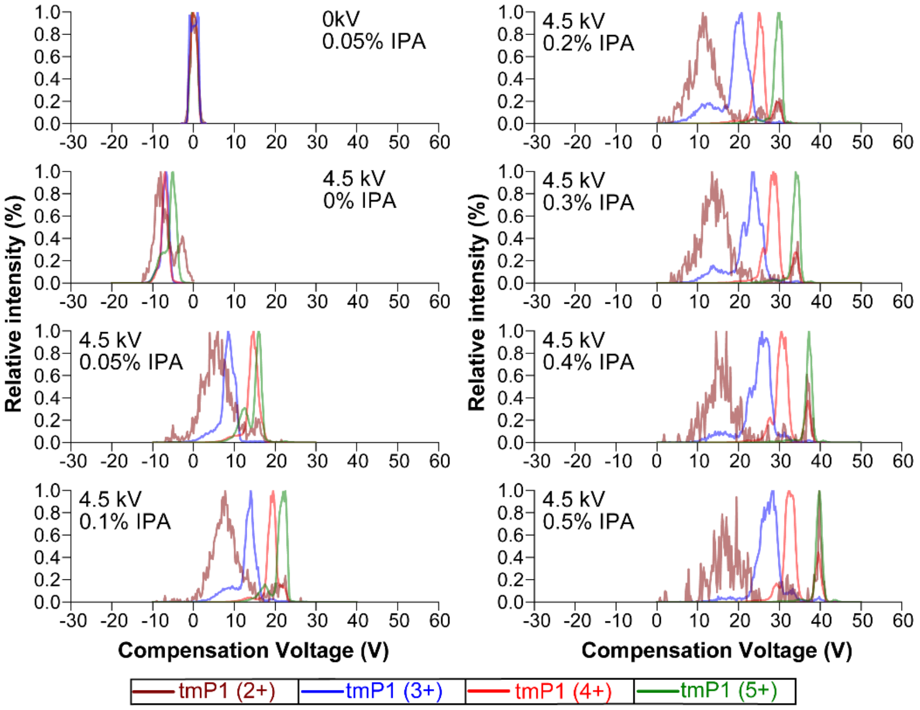

Supplementary Figure 7. Normalized DMS ionograms of the protonated cR6G-tmP1 ions ($[M+nH]^{n+}$, n=2 to 5) in 0 to 0.5 mol % IPA.

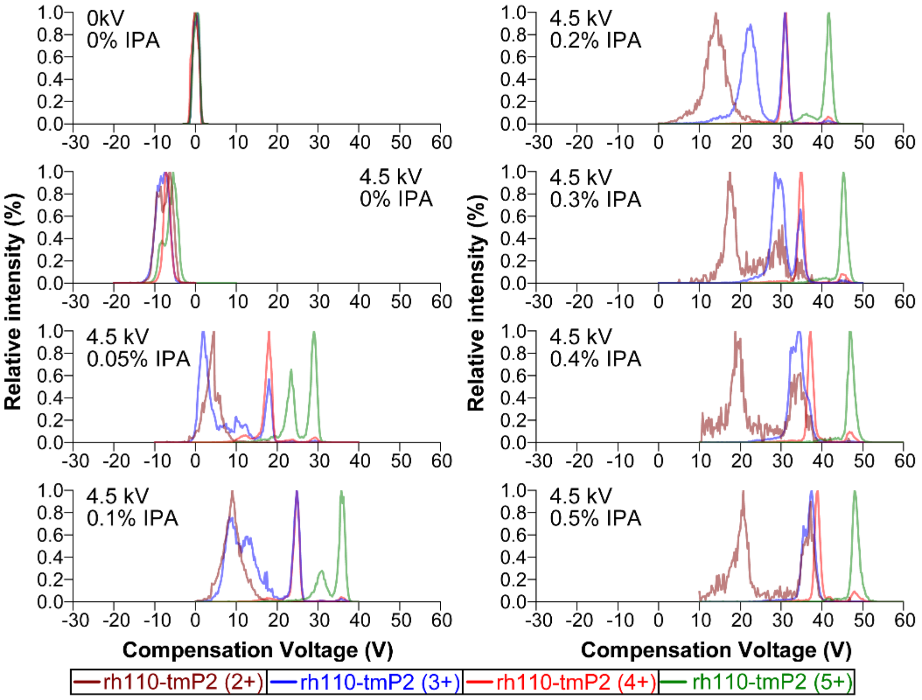

Supplementary Figure 8. Normalized DMS ionograms of the protonated rh110-tmP2 ions ($[M+nH]^{n+}$, n=2 to 5) in 0 to 0.5 mol % IPA.

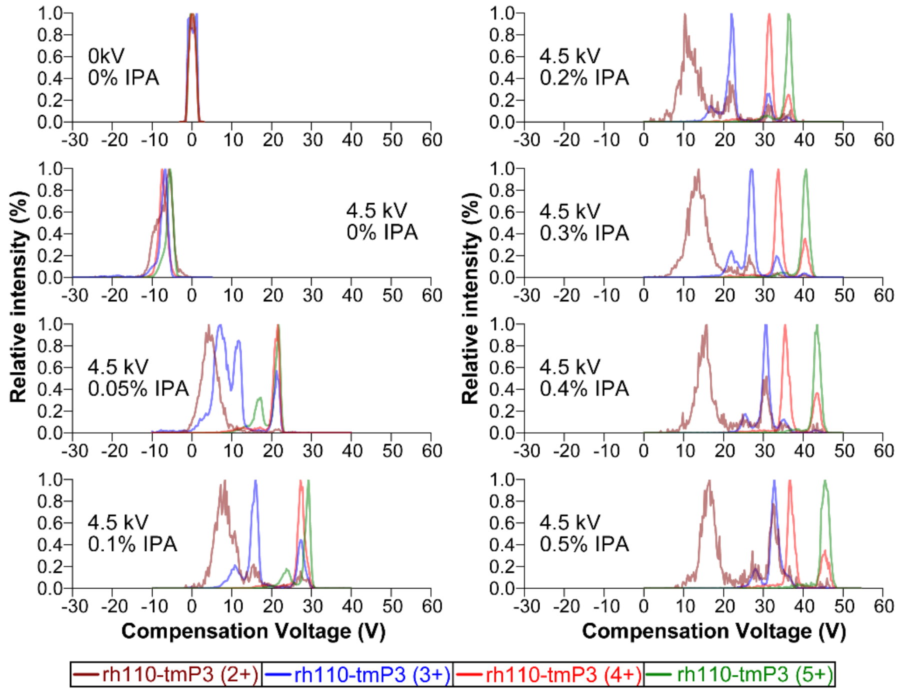

Supplementary Figure 9. Normalized DMS ionograms of the protonated rh110-tmP3 ions ($[M+nH]^{n+}$, n=2 to 5) in 0 to 0.5 mol % IPA.

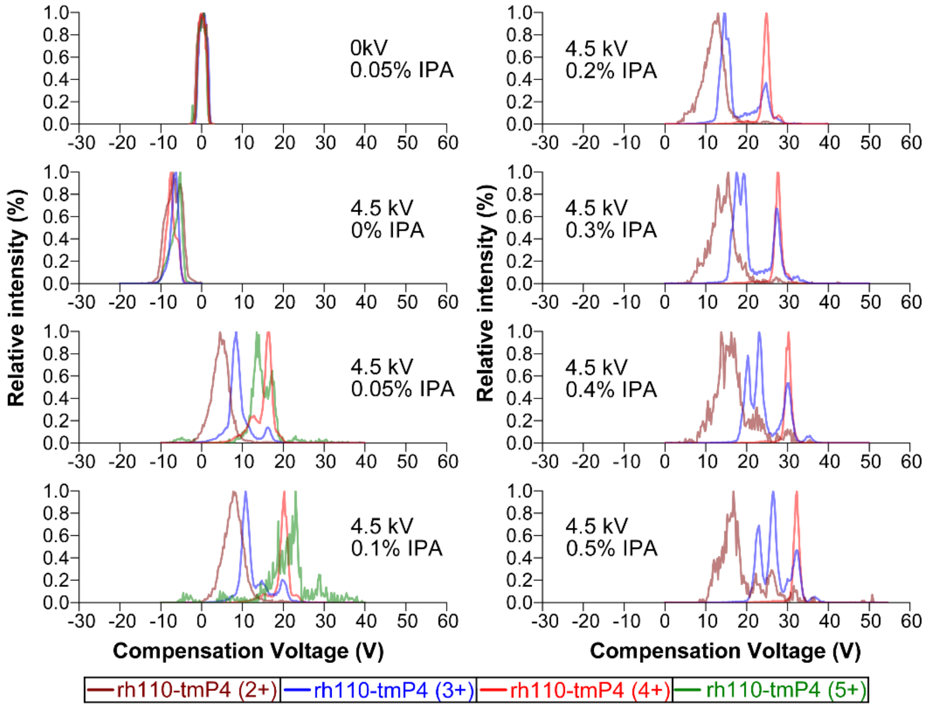

Supplementary Figure 10. Normalized DMS ionograms of the protonated rh110-tmP4 ions ($[M+nH]^{n+}$, n=2 to 5) in 0 to 0.5 mol % IPA.

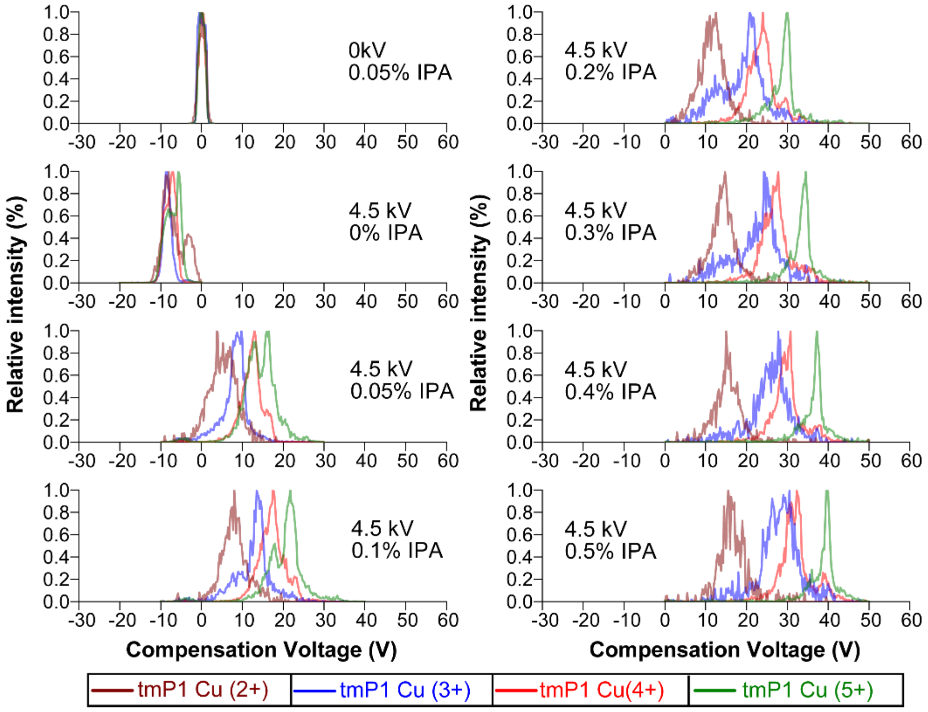

Supplementary Figure 11. Normalized DMS ionograms of the copper adduced cR6G-tmP1 ions ($[M+Cu+nH]^{(2+n)+}$, n=0 to 3) in 0 to 0.5 mol % IPA.

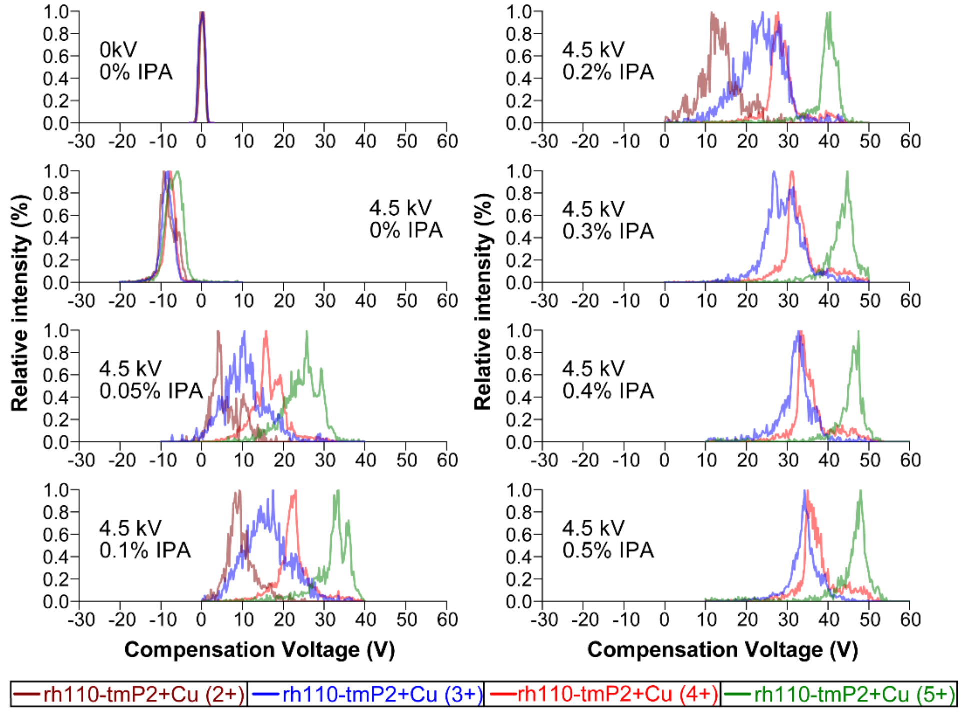

Supplementary Figure 12. Normalized DMS ionograms of the copper adduced rh110-tmP2 ions ($[M+Cu+nH]^{(2+n)+}$, n=0 to 3) in 0 to 0.5 mol % IPA.

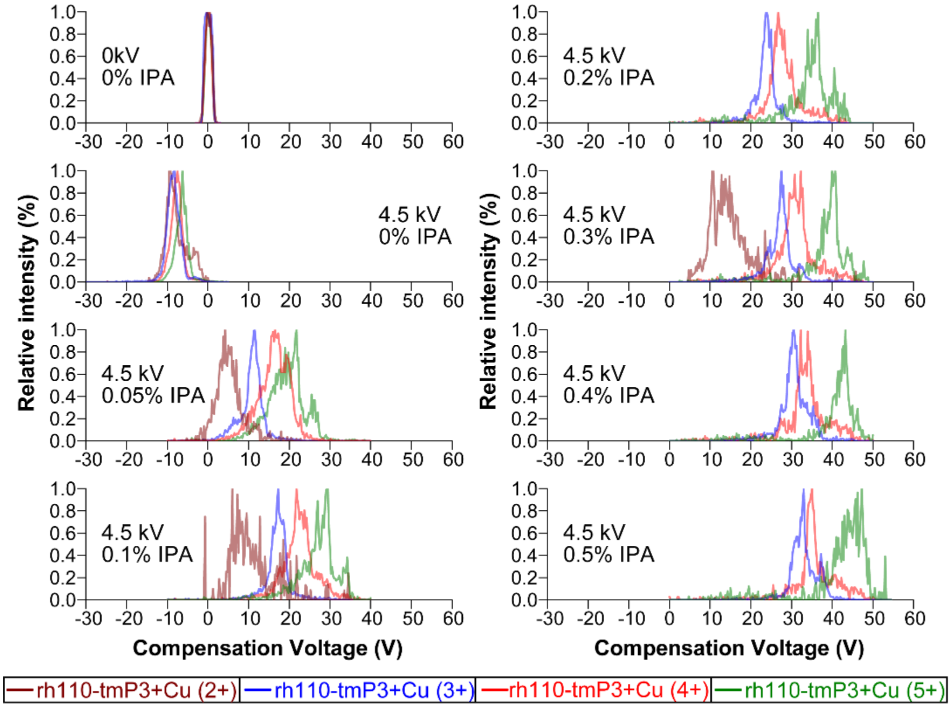

Supplementary Figure 13. Normalized DMS ionograms of the copper adduced rh110-tmP3 ions ($[M+Cu+nH]^{(2+n)+}$, n=0 to 3) in 0 to 0.5 mol % IPA.

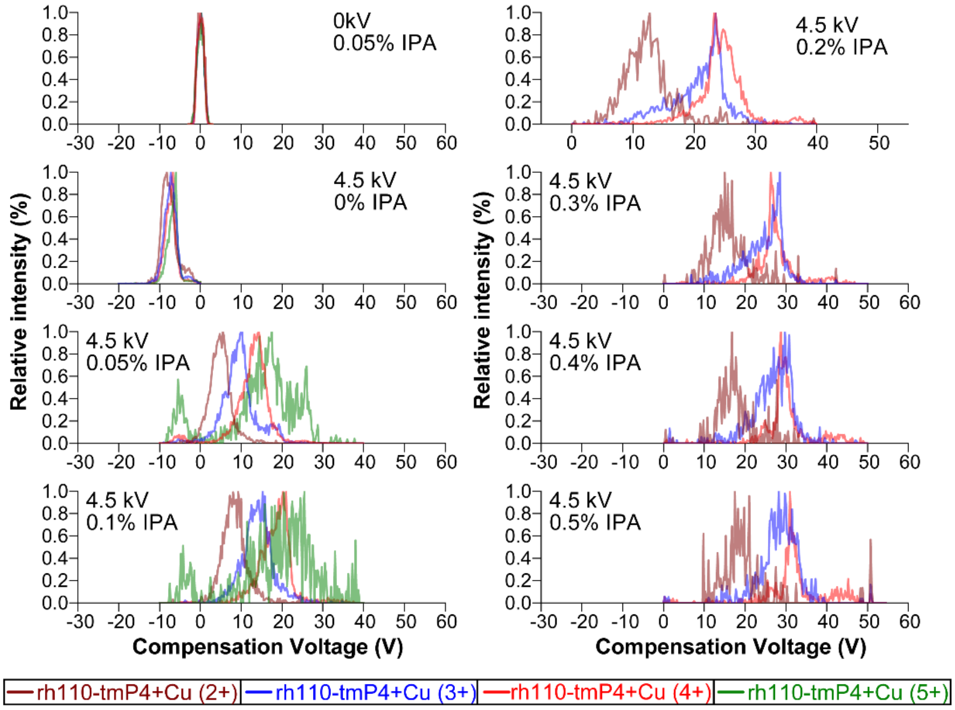

Supplementary Figure 14. Normalized DMS ionograms of the copper adduced rh110-tmP4 ions ($[M+Cu+nH]^{(2+n)+}$, n=0 to 3) in 0 to 0.5 mol % IPA.

Supplementary Note 2.4. Summary

In the DMS experiments, most of the $[M+3H]^{3+}$ ion of tmFRET model peptides showed sufficient intensity, which enables fluorescence spectroscopic experiments on each DMS separated conformer. Further, the results also showed that multiple conformations exist for $[M+Cu+3H]^{5+}$ ions, though the minor peaks (i.e., conformers) were in relatively low signal intensity (i.e., lower than $2*10^{7}$ intensity). However, low abundance conformer with large CCS value are also likely to contribute to the fluorescence decay curve as they are more unfolded (i.e., less quenched by acceptor fluorophore). On the other hand, the complementary TWIM separations were conducted. Since the developed tmFRET method relies on the lifetime difference between $[M+3H]^{3+}$ and $[M+Cu+3H]^{5+}$ ions, negligence of multiple conformations and the structure changes upon binding to Cu^{2+} leads to an inaccurate estimation of FRET efficiencies (E). Therefore, adding the distance measurement to the CCS information improves the selectivity as a specific CCS value of an ion is a global shape (or size) parameter and may not limit to only one conformation. FRET or tmFRET, which provides intramolecular distance, is potentially capable to distinguish ions of different conformation with similar CCS. Therefore, we hypothesized that the computational simulation can be guided for better determination of the gas-phase ion structures.

DMS separation, which is proved to be a complementary technique to IM-MS and FRET or tmFRET, represents the cluster formation properties between a charged ion and (one or several) neutral gas modifier molecules. Even though the ion-molecular interaction varies between analytes, the binding energy and possible solvation sites available for binding of gas modifier molecules could be calculated for each candidate conformer. Therefore, DMS results were incorporated to the selection of candidate structures in the gas phase, together with IM-MS and FRET-based methods.

Supplementary Note 3. Differential ion mobility spectrometry (DMS) instrumentation and experiments

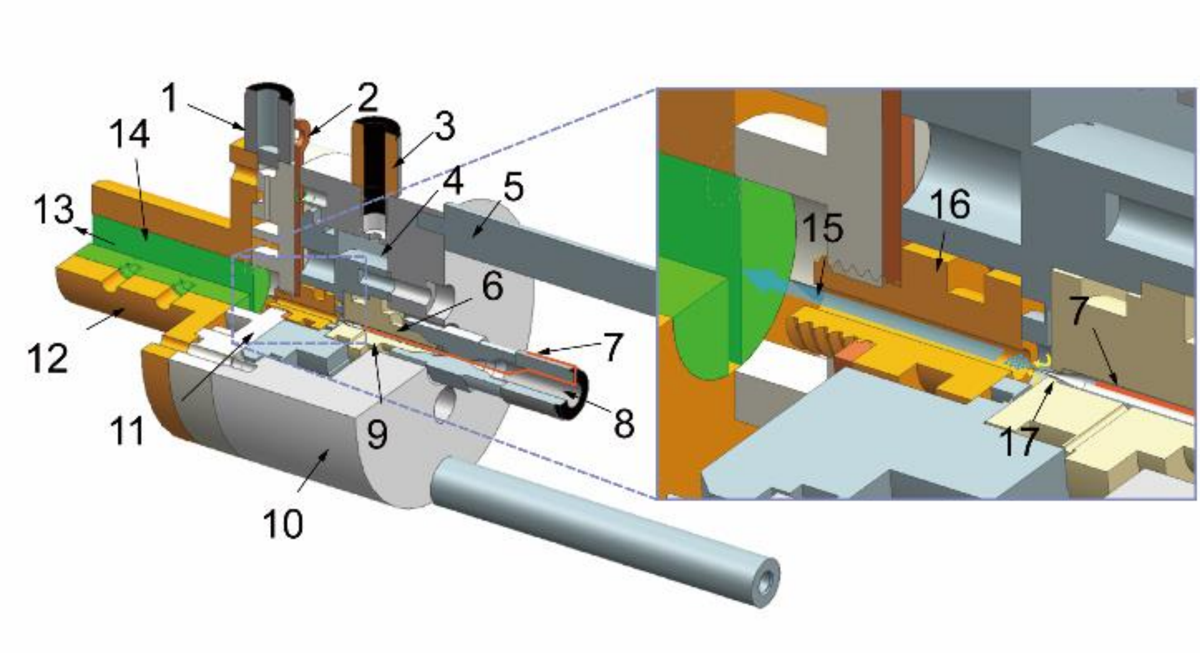

Supplementary Figure 15. Schematic of the nanoESI DMS device. 1. Auxiliary gas inlet; 2. Counter electrode wire; 3. Nebulizer gas inlet; 4. Sprayer holder; 5. Adaptor to translation stages; 6. NanoESI glass capillary; 7. Platinum wire; 8. Quick fitting for backing pressure (N_2); 9. nanoESI sprayer; 10. nanoESI chamber (i.e., housing 1); 11. Circular channel (i.e., housing 2); 12. DMS chamber (housing 3); 13. Transfer to MS; 14. DMS electrodes; 15. Skimmer; 16. Counter electrode; 17. nanoESI. The 3D model was created with the help of NX 11.0 software (Siemens Software).

Supplementary Note 4. DMS, IMS and fluorescence spectroscopic measurements of singly labelled α-helical peptide

Supplementary Note 4.1. HPLC separation

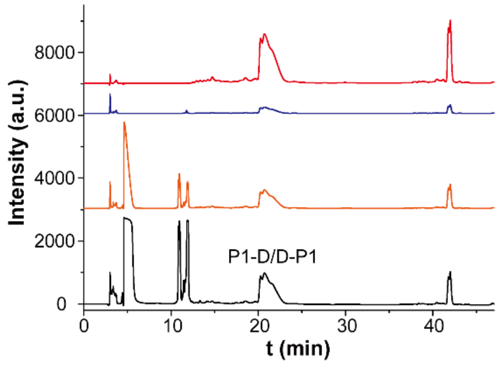

Supplementary Figure 16. HPLC purification of two labelling isomers of P1-Atto 532. UV/VIS absorption spectroscopy was set at wavelengths of 220 nm (black), 235 nm(orange), 280 nm (blue), and 532 nm (red) for detection. The denotation X-P1-Y refers to the dye position after label. X refers to the N-terminal and Y to the C-terminal. A gentle gradient of ACN concentration of 23.8% to 25.0% was set between 6 and 31 minutes.

Supplementary Note 4.2. DMS coupled with gas-phase fluorescence spectroscopy and dissociation methods

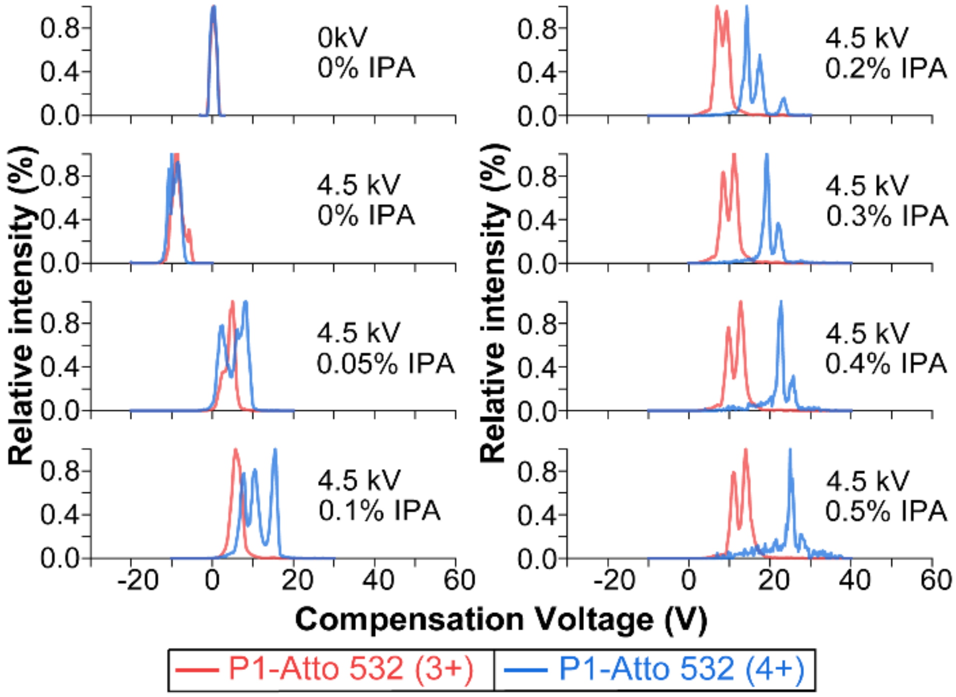

Supplementary Figure 17. Normalized DMS ionograms of the $[M+3H]^{3+}$ (red) and $[M+4H]^{4+}$ (blue) ions of P1-Atto 532 in the presence of isopropanol (IPA) as the gas modifier in the carrier gas.

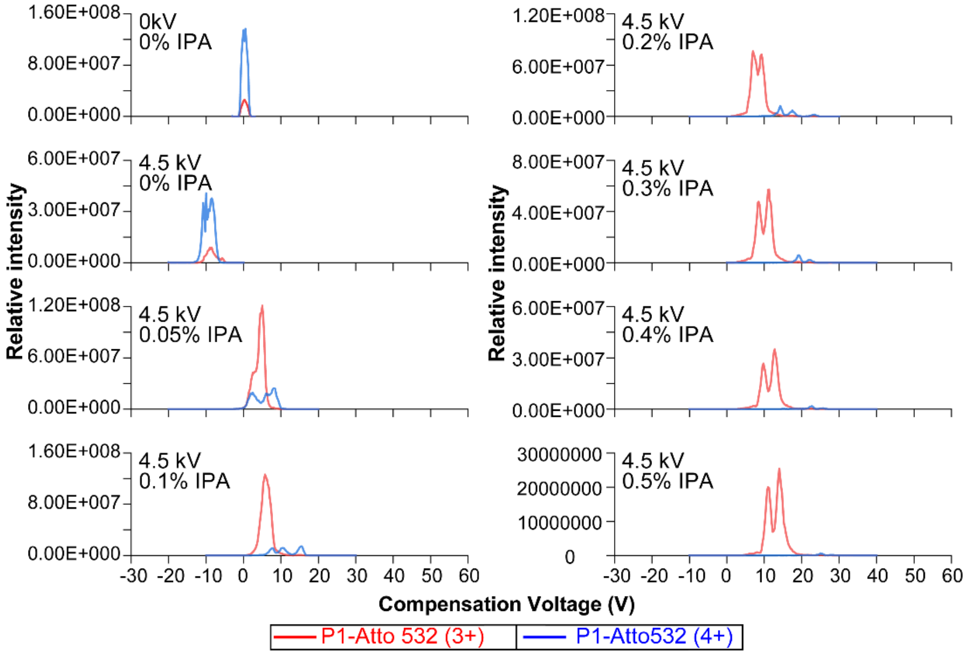

Supplementary Figure 18. DMS separation of the $[M+3H]^{3+}$ and $[M+4H]^{4+}$ ions of P1-Atto 532 in 0 to 0.5 mol % IPA. Dispersion voltage was 4.5 kV. A higher concentration of IPA significantly reduces the signal intensity and broadens the peak, which is likely due to a charge stripping effect from $[M+4H]^{4+}$ to $[M+3H]^{3+}$ in the presence of IPA, and competitive ionization.[7]

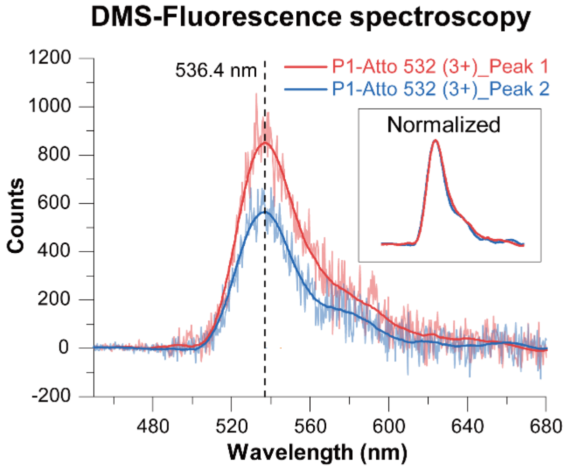

Supplementary Figure 19. Gas-phase fluorescence emission spectra of the $[M+3H]^{3+}$ ions of P1-Atto 532 after DMS with λ_{ex} = 480 nm, P = 10mW, and 300 s of fluorescence collection. In DMS, two peaks were separated with CV values of ~8 V and ~10.25 V.

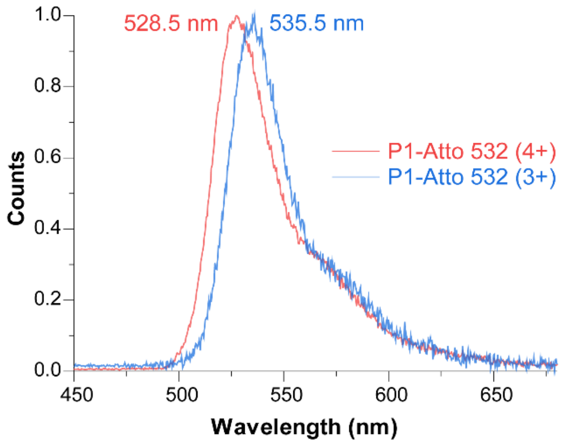

Supplementary Figure 20. Gas-phase fluorescence emission spectra of the $[M+3H]^{3+}$ and $[M+4H]^{4+}$ ions of P1-Atto 532/Atto-532-P1 isomers with λ_{ex} = 480 nm, P = 10mW, and a fluorescence collection time of 720 s. Comparison of the emission spectra obtained for the 3+ and 4+ charge states revealed a blue shift of the emission maximum from 535.5 nm to 528.5 nm. The altered emission properties are likely due to changes in the solvation environment of the dye or the additional charge interacting with the fluorophore's dipole.

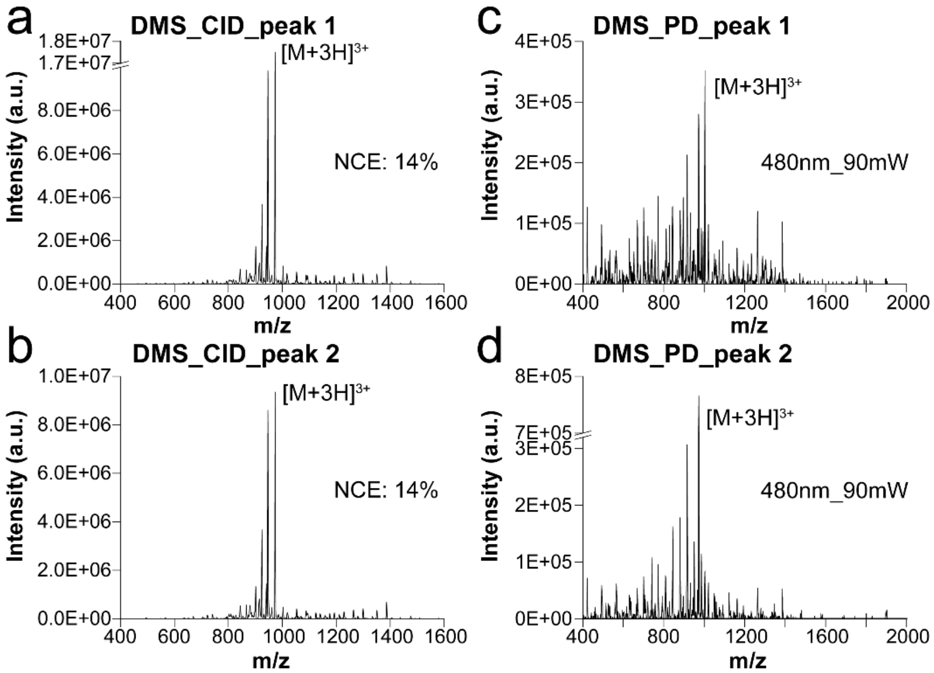

Supplementary Figure 21. (**a&b**) CID mass spectra of the $[M+3H]^{3+}$ ions of P1-Atto 532 after DMS separation with ~0.3 mol % IPA as the gas modifier. Normalized collision energy (NCE) was 14%. An isolation window of 10 Th was used in the modified QIT MS. (**c&d**) Photodissociation (PD) mass spectra of the $[M+3H]^{3+}$ ions of P1-Atto 532 after the DMS separation with λ_{ex} = 480 nm, P = ~90mW. Assignment of peak 1 and peak 2 are referring to Figure 2a in the manuscript.

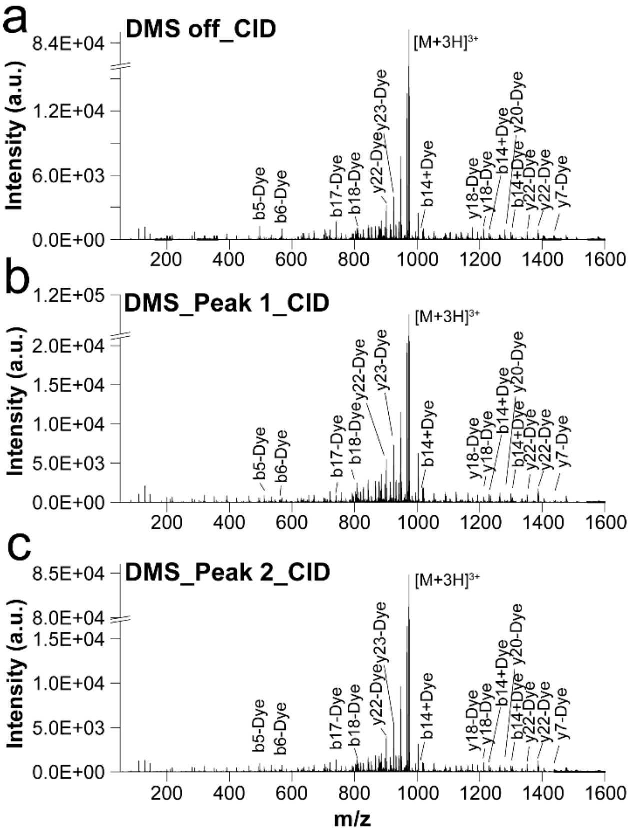

Supplementary Figure 22. CID mass spectra of the $[M+3H]^{3+}$ ions of P1-Atto 532, with and without the DMS separation. In DMS, ~0.3 mol % IPA was used as the gas modifier. Collision energy was set as 34 eV in the Synapt G2-S QTOF-MS. Assignment of peak 1 and peak 2 are referring to Figure 2a in the manuscript.

Supplementary Note 4.3. IM-MS measurements

Supplementary Table 3. The experimental N_2 CCS values for P1-Atto 532 ions. Multiple peak fit with Gaussian peak function was applied to fit all of the ion mobility spectra to define the peak number. The linear fit CCS value of the major component in each ion mobility spectrum is bolded. Relative standard deviation (RSD) was calculated from three replicates with wave heights of 7.0 V, 7.5 V, and 8.0 V in the TWIM separations.

Ions	Assignments	m/z	Peak number	linear fit CCS (Å²)	RSD (%)
P1-Atto 532	$[M+3H]^{3+}$	973.7648	1	638.4	0.3
			2	661.2	0.3
			3	691.3	0.5
			4	**723.7**	0.3
	$[M+3H]^{4+}$	730.5754	1	**757.5**	0.7

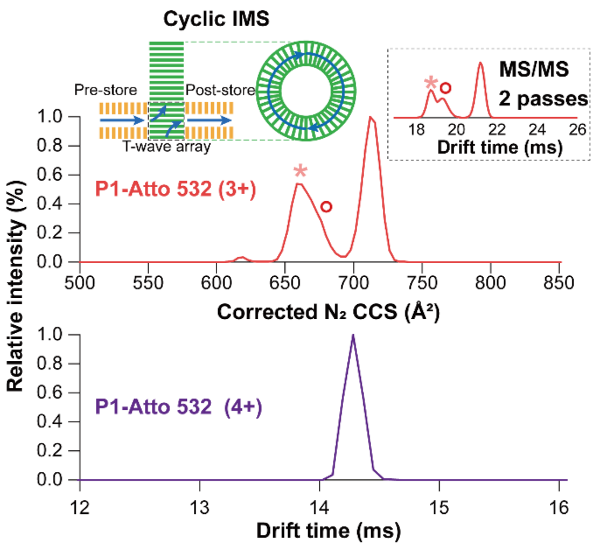

Supplementary Figure 23. Ion mobility spectra of the $[M+3H]^{3+}$ and $[M+4H]^{4+}$ ions of P1-Atto 532 in the cIM. Inset: 2 passes of cIM separation in MS/MS mode, with the $[M+3H]^{3+}$ ion isolated. The ratios of the four conformers were slightly different between experiments, which is dependent on the ratios of isomers collected from different HPLC batches.

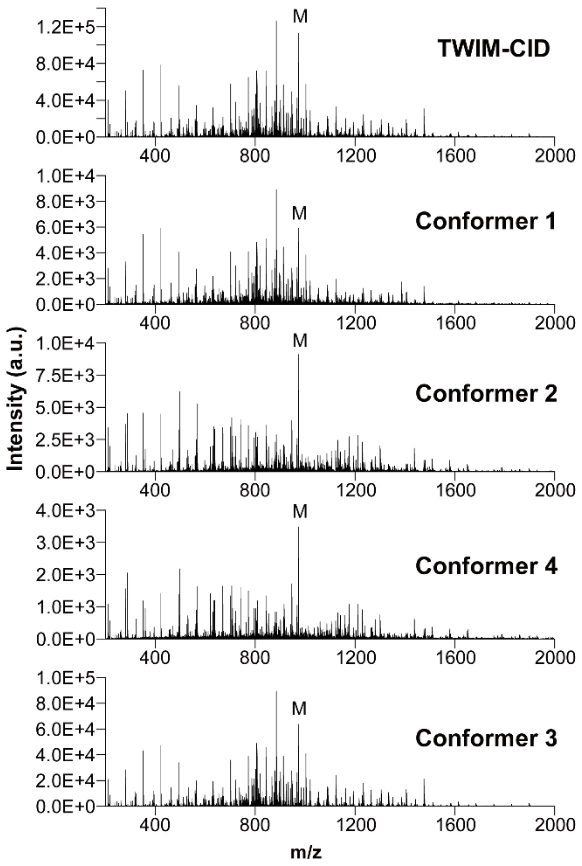

Supplementary Figure 24. CID mass spectra of the $[M+3H]^{3+}$ ions of P1-Atto 532 after the TWMS separation. Quadrupole isolation resolution was set to 5. 44 eV was used in the transfer region of the Triwave, which is after the TWIM separation. TWIM separation was assumed not to be affected. Conformer 1 and 3 also showed high ratio of y ions, while conformer 2 and 4 showed high ratio of b ions (Supplementary Tables 5-8). The difference is likely driven by a more favorable charging in the basic side chains of amino acids further away from the labelling dye (likely singly charge) due to Coulomb repulsion. The relatively low ratio of y-Atto 532 ions for conformers 1 and 3, and Atto 532 ions-b ions for conformers 2 and 4 are likely due to partially overlapped peaks in the CCS distributions.

Supplementary Table 4. Summary of the overall CID fragment ions from the TWIM-CID experiments. Precursor ion is the $[M+3H]^{3+}$ ions. 44 eV was used in the transfer region of the Triwave.

Assignment	Charge state	Theo. m/z	Expt. m/z	Error (ppm)	Int.	Rel. Int. (%)	Norm. to $[M+3H]^{3+}$ (%)
M+3H	3	973.4053	973.4084	-3.2	1.13E+05	89.7	100.0
b2	1	217.065	217.0596	24.9	6.97E+03	5.5	6.2
b3	1	288.102	288.097	17.4	1.62E+04	12.9	14.3
b4	1	359.139	359.1352	10.6	6.40E+03	5.1	5.7
b5	1	496.198	496.2007	-5.4	1.91E+04	15.2	16.9
b6	1	567.235	567.2331	3.3	1.66E+04	13.2	14.7
b7	1	638.272	638.275	-4.7	1.05E+04	8.3	9.3
b8	1	709.309	709.3035	7.8	8.41E+03	6.7	7.4
b9	1	846.368	846.3671	1.1	4.92E+03	3.9	4.4
b11	1	988.442	988.4489	-7.0	3.26E+03	2.6	2.9
b12	1	1059.479	1059.49	-10.1	4.16E+03	3.3	3.7
b13	1	1130.516	1130.514	2.0	7.31E+03	5.8	6.5
b14	1	1267.575	1267.586	-8.8	2.96E+03	2.3	2.6
b15	1	1338.613	1338.624	-8.0	1.54E+03	1.2	1.4
b16	1	1409.65	1409.646	2.6	1.64E+03	1.3	1.5
b17	1	1480.687	1480.69	-2.2	2.65E+03	2.1	2.3
b9	2	423.688	423.6861	4.5	2.13E+03	1.7	1.9
b10	2	459.206	459.2044	3.5	2.49E+03	2.0	2.2
b11	2	494.725	494.7236	2.8	3.86E+03	3.1	3.4
b12	2	530.243	530.2382	9.1	6.46E+03	5.1	5.7
b13	2	565.762	565.7576	7.8	5.30E+03	4.2	4.7
b14	2	634.291	634.2885	3.9	1.24E+03	1.0	1.1
b15	2	669.81	669.8137	-5.5	1.22E+03	1.0	1.1
b16	2	705.328	705.321	9.9	1.50E+04	11.9	13.3
b17	2	740.847	740.8419	6.9	1.38E+04	11.0	12.2
b18	2	809.376	809.3762	-0.2	9.47E+03	7.5	8.4
b19	2	844.895	844.8943	0.8	8.19E+03	6.5	7.2
b20	2	880.414	880.4107	3.7	7.60E+03	6.0	6.7
b21	2	915.932	915.9315	0.5	7.10E+03	5.6	6.3
y19	1	1654.834	1654.867	-19.6	1.77E+03	1.4	1.6
y18	1	1583.797	1583.791	4.0	3.38E+04	26.8	29.9
y17	1	1512.76	1512.78	-13.5	4.58E+03	3.6	4.1
y16	1	1441.722	1441.724	-1.4	8.45E+03	6.7	7.5
y15	1	1304.663	1304.676	-9.7	1.91E+04	15.2	16.9
y14	1	1233.626	1233.622	3.6	2.42E+04	19.2	21.4
y13	1	1162.589	1162.591	-1.9	2.00E+04	15.9	17.7
y12	1	1091.552	1091.56	-7.2	1.97E+04	15.6	17.4
y11	1	1020.515	1020.521	-5.7	2.86E+04	22.7	25.3

y10	1	883.456	883.4575	-1.7	2.65E+04	21.0	23.5
y9	1	812.419	812.422	-3.7	2.00E+04	15.9	17.7
y8	1	741.382	741.3766	7.3	1.58E+04	12.5	14.0
y7	1	670.345	670.3448	0.3	2.17E+04	17.2	19.2
y6	1	533.286	533.2886	-4.9	1.99E+04	15.8	17.6
y5	1	462.249	462.2499	-1.9	2.04E+04	16.2	18.1
y4	1	391.212	391.2103	4.3	1.67E+04	13.3	14.8
y3	1	320.175	320.1692	18.1	1.51E+04	12.0	13.4
y2	1	217.165	217.1625	11.5	1.40E+04	11.1	12.4
y24	2	1075.516	1075.537	-19.2	4.45E+03	3.5	3.9
y23	2	1003.006	1003.01	-4.0	5.75E+04	45.6	50.9
y22	2	967.487	967.4924	-5.6	2.31E+04	18.3	20.4
y21	2	931.969	931.9617	7.8	2.59E+04	20.6	22.9
y20	2	896.45	896.4552	-5.8	2.73E+04	21.7	24.2
y19	2	827.921	827.9127	10.0	1.60E+04	12.7	14.2
y18	2	792.402	792.4008	1.5	1.12E+04	8.9	9.9
y17	2	756.883	756.8743	11.5	9.63E+03	7.6	8.5
y16	2	721.365	721.3651	-0.1	1.87E+04	14.8	16.5
y15	2	652.835	652.8389	-6.0	9.58E+03	7.6	8.5
y14	2	617.317	617.3173	-0.5	6.68E+03	5.3	5.9
y13	2	581.798	581.799	-1.7	3.43E+03	2.7	3.0
y12	2	546.28	546.2814	-2.6	4.08E+03	3.2	3.6
y11	2	510.761	510.7571	7.6	1.04E+04	8.3	9.2
b2+dye-2H	1	984.2337	984.2533	-19.9	2.07E+04	16.4	18.3
b3+dye-2H	1	1055.271	1055.302	-29.5	8.56E+03	6.8	7.6
b5+dye-2H	1	1263.367	1263.393	-20.7	1.78E+04	14.1	15.8
b6+dye-2H	1	1334.404	1334.426	-16.7	1.51E+04	12.0	13.4
b7+dye-2H	1	1405.441	1405.47	-20.6	1.92E+04	15.2	17.0
b8+dye-2H	1	1476.478	1476.493	-10.5	3.07E+04	24.4	27.2
b9+dye-2H	1	1613.537	1613.57	-20.4	5.60E+03	4.4	5.0
b10+dye-2H	1	1684.574	1684.586	-7.5	3.37E+03	2.7	3.0
b11+dye-2H	1	1755.611	1755.632	-12.2	2.67E+03	2.1	2.4
b12+dye-2H	1	1826.648	1826.69	-22.9	2.81E+03	2.2	2.5
b13+dye-2H	1	1897.685	1897.711	-13.7	3.66E+03	2.9	3.2
b14+dye-2H	1	2034.744	2034.768	-11.7	6.61E+02	0.5	0.6
b15+dye-2H	1	2105.782	2105.799	-8.2	5.00E+02	0.4	0.4
b16+dye-2H	1	2176.819	2176.827	-3.7	3.12E+02	0.2	0.3
b17+dye-2H	1	2247.856	2247.853	1.4	4.19E+02	0.3	0.4
b5+dye-2H	2	632.187	632.2011	-22.3	1.87E+04	14.8	16.5
b6+dye-2H	2	667.7055	667.7138	-12.4	1.10E+04	8.7	9.7
b10+dye-2H	2	842.7905	842.8018	-13.4	4.10E+04	32.5	36.3
b11+dye-2H	2	878.309	878.3135	-5.1	3.98E+04	31.6	35.2
b12+dye-2H	2	913.8275	913.8452	-19.3	3.40E+04	27.0	30.1
b13+dye-2H	2	949.346	949.3547	-9.1	2.48E+04	19.7	21.9

b14+dye-2H	2	1017.876	1017.887	-10.9	1.74E+04	13.8	15.4
b15+dye-2H	2	1053.395	1053.402	-7.4	1.60E+04	12.7	14.2
b16+dye-2H	2	1088.913	1088.922	-8.2	2.22E+04	17.6	19.6
b17+dye-2H	2	1124.432	1124.445	-11.8	2.85E+04	22.6	25.2
b18+dye-2H	2	1192.961	1192.975	-11.9	1.21E+04	9.6	10.7
b19+dye-2H	2	1228.48	1228.49	-8.6	9.23E+03	7.3	8.2
b20+dye-2H	2	1263.998	1264.014	-12.4	8.50E+03	6.7	7.5
b21+dye-2H	2	1299.517	1299.524	-5.9	7.58E+03	6.0	6.7
b22+dye-2H	2	1351.021	1351.031	-7.5	7.33E+03	5.8	6.5
b23+dye-2H	2	1386.54	1386.548	-6.3	7.85E+03	6.2	6.9
b14+dye-2H	3	678.9194	678.9287	-13.6	3.82E+03	3.0	3.4
b15+dye-2H	3	702.5988	702.6105	-16.7	4.25E+03	3.4	3.8
b16+dye-2H	3	726.2778	726.2864	-11.9	8.36E+03	6.6	7.4
b17+dye-2H	3	749.9568	749.9601	-4.4	1.08E+04	8.6	9.6
b18+dye-2H	3	795.6431	795.6485	-6.8	2.36E+04	18.7	20.9
b19+dye-2H	3	819.3221	819.3304	-10.1	3.18E+04	25.2	28.1
b20+dye-2H	3	843.0011	843.0046	-4.1	3.53E+04	28.0	31.2
b21+dye-2H	3	866.6801	866.6819	-2.1	2.30E+04	18.3	20.4
b22+dye-2H	3	901.0164	901.0229	-7.2	4.02E+04	31.9	35.6
b23+dye-2H	3	924.6954	924.6991	-4.0	1.81E+04	14.4	16.0
y14+dye-2H	1	2000.795	2000.802	-3.8	2.37E+02	0.2	0.2
y13+dye-2H	1	1929.758	1929.796	-19.8	4.30E+02	0.3	0.4
y12+dye-2H	1	1858.721	1858.754	-18.1	5.05E+02	0.4	0.4
y11+dye-2H	1	1787.684	1787.697	-7.6	1.11E+03	0.9	1.0
y10+dye-2H	1	1650.625	1650.642	-10.6	1.80E+03	1.4	1.6
y9+dye-2H	1	1579.588	1579.606	-11.7	2.50E+03	2.0	2.2
y8+dye-2H	1	1508.551	1508.572	-14.3	3.23E+03	2.6	2.9
y7+dye-2H	1	1437.514	1437.533	-13.5	5.56E+03	4.4	4.9
y6+dye-2H	1	1300.455	1300.469	-10.8	1.02E+04	8.1	9.0
y5+dye-2H	1	1229.418	1229.424	-4.8	7.56E+03	6.0	6.7
y4+dye-2H	1	1158.381	1158.397	-14.2	6.06E+03	4.8	5.4
y3+dye-2H	1	1087.344	1087.367	-21.1	5.13E+03	4.1	4.5
y21+dye-2H	2	1315.553	1315.579	-19.7	1.10E+03	0.9	1.0
y20+dye-2H	2	1280.035	1280.051	-13.1	3.18E+03	2.5	2.8
y19+dye-2H	2	1211.505	1211.518	-11.0	6.46E+03	5.1	5.7
y18+dye-2H	2	1175.987	1175.997	-8.8	6.71E+03	5.3	5.9
y17+dye-2H	2	1140.468	1140.488	-17.3	5.20E+03	4.1	4.6
y16+dye-2H	2	1104.949	1104.957	-7.4	5.27E+03	4.2	4.7
y15+dye-2H	2	1036.42	1036.426	-5.8	3.08E+03	2.4	2.7
y11+dye-2H	2	894.3455	894.352	-7.2	2.86E+03	2.3	2.5
y9+dye-2H	2	790.2975	790.313	-19.6	2.52E+03	2.0	2.2
y7+dye-2H	2	719.2605	719.2677	-10.0	3.99E+03	3.2	3.5
y20+dye-2H	3	853.6921	853.6985	-7.5	4.95E+03	3.9	4.4

Supplementary Table 5. Summary of the conformer 1 CID fragment ions from the TWIM-CID experiments. Precursor ion is the $[M+3H]^{3+}$ ions. 44 eV was used in the transfer region of the Triwave.

Assignment	Charge state	Theo. m/z	Expt. m/z	Error (ppm)	Int.	Rel. Int. (%)	Norm. to $[M+3H]^{3+}$ (%)
M+3H	3	973.4053	973.4084	-3.2	5.92E+03	66.4	100.0
b2	1	217.065	217.066	-4.6	4.14E+02	4.6	7.0
b3	1	288.102	288.1044	-8.3	6.35E+02	7.1	10.7
b4	1	359.139	359.1352	10.6	1.84E+02	2.1	3.1
b5	1	496.198	496.2007	-5.4	5.76E+02	6.5	9.7
b6	1	567.235	567.2331	3.3	5.36E+02	6.0	9.1
b7	1	638.272	638.2639	12.7	3.29E+02	3.7	5.6
b8	1	709.309	709.3035	7.8	3.13E+02	3.5	5.3
b9	1	846.368	846.3798	-13.9	2.68E+02	3.0	4.5
b13	1	1130.516	1130.514	2.0	2.03E+02	0.2	0.2
b10	2	459.206	459.2137	-16.8	6.20E+01	0.7	1.0
b11	2	494.725	494.7236	2.8	3.02E+02	3.4	5.1
b12	2	530.243	530.2382	9.1	3.47E+02	3.9	5.9
b13	2	565.762	565.7576	7.8	2.09E+02	2.3	3.5
b14	2	634.291	634.2885	3.9	5.22E+02	5.9	8.8
b15	2	669.81	669.8024	11.3	4.00E+02	4.5	6.8
b16	2	705.328	705.3326	-6.5	5.50E+01	0.6	0.9
b17	2	740.847	740.8419	6.9	3.84E+02	4.3	6.5
b18	2	809.376	809.3762	-0.2	3.59E+02	4.0	6.1
b19	2	844.895	844.8943	0.8	3.38E+02	3.8	5.7
b20	2	880.414	880.4107	3.7	3.50E+02	3.9	5.9
b21	2	915.932	915.9315	0.5	3.24E+02	3.6	5.5
y18	1	1583.797	1583.791	4.0	7.10E+01	0.8	1.2
y17	1	1512.76	1512.78	-13.5	2.02E+02	2.3	3.4
y16	1	1441.722	1441.741	-12.9	3.11E+02	3.5	5.3
y15	1	1304.663	1304.676	-9.7	8.26E+02	9.3	14.0
y14	1	1233.626	1233.652	-21.2	1.26E+03	14.1	21.3
y13	1	1162.589	1162.591	-1.9	9.41E+02	10.6	15.9
y12	1	1091.552	1091.56	-7.2	1.02E+03	11.4	17.2
y11	1	1020.515	1020.521	-5.7	1.82E+03	20.4	30.7
y10	1	883.456	883.4575	-1.7	1.24E+03	13.9	20.9
y9	1	812.419	812.422	-3.7	1.07E+03	12.0	18.1
y8	1	741.382	741.3648	23.2	8.46E+02	9.5	14.3
y7	1	670.345	670.3561	-16.6	1.10E+03	12.3	18.6
y6	1	533.286	533.2886	-4.9	1.32E+03	14.8	22.3
y5	1	462.249	462.2499	-1.9	1.65E+03	18.5	27.9
y4	1	391.212	391.2103	4.3	1.22E+03	13.7	20.6
y3	1	320.175	320.177	-6.2	1.03E+03	11.6	17.4

y2	1	217.165	217.1625	11.5	1.36E+03	15.3	23.0
y24	2	1075.516	1075.537	-19.2	1.51E+02	1.7	2.6
y23	2	1003.006	1003.01	-4.0	2.95E+03	33.1	49.8
y22	2	967.487	967.4788	8.5	1.46E+03	16.4	24.7
y21	2	931.969	931.9617	7.8	1.98E+03	22.2	33.4
y20	2	896.45	896.4552	-5.8	2.42E+03	27.2	40.9
y19	2	827.921	827.9127	10.0	1.04E+03	11.7	17.6
y18	2	792.402	792.4008	1.5	8.91E+02	10.0	15.1
y17	2	756.883	756.8863	-4.4	6.35E+02	7.1	10.7
y16	2	721.365	721.3651	-0.1	1.30E+03	14.6	22.0
y15	2	652.835	652.8389	-6.0	7.64E+02	8.6	12.9
y14	2	617.317	617.3064	17.2	6.15E+02	6.9	10.4
y13	2	581.798	581.799	-1.7	2.93E+02	3.3	4.9
y12	2	546.28	546.2712	16.1	3.05E+02	3.4	5.2
y11	2	510.761	510.7571	7.6	9.06E+02	10.2	15.3
b2+dye-2H	1	984.2337	984.2533	-19.9	1.24E+03	13.9	20.9
b3+dye-2H	1	1055.271	1055.288	-16.1	4.96E+02	5.6	8.4
b5+dye-2H	1	1263.367	1263.393	-20.7	1.09E+03	12.2	18.4
b6+dye-2H	1	1334.404	1334.442	-28.7	9.10E+02	10.2	15.4
b7+dye-2H	1	1405.441	1405.453	-9.0	9.95E+02	11.2	16.8
b8+dye-2H	1	1476.478	1476.493	-10.5	1.40E+03	15.7	23.6
b9+dye-2H	1	1613.537	1613.57	-20.4	2.20E+02	2.5	3.7
b10+dye-2H	1	1684.574	1684.586	-7.5	1.90E+02	2.1	3.2
b11+dye-2H	1	1755.611	1755.632	-12.2	7.30E+01	0.8	1.2
b12+dye-2H	1	1826.648	1826.671	-12.6	8.00E+01	0.9	1.4
b13+dye-2H	1	1897.685	1897.73	-23.7	1.40E+02	1.6	2.4
b14+dye-2H	1	2034.744	2034.768	-11.7	2.60E+01	0.3	0.4
b15+dye-2H	1	2105.782	2105.819	-17.7	1.20E+01	0.1	0.2
b16+dye-2H	1	2176.819	2176.827	-3.7	1.70E+01	0.2	0.3
b17+dye-2H	1	2247.856	2247.853	1.4	1.60E+01	0.2	0.3
b5+dye-2H	2	632.187	632.2011	-22.3	1.24E+03	13.9	20.9
b6+dye-2H	2	667.7055	667.7251	-29.3	1.00E+03	11.2	16.9
b10+dye-2H	2	842.7905	842.8018	-13.4	2.97E+03	33.3	50.2
b11+dye-2H	2	878.309	878.3264	-19.8	3.50E+03	39.3	59.1
b12+dye-2H	2	913.8275	913.8452	-19.3	3.12E+03	35.0	52.7
b13+dye-2H	2	949.346	949.3547	-9.1	1.86E+03	20.9	31.4
b14+dye-2H	2	1017.876	1017.887	-10.9	1.42E+03	15.9	24.0
b15+dye-2H	2	1053.395	1053.402	-7.4	9.98E+02	11.2	16.9
b16+dye-2H	2	1088.913	1088.908	5.0	1.49E+03	16.7	25.2
b17+dye-2H	2	1124.432	1124.445	-11.8	1.76E+03	19.8	29.7
b18+dye-2H	2	1192.961	1192.975	-11.9	5.90E+02	6.6	10.0
b19+dye-2H	2	1228.48	1228.49	-8.6	6.84E+02	7.7	11.6
b20+dye-2H	2	1263.998	1264.014	-12.4	5.98E+02	6.7	10.1
b21+dye-2H	2	1299.517	1299.54	-18.0	5.94E+02	6.7	10.0

b22+dye-2H	2	1351.021	1351.015	4.5	5.09E+02	5.7	8.6
b23+dye-2H	2	1386.54	1386.548	-6.3	4.72E+02	5.3	8.0
b14+dye-2H	3	678.9194	678.9287	-13.6	4.64E+02	5.2	7.8
b15+dye-2H	3	702.5988	702.6105	-16.7	3.08E+02	3.5	5.2
b16+dye-2H	3	726.2778	726.2864	-11.9	4.68E+02	5.3	7.9
b17+dye-2H	3	749.9568	749.9601	-4.4	6.26E+02	7.0	10.6
b18+dye-2H	3	795.6431	795.6485	-6.8	1.71E+03	19.2	28.9
b19+dye-2H	3	819.3221	819.3304	-10.1	2.35E+03	26.4	39.7
b20+dye-2H	3	843.0011	843.0046	-4.1	2.50E+03	28.1	42.2
b21+dye-2H	3	866.6801	866.6947	-16.8	1.43E+03	16.0	24.2
b22+dye-2H	3	901.0164	901.0229	-7.2	2.24E+03	25.1	37.8
b23+dye-2H	3	924.6954	924.6991	-4.0	9.47E+02	10.6	16.0
y14+dye-2H	1	2000.795	2000.802	-3.8	9.00E+00	0.1	0.2
y13+dye-2H	1	1929.758	1929.796	-19.8	1.70E+01	0.2	0.3
y12+dye-2H	1	1858.721	1858.754	-18.1	2.20E+01	0.2	0.4
y11+dye-2H	1	1787.684	1787.716	-18.0	5.00E+01	0.6	0.8
y10+dye-2H	1	1650.625	1650.678	-32.1	7.40E+01	0.8	1.3
y9+dye-2H	1	1579.588	1579.606	-11.7	1.52E+02	1.7	2.6
y8+dye-2H	1	1508.551	1508.572	-14.3	1.29E+02	1.4	2.2
y7+dye-2H	1	1437.514	1437.533	-13.5	3.91E+02	4.4	6.6
y6+dye-2H	1	1300.455	1300.485	-22.9	7.24E+02	8.1	12.2
y4+dye-2H	1	1158.381	1158.397	-14.2	4.97E+02	5.6	8.4
y3+dye-2H	1	1087.344	1087.381	-34.4	3.49E+02	3.9	5.9
y21+dye-2H	2	1315.553	1315.579	-19.7	1.13E+02	1.3	1.9
y20+dye-2H	2	1280.035	1280.036	-0.8	7.70E+01	0.9	1.3
y19+dye-2H	2	1211.505	1211.518	-11.0	3.16E+02	3.5	5.3
y18+dye-2H	2	1175.987	1175.997	-8.8	5.19E+02	5.8	8.8
y17+dye-2H	2	1140.468	1140.488	-17.3	2.19E+02	2.5	3.7
y16+dye-2H	2	1104.949	1104.972	-20.4	2.36E+02	2.6	4.0
y15+dye-2H	2	1036.42	1036.426	-5.8	2.86E+02	3.2	4.8
y11+dye-2H	2	894.3455	894.3651	-21.9	2.63E+02	3.0	4.4
y9+dye-2H	2	790.2975	790.3007	-4.0	1.61E+02	1.8	2.7
y7+dye-2H	2	719.2605	719.2677	-10.0	3.67E+02	4.1	6.2
y20+dye-2H	3	853.6921	853.6985	-7.5	4.62E+02	5.2	7.8

Supplementary Table 6. Summary of the conformer 2 CID fragment ions from the TWIM-CID experiments. Precursor ion is the $[M+3H]^{3+}$ ions. 44 eV was used in the transfer region of the Triwave.

Assignment	Charge state	Theo. m/z	Expt. m/z	Error (ppm)	Int.	Rel. Int. (%)
M+3H	3	973.4053	973.4084	-3.2	9.10E+03	100.0
b2	1	217.065	217.0593	26.3	1.97E+03	21.6
b3	1	288.102	288.097	17.4	4.51E+03	49.6
b4	1	359.139	359.1352	10.6	1.74E+03	19.1
b5	1	496.198	496.191	14.1	6.22E+03	68.4
b6	1	567.235	567.2331	3.3	5.25E+03	57.7
b7	1	638.272	638.275	-4.7	3.29E+03	36.2
b8	1	709.309	709.3135	-6.3	2.78E+03	30.5
b9	1	846.368	846.3671	1.1	1.66E+03	18.2
b11	1	988.442	988.4489	-7.0	1.35E+03	14.8
b12	1	1059.479	1059.476	3.2	1.26E+03	13.8
b13	1	1130.516	1130.514	2.0	2.43E+03	26.7
b14	1	1267.575	1267.586	-8.8	9.40E+02	10.3
b15	1	1338.613	1338.624	-8.0	4.18E+02	4.6
b16	1	1409.65	1409.63	14.2	5.33E+02	5.9
b17	1	1480.687	1480.69	-2.2	8.82E+02	9.7
b9	2	423.688	423.6861	4.5	5.08E+02	5.6
b10	2	459.206	459.2044	3.5	7.57E+02	8.3
b11	2	494.725	494.7236	2.8	1.04E+03	11.4
b12	2	530.243	530.2382	9.1	1.65E+03	18.1
b13	2	565.762	565.7576	7.8	1.61E+03	17.7
b14	2	634.291	634.2885	3.9	3.50E+03	38.5
b15	2	669.81	669.8137	-5.5	3.43E+03	37.7
b16	2	705.328	705.321	9.9	4.19E+03	46.0
b17	2	740.847	740.8419	6.9	4.03E+03	44.3
b18	2	809.376	809.3762	-0.2	2.68E+03	29.5
b19	2	844.895	844.8943	0.8	2.75E+03	30.2
b20	2	880.414	880.4107	3.7	2.10E+03	23.1
b21	2	915.932	915.9315	0.5	1.52E+03	16.7
y18	1	1583.797	1583.791	4.0	2.00E+02	2.2
y17	1	1512.76	1512.78	-13.5	2.54E+02	2.8
y16	1	1441.722	1441.724	-1.4	3.03E+02	3.3
y15	1	1304.663	1304.66	2.4	6.90E+02	7.6
y14	1	1233.626	1233.622	3.6	1.04E+03	11.4
y13	1	1162.589	1162.591	-1.9	6.59E+02	7.2
y12	1	1091.552	1091.545	6.0	6.99E+02	7.7
y11	1	1020.515	1020.507	8.0	6.95E+02	7.6
y10	1	883.456	883.4575	-1.7	7.40E+02	8.1
y9	1	812.419	812.422	-3.7	4.78E+02	5.3

y6	1	533.286	533.2886	-4.9	5.62E+02	6.2
y5	1	462.249	462.2499	-1.9	6.01E+02	6.6
y4	1	391.212	391.2103	4.3	3.06E+02	3.4
y3	1	320.175	320.177	-6.2	4.60E+02	5.1
y2	1	217.165	217.1625	11.5	6.68E+02	7.3
y23	2	1003.006	1003.01	-4.0	1.05E+03	11.5
y22	2	967.487	967.4788	8.5	4.87E+02	5.4
y21	2	931.969	931.975	-6.4	7.64E+02	8.4
y20	2	896.45	896.4421	8.8	6.94E+02	7.6
y19	2	827.921	827.9127	10.0	3.77E+02	4.1
y18	2	792.402	792.4008	1.5	2.62E+02	2.9
y17	2	756.883	756.8863	-4.4	2.21E+02	2.4
y16	2	721.365	721.3651	-0.1	3.97E+02	4.4
y15	2	652.835	652.8389	-6.0	4.55E+02	5.0
y14	2	617.317	617.3281	-18.0	4.13E+02	4.5
y13	2	581.798	581.799	-1.7	1.24E+02	1.4
y12	2	546.28	546.2712	16.1	1.12E+02	1.2
y11	2	510.761	510.7571	7.6	2.77E+02	3.0
b2+dye-2H	1	984.2337	984.2533	-19.9	5.01E+02	5.5
b3+dye-2H	1	1055.271	1055.302	-29.5	2.70E+02	3.0
b5+dye-2H	1	1263.367	1263.408	-33.0	5.30E+02	5.8
b6+dye-2H	1	1334.404	1334.426	-16.7	5.56E+02	6.1
b7+dye-2H	1	1405.441	1405.47	-20.6	5.97E+02	6.6
b8+dye-2H	1	1476.478	1476.493	-10.5	8.87E+02	9.7
b9+dye-2H	1	1613.537	1613.552	-9.5	1.73E+02	1.9
b10+dye-2H	1	1684.574	1684.586	-7.5	1.34E+02	1.5
b11+dye-2H	1	1755.611	1755.632	-12.2	1.80E+02	2.0
b12+dye-2H	1	1826.648	1826.671	-12.6	1.64E+02	1.8
b13+dye-2H	1	1897.685	1897.73	-23.7	2.12E+02	2.3
b14+dye-2H	1	2034.744	2034.768	-11.7	4.00E+01	0.4
b15+dye-2H	1	2105.782	2105.799	-8.2	1.80E+01	0.2
b17+dye-2H	1	2247.856	2247.894	-17.0	1.20E+01	0.1
b5+dye-2H	2	632.187	632.2122	-39.8	5.32E+02	5.8
b6+dye-2H	2	667.7055	667.7251	-29.3	2.97E+02	3.3
b10+dye-2H	2	842.7905	842.8018	-13.4	7.86E+02	8.6
b11+dye-2H	2	878.309	878.3135	-5.1	8.89E+02	9.8
b12+dye-2H	2	913.8275	913.8452	-19.3	8.32E+02	9.1
b13+dye-2H	2	949.346	949.3682	-23.4	7.60E+02	8.4
b14+dye-2H	2	1017.876	1017.873	2.8	3.28E+02	3.6
b15+dye-2H	2	1053.395	1053.417	-21.0	2.33E+02	2.6
b16+dye-2H	2	1088.913	1088.922	-8.2	8.64E+02	9.5
b17+dye-2H	2	1124.432	1124.445	-11.8	6.79E+02	7.5
b19+dye-2H	2	1228.48	1228.49	-8.6	2.25E+02	2.5
b20+dye-2H	2	1263.998	1263.998	-0.1	2.69E+02	3.0

b21+dye-2H	2	1299.517	1299.524	-5.9	2.00E+02	2.2
b22+dye-2H	2	1351.021	1351.031	-7.5	2.06E+02	2.3
b23+dye-2H	2	1386.54	1386.548	-6.3	2.79E+02	3.1
b14+dye-2H	3	678.9194	678.9287	-13.6	1.30E+02	1.4
b15+dye-2H	3	702.5988	702.6105	-16.7	2.31E+02	2.5
b16+dye-2H	3	726.2778	726.2982	-28.1	2.77E+02	3.0
b17+dye-2H	3	749.9568	749.972	-20.3	2.06E+02	2.3
b18+dye-2H	3	795.6431	795.6485	-6.8	8.83E+02	9.7
b19+dye-2H	3	819.3221	819.3304	-10.1	6.59E+02	7.2
b20+dye-2H	3	843.0011	843.0173	-19.2	6.59E+02	7.2
b21+dye-2H	3	866.6801	866.6947	-16.8	6.71E+02	7.4
b22+dye-2H	3	901.0164	901.0229	-7.2	1.24E+03	13.6
b23+dye-2H	3	924.6954	924.6991	-4.0	5.80E+02	6.4
y14+dye-2H	1	2000.795	2000.802	-3.8	9.50E+01	1.0
y13+dye-2H	1	1929.758	1929.796	-19.8	1.16E+02	1.3
y12+dye-2H	1	1858.721	1858.736	-7.9	1.57E+02	1.7
y11+dye-2H	1	1787.684	1787.697	-7.6	2.98E+02	3.3
y10+dye-2H	1	1650.625	1650.625	0.1	5.90E+02	6.5
y9+dye-2H	1	1579.588	1579.606	-11.7	8.87E+02	9.7
y8+dye-2H	1	1508.551	1508.589	-25.5	9.95E+02	10.9
y7+dye-2H	1	1437.514	1437.533	-13.5	1.81E+03	19.9
y6+dye-2H	1	1300.455	1300.469	-10.8	2.02E+03	22.2
y5+dye-2H	1	1229.418	1229.424	-4.8	2.17E+03	23.8
y4+dye-2H	1	1158.381	1158.397	-14.2	1.66E+03	18.2
y3+dye-2H	1	1087.344	1087.367	-21.1	1.41E+03	15.5
y21+dye-2H	2	1315.553	1315.563	-7.7	2.84E+02	3.1
y20+dye-2H	2	1280.035	1280.051	-13.1	1.02E+03	11.2
y19+dye-2H	2	1211.505	1211.518	-11.0	1.96E+03	21.5
y18+dye-2H	2	1175.987	1175.997	-8.8	1.43E+03	15.7
y17+dye-2H	2	1140.468	1140.488	-17.3	1.44E+03	15.8
y16+dye-2H	2	1104.949	1104.957	-7.4	1.60E+03	17.6
y15+dye-2H	2	1036.42	1036.426	-5.8	7.59E+02	8.3
y11+dye-2H	2	894.3455	894.3651	-21.9	9.24E+02	10.2
y9+dye-2H	2	790.2975	790.3007	-4.0	4.40E+02	4.8
y7+dye-2H	2	719.2605	719.2794	-26.2	1.15E+03	12.6
y20+dye-2H	3	853.6921	853.6985	-7.5	1.13E+03	12.4

Supplementary Table 7. Summary of the conformer 3 CID fragment ions from the TWIM-CID experiments. Precursor ion is the $[M+3H]^{3+}$ ions. 44 eV was used in the transfer region of the Triwave.

Assignment	Charge state	Theo. m/z	Expt. m/z	Error (ppm)	Int.	Rel. Int. (%)
M+3H	3	973.4053	973.4084	-3.2	3.48E+03	100.0
b2	1	217.065	217.0596	24.9	5.59E+02	16.1
b3	1	288.102	288.097	17.4	2.05E+03	58.9
b4	1	359.139	359.1352	10.6	9.46E+02	27.2
b5	1	496.198	496.2007	-5.4	2.18E+03	62.6
b6	1	567.235	567.2331	3.3	1.64E+03	47.1
b7	1	638.272	638.275	-4.7	1.19E+03	34.2
b8	1	709.309	709.3135	-6.3	9.53E+02	27.4
b9	1	846.368	846.3798	-13.9	4.74E+02	13.6
b11	1	988.442	988.4489	-7.0	3.53E+02	10.1
b12	1	1059.479	1059.49	-10.1	5.34E+02	15.3
b13	1	1130.516	1130.514	2.0	8.12E+02	23.3
b14	1	1267.575	1267.586	-8.8	3.55E+02	10.2
b15	1	1338.613	1338.624	-8.0	1.94E+02	5.6
b16	1	1409.65	1409.663	-9.0	1.81E+02	5.2
b17	1	1480.687	1480.69	-2.2	3.30E+02	9.5
b9	2	423.688	423.6861	4.5	2.45E+02	7.0
b10	2	459.206	459.195	24.0	2.96E+02	8.5
b11	2	494.725	494.7236	2.8	3.86E+02	11.1
b12	2	530.243	530.2482	-9.8	7.20E+02	20.7
b13	2	565.762	565.7576	7.8	6.31E+02	18.1
b14	2	634.291	634.2885	3.9	1.20E+03	34.5
b15	2	669.81	669.8137	-5.5	1.64E+03	47.1
b16	2	705.328	705.321	9.9	1.64E+03	47.1
b17	2	740.847	740.8419	6.9	1.60E+03	46.0
b18	2	809.376	809.3762	-0.2	1.19E+03	34.2
b19	2	844.895	844.8943	0.8	8.73E+02	25.1
b20	2	880.414	880.4107	3.7	5.59E+02	16.1
b21	2	915.932	915.9315	0.5	1.08E+03	31.0
y18	1	1583.797	1583.791	4.0	1.49E+02	4.3
y17	1	1512.76	1512.747	8.9	1.22E+02	3.5
y16	1	1441.722	1441.724	-1.4	1.53E+02	4.4
y15	1	1304.663	1304.691	-21.8	1.49E+02	4.3
y14	1	1233.626	1233.622	3.6	1.44E+02	4.1
y13	1	1162.589	1162.591	-1.9	3.26E+02	9.4
y12	1	1091.552	1091.56	-7.2	1.54E+02	4.4
y11	1	1020.515	1020.535	-19.3	1.93E+02	5.5
y10	1	883.456	883.4575	-1.7	2.22E+02	6.4
y9	1	812.419	812.4344	-19.0	1.51E+02	4.3

y6	1	533.286	533.2886	-4.9	1.14E+02	3.3
y5	1	462.249	462.2499	-1.9	1.35E+02	3.9
y4	1	391.212	391.2017	26.3	1.80E+02	5.2
y3	1	320.175	320.1692	18.1	1.29E+02	3.7
y2	1	217.165	217.1625	11.5	1.84E+02	5.3
y23	2	1003.006	1003.01	-4.0	2.88E+02	8.3
y22	2	967.487	967.4788	8.5	3.10E+02	8.9
y21	2	931.969	931.975	-6.4	1.39E+02	4.0
y20	2	896.45	896.4552	-5.8	2.75E+02	7.9
y19	2	827.921	827.9127	10.0	1.67E+02	4.8
y18	2	792.402	792.4008	1.5	7.30E+01	2.1
y17	2	756.883	756.8863	-4.4	9.70E+01	2.8
y16	2	721.365	721.3651	-0.1	1.28E+02	3.7
y15	2	652.835	652.8389	-6.0	6.50E+01	1.9
y14	2	617.317	617.3173	-0.5	5.50E+01	1.6
y12	2	546.28	546.2916	-21.2	6.50E+01	1.9
y11	2	510.761	510.748	25.5	6.20E+01	1.8
b2+dye-2H	1	984.2337	984.2533	-19.9	1.84E+02	5.3
b3+dye-2H	1	1055.271	1055.316	-42.9	1.54E+02	4.4
b5+dye-2H	1	1263.367	1263.377	-8.4	1.12E+02	3.2
b6+dye-2H	1	1334.404	1334.442	-28.7	1.33E+02	3.8
b7+dye-2H	1	1405.441	1405.486	-32.3	1.81E+02	5.2
b8+dye-2H	1	1476.478	1476.51	-21.8	3.30E+02	9.5
b9+dye-2H	1	1613.537	1613.57	-20.4	4.10E+01	1.2
b10+dye-2H	1	1684.574	1684.586	-7.5	2.30E+01	0.7
b11+dye-2H	1	1755.611	1755.65	-22.6	3.80E+01	1.1
b12+dye-2H	1	1826.648	1826.69	-22.9	3.30E+01	0.9
b13+dye-2H	1	1897.685	1897.73	-23.7	5.20E+01	1.5
b14+dye-2H	1	2034.744	2034.768	-11.7	2.90E+01	0.8
b15+dye-2H	1	2105.782	2105.799	-8.2	3.50E+01	1.0
b16+dye-2H	1	2176.819	2176.827	-3.7	2.20E+01	0.6
b5+dye-2H	2	632.187	632.2122	-39.8	1.97E+02	5.7
b6+dye-2H	2	667.7055	667.7251	-29.3	7.50E+01	2.2
b10+dye-2H	2	842.7905	842.8018	-13.4	3.19E+02	9.2
b11+dye-2H	2	878.309	878.3135	-5.1	2.29E+02	6.6
b12+dye-2H	2	913.8275	913.8318	-4.7	2.33E+02	6.7
b13+dye-2H	2	949.346	949.3547	-9.1	2.67E+02	7.7
b14+dye-2H	2	1017.876	1017.873	2.8	1.32E+02	3.8
b16+dye-2H	2	1088.913	1088.951	-34.6	2.83E+02	8.1
b17+dye-2H	2	1124.432	1124.445	-11.8	1.63E+02	4.7
b19+dye-2H	2	1228.48	1228.49	-8.6	1.22E+02	3.5
b20+dye-2H	2	1263.998	1264.029	-24.7	5.40E+01	1.6
b22+dye-2H	2	1351.021	1351.047	-19.4	7.60E+01	2.2
b14+dye-2H	3	678.9194	678.9174	3.0	8.20E+01	2.4

b16+dye-2H	3	726.2778	726.2746	4.4	9.10E+01	2.6
b17+dye-2H	3	749.9568	749.972	-20.3	6.40E+01	1.8
b18+dye-2H	3	795.6431	795.6485	-6.8	1.99E+02	5.7
b19+dye-2H	3	819.3221	819.3304	-10.1	2.21E+02	6.4
b20+dye-2H	3	843.0011	843.0046	-4.1	2.20E+02	6.3
b21+dye-2H	3	866.6801	866.6947	-16.8	2.37E+02	6.8
b22+dye-2H	3	901.0164	901.0229	-7.2	4.73E+02	13.6
b23+dye-2H	3	924.6954	924.6991	-4.0	3.18E+02	9.1
y14+dye-2H	1	2000.795	2000.802	-3.8	4.10E+01	1.2
y13+dye-2H	1	1929.758	1929.796	-19.8	9.00E+01	2.6
y12+dye-2H	1	1858.721	1858.736	-7.9	1.14E+02	3.3
y11+dye-2H	1	1787.684	1787.697	-7.6	1.41E+02	4.1
y10+dye-2H	1	1650.625	1650.642	-10.6	2.32E+02	6.7
y9+dye-2H	1	1579.588	1579.641	-33.8	3.32E+02	9.5
y8+dye-2H	1	1508.551	1508.589	-25.5	3.64E+02	10.5
y7+dye-2H	1	1437.514	1437.533	-13.5	6.26E+02	18.0
y6+dye-2H	1	1300.455	1300.485	-22.9	7.48E+02	21.5
y5+dye-2H	1	1229.418	1229.424	-4.8	9.06E+02	26.0
y4+dye-2H	1	1158.381	1158.412	-27.0	7.27E+02	20.9
y3+dye-2H	1	1087.344	1087.367	-21.1	4.71E+02	13.5
y21+dye-2H	2	1315.553	1315.563	-7.7	1.53E+02	4.4
y20+dye-2H	2	1280.035	1280.051	-13.1	3.32E+02	9.5
y19+dye-2H	2	1211.505	1211.518	-11.0	7.48E+02	21.5
y18+dye-2H	2	1175.987	1175.997	-8.8	6.16E+02	17.7
y17+dye-2H	2	1140.468	1140.473	-4.5	4.57E+02	13.1
y16+dye-2H	2	1104.949	1104.957	-7.4	5.54E+02	15.9
y15+dye-2H	2	1036.42	1036.426	-5.8	3.15E+02	9.1
y11+dye-2H	2	894.3455	894.3651	-21.9	3.83E+02	11.0
y9+dye-2H	2	790.2975	790.313	-19.6	2.70E+02	7.8
y7+dye-2H	2	719.2605	719.2677	-10.0	4.90E+02	14.1
y20+dye-2H	3	853.6921	853.6985	-7.5	3.74E+02	10.7

Supplementary Table 8. Summary of the conformer 4 CID fragment ions from the TWIM-CID experiments. Precursor ion is the $[M+3H]^{3+}$ ions. 44 eV was used in the transfer region of the Triwave.

Assignment	Charge state	Theo. m/z	Expt. m/z	Error (ppm)	Int.	Rel. Int. (%)
M+3H	3	973.4053	973.4084	-3.2	6.37E+04	71.3
b2	1	217.065	217.0596	24.9	2.19E+02	0.2
b3	1	288.102	288.097	17.4	1.44E+03	1.6
b4	1	359.139	359.1352	10.6	1.52E+02	0.2
b5	1	496.198	496.2007	-5.4	1.36E+03	1.5
b6	1	567.235	567.2331	3.3	3.86E+02	0.4
b7	1	638.272	638.275	-4.7	3.57E+02	0.4
b8	1	709.309	709.2918	24.2	2.63E+02	0.3
b11	1	988.442	988.4489	-7.0	3.26E+03	3.7
b13	1	1130.516	1130.514	2.0	4.65E+02	0.5
b10	2	459.206	459.2044	3.5	1.94E+02	0.2
b12	2	530.243	530.2482	-9.8	1.67E+02	0.2
b13	2	565.762	565.7679	-10.4	1.54E+02	0.2
b14	2	634.291	634.2885	3.9	1.04E+03	1.2
b16	2	705.328	705.3442	-23.0	5.86E+02	0.7
b21	2	915.932	915.9315	0.5	6.37E+02	0.7
y19	1	1654.834	1654.867	-19.6	1.15E+03	1.3
y18	1	1583.797	1583.791	4.0	2.06E+03	2.3
y17	1	1512.76	1512.763	-2.2	3.01E+03	3.4
y16	1	1441.722	1441.724	-1.4	5.35E+03	6.0
y15	1	1304.663	1304.66	2.4	1.28E+04	14.3
y14	1	1233.626	1233.622	3.6	1.78E+04	19.9
y13	1	1162.589	1162.591	-1.9	1.39E+04	15.6
y12	1	1091.552	1091.56	-7.2	1.41E+04	15.8
y11	1	1020.515	1020.521	-5.7	2.06E+04	23.1
y10	1	883.456	883.4575	-1.7	1.92E+04	21.5
y9	1	812.419	812.422	-3.7	1.38E+04	15.5
y8	1	741.382	741.3766	7.3	8.78E+03	9.8
y7	1	670.345	670.3448	0.3	1.47E+04	16.5
y6	1	533.286	533.2886	-4.9	1.45E+04	16.2
y5	1	462.249	462.2499	-1.9	1.42E+04	15.9
y4	1	391.212	391.2103	4.3	1.19E+04	13.3
y3	1	320.175	320.1692	18.1	1.10E+04	12.3
y2	1	217.165	217.1625	11.5	8.75E+03	9.8
y24	2	1075.516	1075.537	-19.2	3.14E+03	3.5
y23	2	1003.006	1003.01	-4.0	3.27E+04	36.6
y22	2	967.487	967.4924	-5.6	1.67E+04	18.7
y21	2	931.969	931.9617	7.8	1.86E+04	20.8
y20	2	896.45	896.4421	8.8	1.95E+04	21.8

y19	2	827.921	827.9253	-5.2	1.12E+04	12.5
y18	2	792.402	792.4008	1.5	7.66E+03	8.6
y17	2	756.883	756.8743	11.5	6.87E+03	7.7
y16	2	721.365	721.3651	-0.1	1.38E+04	15.5
y15	2	652.835	652.8389	-6.0	6.30E+03	7.1
y14	2	617.317	617.3173	-0.5	4.52E+03	5.1
y13	2	581.798	581.799	-1.7	2.23E+03	2.5
y12	2	546.28	546.2814	-2.6	3.11E+03	3.5
y11	2	510.761	510.7571	7.6	7.15E+03	8.0
b2+dye-2H	1	984.2337	984.2533	-19.9	1.44E+04	16.1
b3+dye-2H	1	1055.271	1055.302	-29.5	6.12E+03	6.9
b5+dye-2H	1	1263.367	1263.393	-20.7	1.28E+04	14.3
b6+dye-2H	1	1334.404	1334.426	-16.7	1.07E+04	12.0
b7+dye-2H	1	1405.441	1405.47	-20.6	1.32E+04	14.8
b8+dye-2H	1	1476.478	1476.493	-10.5	2.11E+04	23.6
b9+dye-2H	1	1613.537	1613.57	-20.4	3.72E+03	4.2
b10+dye-2H	1	1684.574	1684.586	-7.5	2.08E+03	2.3
b11+dye-2H	1	1755.611	1755.65	-22.6	1.81E+03	2.0
b12+dye-2H	1	1826.648	1826.671	-12.6	1.59E+03	1.8
b13+dye-2H	1	1897.685	1897.711	-13.7	3.29E+02	0.4
b14+dye-2H	1	2034.744	2034.768	-11.7	2.43E+02	0.3
b16+dye-2H	1	2176.819	2176.827	-3.7	1.28E+02	0.1
b17+dye-2H	1	2247.856	2247.853	1.4	1.14E+02	0.1
b5+dye-2H	2	632.187	632.2011	-22.3	1.28E+04	14.3
b6+dye-2H	2	667.7055	667.7138	-12.4	8.02E+03	9.0
b10+dye-2H	2	842.7905	842.8018	-13.4	2.97E+04	33.3
b11+dye-2H	2	878.309	878.3135	-5.1	2.78E+04	31.1
b12+dye-2H	2	913.8275	913.8452	-19.3	2.36E+04	26.4
b13+dye-2H	2	949.346	949.3547	-9.1	1.71E+04	19.1
b14+dye-2H	2	1017.876	1017.887	-10.9	1.19E+04	13.3
b15+dye-2H	2	1053.395	1053.417	-21.0	9.10E+03	10.2
b16+dye-2H	2	1088.913	1088.922	-8.2	1.50E+04	16.8
b17+dye-2H	2	1124.432	1124.445	-11.8	2.04E+04	22.8
b18+dye-2H	2	1192.961	1192.975	-11.9	8.95E+03	10.0
b19+dye-2H	2	1228.48	1228.49	-8.6	6.32E+03	7.1
b20+dye-2H	2	1263.998	1264.014	-12.4	6.29E+03	7.0
b21+dye-2H	2	1299.517	1299.524	-5.9	5.55E+03	6.2
b22+dye-2H	2	1351.021	1351.031	-7.5	5.31E+03	5.9
b23+dye-2H	2	1386.54	1386.548	-6.3	5.58E+03	6.2
b14+dye-2H	3	678.9194	678.9287	-13.6	2.53E+03	2.8
b15+dye-2H	3	702.5988	702.6105	-16.7	3.32E+03	3.7
b16+dye-2H	3	726.2778	726.2864	-11.9	5.49E+03	6.1
b17+dye-2H	3	749.9568	749.9601	-4.4	6.23E+03	7.0
b18+dye-2H	3	795.6431	795.6485	-6.8	1.59E+04	17.8

b19+dye-2H	3	819.3221	819.3304	-10.1	2.23E+04	25.0
b20+dye-2H	3	843.0011	843.0046	-4.1	2.51E+04	28.1
b21+dye-2H	3	866.6801	866.6819	-2.1	1.67E+04	18.7
b22+dye-2H	3	901.0164	901.0229	-7.2	1.98E+04	22.2
b23+dye-2H	3	924.6954	924.6991	-4.0	1.20E+04	13.4
y13+dye-2H	1	1929.758	1929.796	-19.8	5.10E+01	0.1
y12+dye-2H	1	1858.721	1858.717	2.2	7.40E+01	0.1
y11+dye-2H	1	1787.684	1787.697	-7.6	1.11E+02	0.1
y10+dye-2H	1	1650.625	1650.66	-21.4	1.48E+02	0.2
y9+dye-2H	1	1579.588	1579.606	-11.7	1.75E+02	0.2
y8+dye-2H	1	1508.551	1508.572	-14.3	2.78E+02	0.3
y7+dye-2H	1	1437.514	1437.533	-13.5	1.35E+03	1.5
y4+dye-2H	1	1158.381	1158.412	-27.0	1.82E+02	0.2
y3+dye-2H	1	1087.344	1087.381	-34.4	1.86E+02	0.2
y20+dye-2H	2	1280.035	1280.051	-13.1	1.73E+02	0.2
y19+dye-2H	2	1211.505	1211.518	-11.0	3.34E+02	0.4
y18+dye-2H	2	1175.987	1175.997	-8.8	2.30E+02	0.3
y17+dye-2H	2	1140.468	1140.488	-17.3	8.88E+02	1.0
y16+dye-2H	2	1104.949	1104.957	-7.4	2.37E+02	0.3
y15+dye-2H	2	1036.42	1036.454	-32.9	3.07E+02	0.3
y9+dye-2H	2	790.2975	790.3253	-35.2	1.01E+03	1.1
y7+dye-2H	2	719.2605	719.2677	-10.0	1.75E+02	0.2
y20+dye-2H	3	853.6921	853.6857	7.5	6.31E+02	0.7

Supplementary Note 5. DMS, IMS and fluorescence spectroscopic measurements of doubly labelled isomeric α-helical peptides

Supplementary Note 5.1. Sample synthesis and purification

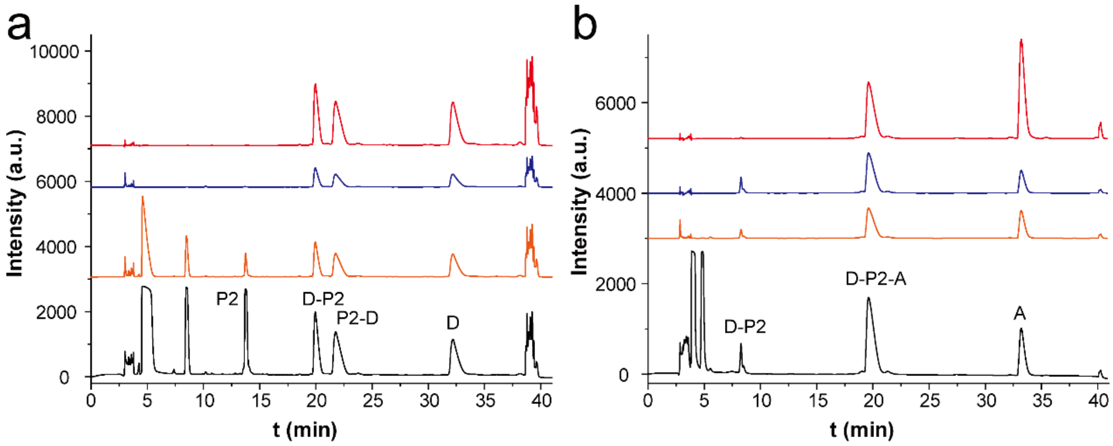

Supplementary Figure 25. HPLC purification of (a) singly and (b) doubly labelled **P2**. UV/VIS absorption spectroscopy was set at wavelengths of, (a) 220 nm (black), 235 nm (orange), 280 nm (blue), and 522 nm (red), and (b) 220 nm (black), 280 nm (orange), 505 nm (blue), and 575 nm (red) for detection. The denotation D-**P2**-A refers to the dye position after label. D refers to the cR6G and A to the QSY7.

Supplementary Note 5.2. Fluorescence spectroscopic experiments in the solution phase

Solution-phase FRET experiments of cR6G-**P1**-QSY7 and cR6G-**P2**-QSY7 were conducted in an in-plume setup by replacing the nano-electrospray tip with a cuvette.[8] The results showed two conformations from the double-exponential fit (Supplementary Figure 26 and Supplementary Table 9). Both cR6G-**P1**-QSY7 conformers exhibited a slightly lower lifetime as well as a higher ratio of molecules adopting compact conformations (58%) compared to cR6G-**P2**-QSY7 (40%).

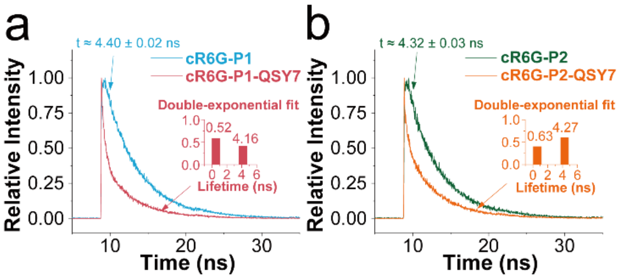

Supplementary Figure 26. Fluorescence decay curves of (a) cR6G-**P1**, cR6G-**P1**-QSY7, and (b) cR6G-**P2** and cR6G-**P2**-QSY7, which are dissolved in the water solution at pH values between 6.7-7.3. Laser was set to λex = 460 nm, P = 20 - 50 μW at a repetition rate of ~26.7 MHz. 20 s of fluorescence collection time was set in the single photon avalanche diode (SPAD). The decays were fitted with a single-exponential modified Gaussian function (without acceptor) or double-exponential fit (with acceptor).

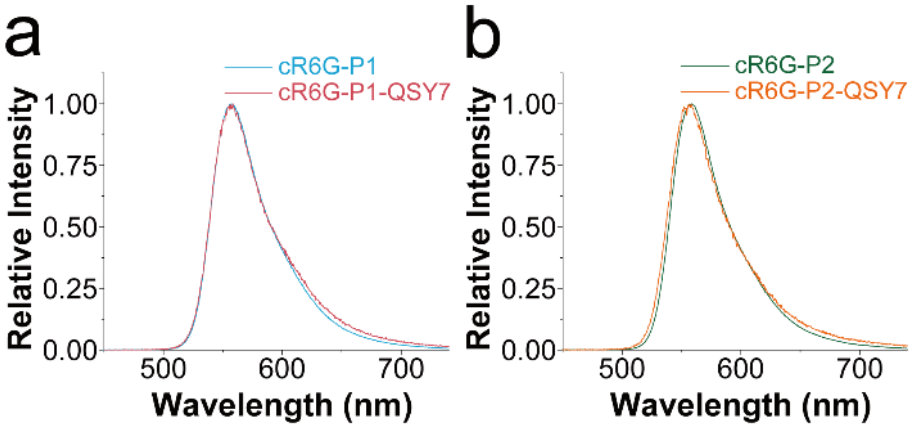

Supplementary Figure 27. Emission spectra of (a) cR6G-**P1**, cR6G-**P1**-QSY7, and (b) cR6G-**P2** and cR6G-**P2**-QSY7, which are dissolved in the water solution at pH values between 6.7-7.3. Laser was set to λex = 460 nm, P = 0.2 mW. 3 s of fluorescence collection time was set in the spectrograph.

Supplementary Table 9. Donor acceptor distance (r_{DA}) determination of cR6G-**P1**-QSY7 and cR6G-**P2**-QSY7 in the solution phase. R_0 for the FRET pair (cR6G and QSY7) was determined as 5.97 nm in solution. Details can be found in Methods.

Peptide	Lifetime (ns)	Ratio	E	r_{DA}(nm)
cR6G-**P1**-QSY7	0.52	0.58	0.88	4.27
	4.16	0.42	0.06	9.56
cR6G-**P2**-QSY7	0.63	0.40	0.85	4.44
	4.27	0.60	0.01	12.61

Supplementary Note 5.3. IM-MS experiments

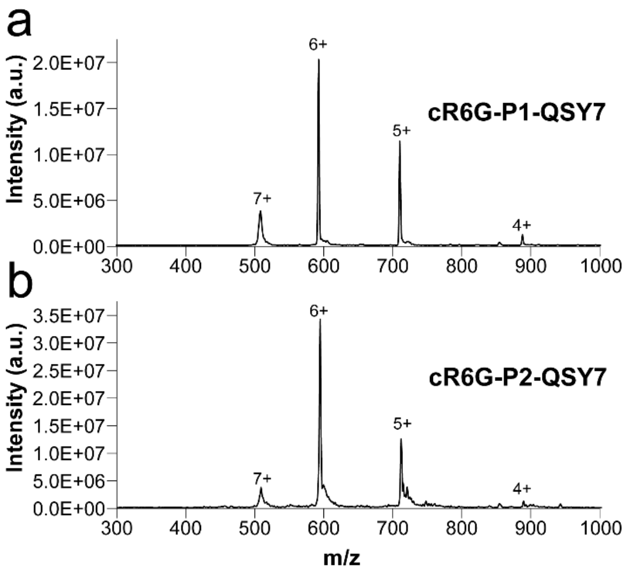

Supplementary Figure 28. nanoESI mass spectra of (a) cR6G-**P1**-QSY7, and (b) cR6G-**P2**-QSY7. 20 μM of the doubly labelled peptides in H_2O solution were sprayed in a ESI high voltage of ~1.0 kV.

Supplementary Table 10. Average CCS values determined at IMS wave heights of 7.0V, 7.5V, and 8.0V, respectively. Single or multiple peak fit with Gaussian function were applied in OriginPro 2021.

Ions	Charge states	Conformer	Mean	RSD (%)
cR6G-**P1**-QSY7	3+	1	776.7	0.9
		2	877.0	1.3
	4+	1	935.4	1.5
		2	975.3	1.3
	5+	1	1047.1	2.2
		2	1099.7	1.7
	6+	1	1111.6	2.4
		2	1168.3	2.7
	7+	1	1239.6	2.0
cR6G-**P2**-QSY7	3+	1	761.2	1.0
		2	876.7	1.1
	4+	1	940.8	1.2
		2	964.0	1.3
	5+	1	1048.3	1.8
		2	1085.4	1.6
	6+	1	1174.1	1.7
		2	1215.8	1.9
	7+	1	1179.3	1.7

Supplementary Note 5.4. Fluorescence spectroscopic meaurements

The lifetime measurements were conducted with λex = 460 nm, P = 10mW at a laser repetition rate of ~26.7 MHz, and 1800 s of fluorescence collection. These two charge states exhibit the same number of charges on the peptide chain, as QSY7 is likely singly charged. As the control experiments, the emission spectra showed no significant shift upon the addition of acceptor chromophore (Supplementary Figure 29). Moreover, the fluorescence decay curves of the $[M+6H]^{6+}$ ions of cR6G-**P1** and cR6G-**P2** (Supplementary Figure 30) confirmed that the decrease in fluorescence lifetime is not because of any photophysical changes of the dye due to the additional charge, but in fact due to FRET. The $[M+7H]^{7+}$ ion of cR6G-**P2**-QSY7 showed higher lifetime values, which indicated further unfolding of the ions (Supplementary Figure 31).

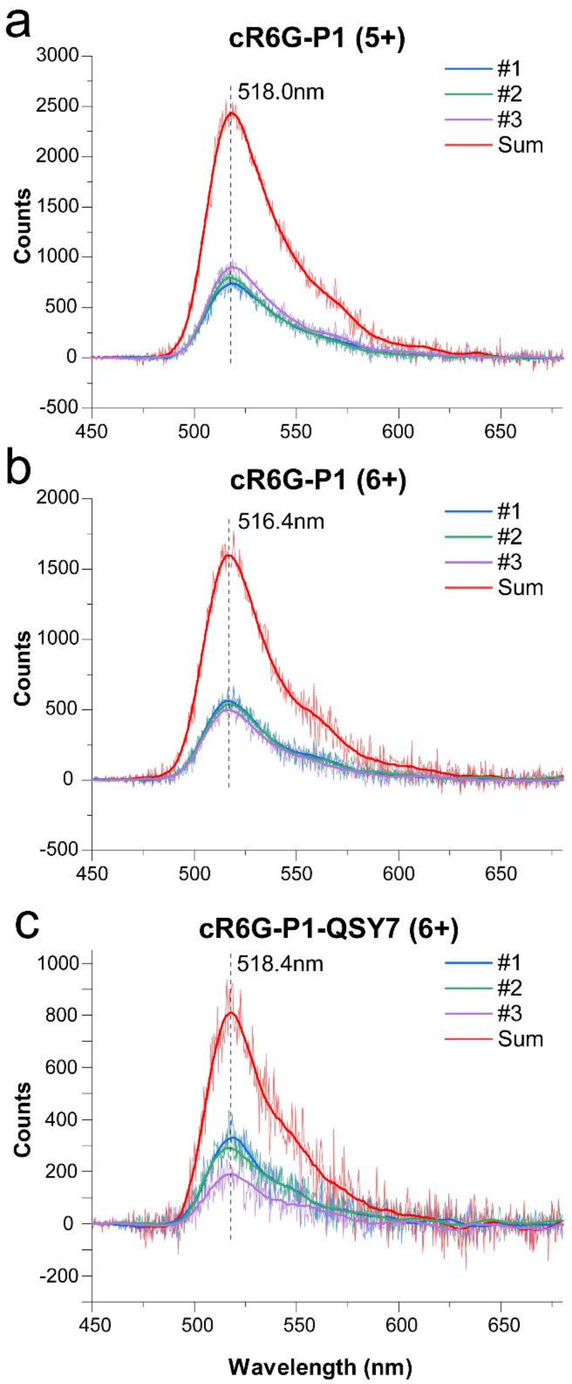

Supplementary Figure 29. Gas-phase fluorescence emission spectra of the (a) $[M+5H]^{5+}$ and (b) $[M+6H]^{6+}$ ions of cR6G-**P1**, and (c) $[M+6H]^{6+}$ ions of cR6G-**P1**-QSY7 with λ_{ex} = 460 nm, P = 10mW, and a fluorescence collection time of 540 s (i.e., 3*180 s). Three replicates were plotted to show the reproducibility of measurements.

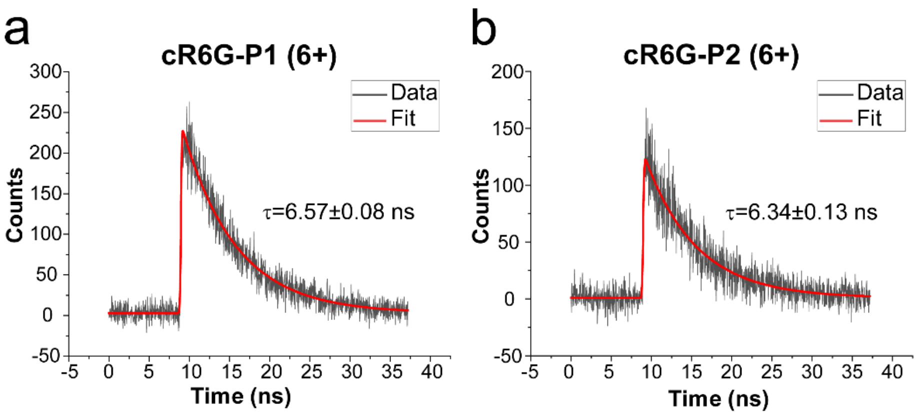

Supplementary Figure 30. Fluorescence decay curves of the $[M+6H]^{6+}$ ions of (a) cR6G-**P1**, and (b) cR6G-**P2** with λ_{ex} = 460 nm, P = 10mW at a repetition rate of ~26.7 MHz, and a fluorescence collection time of 1800 s (i.e., 3 * 600 s). The fluorescence decay curves were fitted with a single-exponential modified Gaussian function.

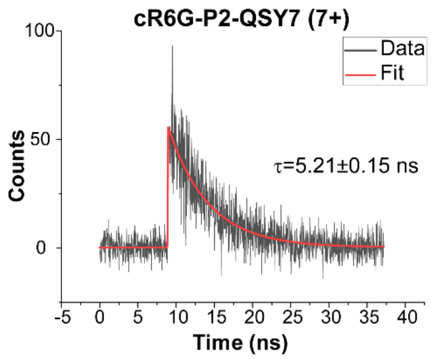

Supplementary Figure 31. Fluorescence decay curves of the $[M+7H]^{7+}$ ion of cR6G-**P2**-QSY7 with λ_{ex} = 460 nm, P = 10mW at a repetition rate of ~26.7 MHz, and a fluorescence collection time of 600 s. The fluorescence decay curve was fitted with a single-exponential modified Gaussian function.

Supplementary Note 5.5. Distance estimation from FRET efficiency (E_{FRET})

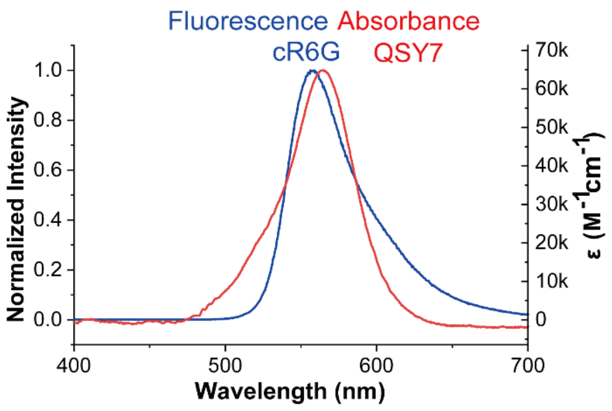

Supplementary Figure 32. Overlap of donor (cR6G) emission and acceptor (QSY7) absorption spectra in the solution phase.

Supplementary Note 5.6. Lifetime determination and multi-exponential fit of fluorescence decay

Supplementary Table 11. Results obtained from a single-exponential fit using Decay Fit and a pre-defined IRF.

Peptide ions	T (ns)
cR6G-P1 (5+)	6.39±0.24
cR6G-P1-QSY7 (6+)	3.92±0.11
cR6G-P2 (5+)	6.56±0.23
cR6G-P2-QSY7 (6+)	3.32±0.13

The results obtained for a double-exponential fit of the doubly labelled peptides are listed in Supplementary Table 12. The relative contributions of each lifetime value are also listed. We note that the quality of the fit did not improve significantly by the double-exponential fit.

Supplementary Table 12. Results obtained from a double-exponential fit using Decay Fit and a pre-defined IRF.

Peptide ions	T1 (ns)	T2 (ns)	rel. contribution T1
cR6G-P1-QSY7 (6+)	3.65	25.00	0.98
cR6G-P2-QSY7 (6+)	3.12	25.00	0.99

Supplementary Table 13. The calculated donor-acceptor distance of the doubly labelled peptides based on the FRET efficiencies measured in the gas phase. $R_{0,\ gas}$ was used for r_{DA} calculation.

Peptide ions	E	r_{DA}(Å)
cR6G-P1-QSY7 (6+)	0.40 ± 0.05	77.5 ± 2.5
cR6G-P2-QSY7 (6+)	0.52 ± 0.02	71.5 ± 0.9

Supplementary Note 5.7. Computational simulations

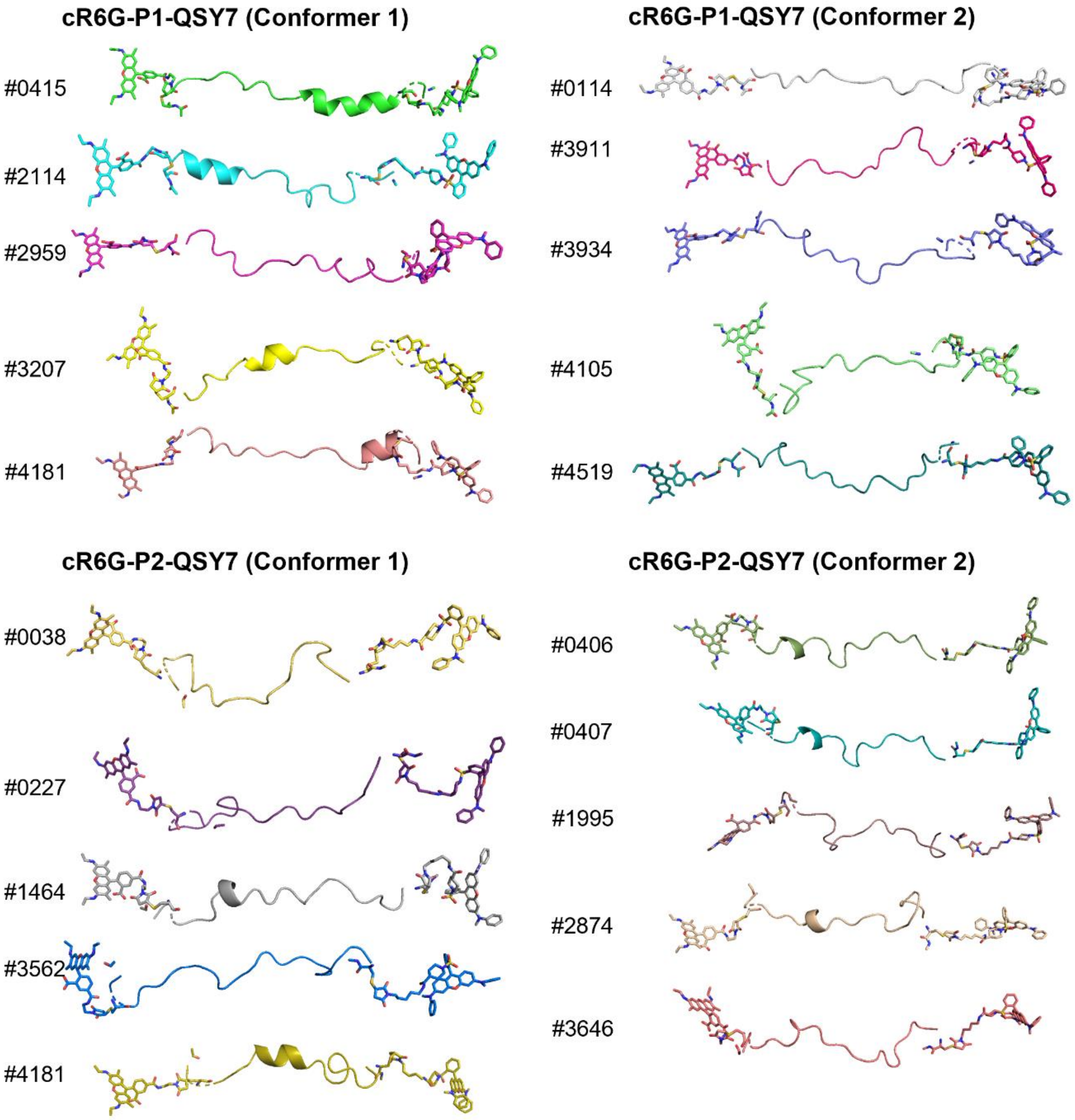

Supplementary Figure 33. Selected 5 structures for each conformer of the $[M+6H]^{6+}$ ion of two peptides. 5000 candidate structures generated from conformer distribution are filtered by their force field energies, IMS, and FRET results. The PDB structures are shown as cartoon and sticks.

Supplementary Table 14. The calculated parameters of selected 20 structures. CCS_TJM* values of each structure were calculated using IMPACT. CCS_TJM values were calculated using Collidoscope.

Peptide	Conf. #	#Frame / Structure	Energy [total, kcal/mol]	Distance (r_{DA}, Å)	CCS_TJM * (Å^2)	CCS_TJM (Å^2)	Solvation sites	Solvation free Energy (kcal/mol)
cR6G-**P1**-QSY7	1	415	103.5	67.0	1113.9	1108.9	8	-118
		2114	120.1	64.6	1120.7	1075.6	11	-176
		2959	114.7	66.9	1118.2	1074.0	14	-190
		3207	121.3	65.2	1130.5	1082.1	10	-154
		4181	109.3	69.1	1121.9	1085.6	13	-192
		**Average**	**113.8**	**66.5**	**1121.0**	**1085.2**	**11.2**	**-166**
	2	114	104.3	72.2	1166.2	1158.1	13	-180
		3911	107.0	70.1	1161.0	1111.8	11	-147
		3934	122.1	70.8	1165.0	1094.7	9	-129
		4107	115.0	72.4	1185.6	1117.2	10	-162
		4519	120.8	73.0	1187.2	1118.3	9	-134
		**Average**	**116.9**	**71.5**	**1178.6**	**1120.0**	**10.4**	**-150**
cR6G-**P2**-QSY7	1	38	122.9	67.0	1173.2	1100.7	8	-114
		227	116.5	66.6	1176.3	1107.1	8	-119
		1464	120.5	67.4	1161.6	1097.9	7	-113
		3562	133.7	70.0	1172.5	1110.1	7	-110
		4962	131.6	68.7	1173.1	1155.4	8	-117
		**Average**	**125.0**	**66.6**	**1171.4**	**1114.1**	**7.6**	**-115**
	2	406	132.4	68.6	1194.1	1127.7	10	-153
		407	131.5	69.3	1201.7	1133.2	9	-141
		1995	126.1	68.5	1199.2	1112.2	8	-128
		2874	131.9	70.5	1225.6	1165.0	6	-92
		3646	123.3	68.5	1192.8	1117.4	5	-85
		**Average**	**129.0**	**69.1**	**1202.7**	**1131.1**	**7.6**	**-120**

Supplementary Table 15. List of atoms and their coordination in the structure #415 of cR6G-P1-QSY7.

ATOM	1	H1	ACE	1	3.691	23.773	-5.213	1.00	0.00	H
ATOM	2	CH3	ACE	1	3.855	24.701	-5.759	1.00	0.00	C
ATOM	3	H2	ACE	1	4.299	25.437	-5.090	1.00	0.00	H
ATOM	4	H3	ACE	1	2.906	25.084	-6.132	1.00	0.00	H
ATOM	5	C	ACE	1	4.792	24.451	-6.918	1.00	0.00	C
ATOM	6	O	ACE	1	5.909	24.949	-6.930	1.00	0.00	O
ATOM	7	N	QS	2	4.419	23.622	-7.941	1.00	0.00	N
ATOM	8	C1	QS	2	3.341	22.605	-7.890	1.00	0.00	C
ATOM	9	C2	QS	2	3.849	21.243	-7.356	1.00	0.00	C
ATOM	10	C3	QS	2	2.561	22.491	-9.230	1.00	0.00	C
ATOM	11	O1	QS	2	3.265	20.658	-6.468	1.00	0.00	O
ATOM	12	S	QS	2	3.487	21.682	-10.618	1.00	0.00	S
ATOM	13	C4	QS	2	4.888	15.756	-13.239	1.00	0.00	C
ATOM	14	C5	QS	2	5.801	16.298	-12.365	1.00	0.00	C
ATOM	15	C6	QS	2	5.969	17.724	-12.242	1.00	0.00	C
ATOM	16	C7	QS	2	5.127	18.565	-13.066	1.00	0.00	C
ATOM	17	C8	QS	2	4.190	18.053	-13.955	1.00	0.00	C
ATOM	18	C9	QS	2	4.048	16.605	-14.040	1.00	0.00	C
ATOM	19	C10	QS	2	6.894	18.239	-11.367	1.00	0.00	C
ATOM	20	N2	QS	2	7.182	19.588	-11.196	1.00	0.00	N
ATOM	21	O2	QS	2	7.558	17.545	-10.614	1.00	0.00	O
ATOM	22	C11	QS	2	7.995	20.283	-12.206	1.00	0.00	C
ATOM	23	C12	QS	2	8.054	21.811	-11.967	1.00	0.00	C
ATOM	24	N3	QS	2	6.714	22.433	-11.999	1.00	0.00	N
ATOM	25	C13	QS	2	6.008	22.773	-10.889	1.00	0.00	C
ATOM	26	C14	QS	2	4.557	23.050	-11.266	1.00	0.00	C
ATOM	27	C15	QS	2	4.591	23.035	-12.821	1.00	0.00	C
ATOM	28	C16	QS	2	5.975	22.507	-13.141	1.00	0.00	C
ATOM	29	O3	QS	2	6.371	22.157	-14.231	1.00	0.00	O
ATOM	30	O4	QS	2	6.455	22.819	-9.756	1.00	0.00	O
ATOM	31	C17	QS	2	3.076	15.989	-14.827	1.00	0.00	C
ATOM	32	C18	QS	2	3.469	14.850	-15.665	1.00	0.00	C
ATOM	33	C19	QS	2	2.450	14.042	-16.185	1.00	0.00	C
ATOM	34	O5	QS	2	1.136	14.304	-15.903	1.00	0.00	O
ATOM	35	C20	QS	2	0.733	15.291	-15.040	1.00	0.00	C
ATOM	36	C21	QS	2	1.664	16.152	-14.440	1.00	0.00	C
ATOM	37	C22	QS	2	-0.624	15.375	-14.737	1.00	0.00	C
ATOM	38	C23	QS	2	-1.089	16.308	-13.808	1.00	0.00	C
ATOM	39	C24	QS	2	-0.168	17.154	-13.156	1.00	0.00	C
ATOM	40	C25	QS	2	1.203	17.064	-13.465	1.00	0.00	C
ATOM	41	C26	QS	2	4.797	14.613	-16.076	1.00	0.00	C
ATOM	42	C27	QS	2	5.103	13.534	-16.928	1.00	0.00	C
ATOM	43	C28	QS	2	4.066	12.700	-17.402	1.00	0.00	C
ATOM	44	C29	QS	2	2.744	12.973	-17.031	1.00	0.00	C
ATOM	45	C30	QS	2	6.558	13.301	-17.340	1.00	0.00	C
ATOM	46	C31	QS	2	-0.630	18.167	-12.107	1.00	0.00	C
ATOM	47	N4	QS	2	-2.441	16.392	-13.506	1.00	0.00	N
ATOM	48	N5	QS	2	4.374	11.642	-18.249	1.00	0.00	N
ATOM	49	C32	QS	2	-3.506	15.585	-14.120	1.00	0.00	C
ATOM	50	C33	QS	2	-4.872	16.000	-13.550	1.00	0.00	C
ATOM	51	C34	QS	2	3.427	10.662	-18.809	1.00	0.00	C
ATOM	52	C35	QS	2	2.784	11.218	-20.093	1.00	0.00	C
ATOM	53	C36	QS	2	3.448	19.048	-14.794	1.00	0.00	C
ATOM	54	O7	QS	2	3.468	20.247	-14.616	1.00	0.00	O
ATOM	55	O8	QS	2	2.782	18.574	-15.844	1.00	0.00	O
ATOM	56	H1	QS	2	5.164	23.462	-8.611	1.00	0.00	H
ATOM	57	H2	QS	2	2.601	22.922	-7.144	1.00	0.00	H
ATOM	58	H3	QS	2	1.666	21.881	-9.045	1.00	0.00	H
ATOM	59	H4	QS	2	2.214	23.483	-9.547	1.00	0.00	H
ATOM	60	H6	QS	2	4.768	14.677	-13.283	1.00	0.00	H
ATOM	61	H7	QS	2	6.411	15.636	-11.755	1.00	0.00	H
ATOM	62	H8	QS	2	5.248	19.642	-12.994	1.00	0.00	H
ATOM	63	H9	QS	2	7.429	19.796	-10.231	1.00	0.00	H
ATOM	64	H10	QS	2	9.015	19.877	-12.177	1.00	0.00	H
ATOM	65	H11	QS	2	7.593	20.088	-13.210	1.00	0.00	H
ATOM	66	H12	QS	2	8.534	22.007	-10.998	1.00	0.00	H
ATOM	67	H13	QS	2	8.677	22.268	-12.748	1.00	0.00	H
ATOM	68	H14	QS	2	4.259	24.031	-10.877	1.00	0.00	H
ATOM	69	H15	QS	2	4.498	24.041	-13.246	1.00	0.00	H
ATOM	70	H16	QS	2	3.830	22.381	-13.261	1.00	0.00	H
ATOM	71	H17	QS	2	-1.301	14.689	-15.231	1.00	0.00	H
ATOM	72	H18	QS	2	1.896	17.703	-12.927	1.00	0.00	H
ATOM	73	H19	QS	2	5.598	15.275	-15.753	1.00	0.00	H
ATOM	74	H20	QS	2	1.921	12.378	-17.404	1.00	0.00	H

ATOM	75	H21	QS	2	6.679	13.384	-18.428	1.00	0.00	H
ATOM	76	H22	QS	2	7.236	14.033	-16.882	1.00	0.00	H
ATOM	77	H23	QS	2	6.902	12.305	-17.033	1.00	0.00	H
ATOM	78	H24	QS	2	-1.114	17.665	-11.259	1.00	0.00	H
ATOM	79	H25	QS	2	0.208	18.751	-11.704	1.00	0.00	H
ATOM	80	H26	QS	2	-1.346	18.880	-12.535	1.00	0.00	H
ATOM	81	H27	QS	2	-2.711	17.071	-12.809	1.00	0.00	H
ATOM	82	H28	QS	2	5.349	11.532	-18.494	1.00	0.00	H
ATOM	83	H29	QS	2	-3.510	15.728	-15.209	1.00	0.00	H
ATOM	84	H30	QS	2	-3.338	14.518	-13.915	1.00	0.00	H
ATOM	85	H31	QS	2	-5.672	15.402	-14.003	1.00	0.00	H
ATOM	86	H32	QS	2	-4.908	15.845	-12.464	1.00	0.00	H
ATOM	87	H33	QS	2	-5.080	17.057	-13.759	1.00	0.00	H
ATOM	88	H34	QS	2	3.958	9.732	-19.052	1.00	0.00	H
ATOM	89	H35	QS	2	2.647	10.406	-18.079	1.00	0.00	H
ATOM	90	H36	QS	2	3.550	11.435	-20.849	1.00	0.00	H
ATOM	91	H37	QS	2	2.084	10.488	-20.518	1.00	0.00	H
ATOM	92	H38	QS	2	2.231	12.143	-19.890	1.00	0.00	H
ATOM	93	H40	QS	2	2.484	19.413	-16.236	1.00	0.00	H
ATOM	94	N	ALA	3	5.026	20.747	-7.882	1.00	0.00	N
ATOM	95	H	ALA	3	5.518	21.287	-8.579	1.00	0.00	H
ATOM	96	CA	ALA	3	5.624	19.471	-7.489	1.00	0.00	C
ATOM	97	HA	ALA	3	5.416	19.301	-6.431	1.00	0.00	H
ATOM	98	CB	ALA	3	4.949	18.348	-8.291	1.00	0.00	C
ATOM	99	HB1	ALA	3	5.044	18.539	-9.358	1.00	0.00	H
ATOM	100	HB2	ALA	3	5.408	17.388	-8.055	1.00	0.00	H
ATOM	101	HB3	ALA	3	3.889	18.301	-8.034	1.00	0.00	H
ATOM	102	C	ALA	3	7.157	19.470	-7.662	1.00	0.00	C
ATOM	103	O	ALA	3	7.696	20.272	-8.430	1.00	0.00	O
ATOM	104	N	ALA	4	7.827	18.544	-6.965	1.00	0.00	N
ATOM	105	H	ALA	4	7.272	17.918	-6.404	1.00	0.00	H
ATOM	106	CA	ALA	4	9.285	18.367	-6.887	1.00	0.00	C
ATOM	107	HA	ALA	4	9.448	17.544	-6.191	1.00	0.00	H
ATOM	108	CB	ALA	4	9.827	17.886	-8.242	1.00	0.00	C
ATOM	109	HB1	ALA	4	9.730	18.673	-8.991	1.00	0.00	H
ATOM	110	HB2	ALA	4	10.879	17.614	-8.153	1.00	0.00	H
ATOM	111	HB3	ALA	4	9.269	17.009	-8.574	1.00	0.00	H
ATOM	112	C	ALA	4	10.078	19.561	-6.301	1.00	0.00	C
ATOM	113	O	ALA	4	9.586	20.682	-6.170	1.00	0.00	O
ATOM	114	N	ALA	5	11.336	19.304	-5.924	1.00	0.00	N
ATOM	115	H	ALA	5	11.698	18.375	-6.073	1.00	0.00	H
ATOM	116	CA	ALA	5	12.248	20.303	-5.363	1.00	0.00	C
ATOM	117	HA	ALA	5	11.774	20.737	-4.480	1.00	0.00	H
ATOM	118	CB	ALA	5	13.532	19.592	-4.915	1.00	0.00	C
ATOM	119	HB1	ALA	5	14.034	19.148	-5.776	1.00	0.00	H
ATOM	120	HB2	ALA	5	14.207	20.305	-4.439	1.00	0.00	H
ATOM	121	HB3	ALA	5	13.291	18.808	-4.195	1.00	0.00	H
ATOM	122	C	ALA	5	12.547	21.454	-6.348	1.00	0.00	C
ATOM	123	O	ALA	5	12.371	21.324	-7.562	1.00	0.00	O
ATOM	124	N	HIP	6	12.988	22.611	-5.838	1.00	0.00	N
ATOM	125	H	HIP	6	13.164	22.658	-4.843	1.00	0.00	H
ATOM	126	CA	HIP	6	13.338	23.801	-6.636	1.00	0.00	C
ATOM	127	HA	HIP	6	13.474	23.509	-7.678	1.00	0.00	H
ATOM	128	CB	HIP	6	12.217	24.864	-6.569	1.00	0.00	C
ATOM	129	HB2	HIP	6	12.174	25.241	-5.544	1.00	0.00	H
ATOM	130	HB3	HIP	6	12.499	25.705	-7.205	1.00	0.00	H
ATOM	131	CG	HIP	6	10.812	24.440	-6.960	1.00	0.00	C
ATOM	132	ND1	HIP	6	10.422	23.297	-7.642	1.00	0.00	N
ATOM	133	HD1	HIP	6	11.026	22.504	-7.870	1.00	0.00	H
ATOM	134	CE1	HIP	6	9.079	23.294	-7.735	1.00	0.00	C
ATOM	135	HE1	HIP	6	8.474	22.474	-8.115	1.00	0.00	H
ATOM	136	NE2	HIP	6	8.607	24.421	-7.169	1.00	0.00	N
ATOM	137	HE2	HIP	6	7.615	24.661	-7.060	1.00	0.00	H
ATOM	138	CD2	HIP	6	9.675	25.148	-6.678	1.00	0.00	C
ATOM	139	HD2	HIP	6	9.618	26.088	-6.141	1.00	0.00	H
ATOM	140	C	HIP	6	14.673	24.390	-6.153	1.00	0.00	C
ATOM	141	O	HIP	6	14.980	24.309	-4.967	1.00	0.00	O
ATOM	142	N	ALA	7	15.453	24.988	-7.055	1.00	0.00	N
ATOM	143	H	ALA	7	15.142	25.043	-8.010	1.00	0.00	H
ATOM	144	CA	ALA	7	16.721	25.640	-6.712	1.00	0.00	C
ATOM	145	HA	ALA	7	17.306	24.948	-6.102	1.00	0.00	H
ATOM	146	CB	ALA	7	17.500	25.909	-8.005	1.00	0.00	C
ATOM	147	HB1	ALA	7	18.470	26.350	-7.767	1.00	0.00	H
ATOM	148	HB2	ALA	7	17.665	24.975	-8.544	1.00	0.00	H
ATOM	149	HB3	ALA	7	16.945	26.601	-8.640	1.00	0.00	H
ATOM	150	C	ALA	7	16.519	26.933	-5.893	1.00	0.00	C

ATOM	151	O	ALA	7	15.520	27.635	-6.058	1.00	0.00	O
ATOM	152	N	ALA	8	17.485	27.270	-5.032	1.00	0.00	N
ATOM	153	H	ALA	8	18.289	26.668	-4.946	1.00	0.00	H
ATOM	154	CA	ALA	8	17.450	28.485	-4.219	1.00	0.00	C
ATOM	155	HA	ALA	8	16.471	28.540	-3.738	1.00	0.00	H
ATOM	156	CB	ALA	8	18.511	28.373	-3.118	1.00	0.00	C
ATOM	157	HB1	ALA	8	19.506	28.307	-3.560	1.00	0.00	H
ATOM	158	HB2	ALA	8	18.466	29.250	-2.470	1.00	0.00	H
ATOM	159	HB3	ALA	8	18.326	27.483	-2.514	1.00	0.00	H
ATOM	160	C	ALA	8	17.641	29.761	-5.067	1.00	0.00	C
ATOM	161	O	ALA	8	18.564	29.858	-5.875	1.00	0.00	O
ATOM	162	N	ALA	9	16.811	30.782	-4.835	1.00	0.00	N
ATOM	163	H	ALA	9	16.081	30.637	-4.154	1.00	0.00	H
ATOM	164	CA	ALA	9	16.704	31.995	-5.660	1.00	0.00	C
ATOM	165	HA	ALA	9	16.664	31.670	-6.701	1.00	0.00	H
ATOM	166	CB	ALA	9	15.351	32.650	-5.337	1.00	0.00	C
ATOM	167	HB1	ALA	9	15.349	33.024	-4.312	1.00	0.00	H
ATOM	168	HB2	ALA	9	15.170	33.484	-6.017	1.00	0.00	H
ATOM	169	HB3	ALA	9	14.545	31.925	-5.461	1.00	0.00	H
ATOM	170	C	ALA	9	17.886	33.001	-5.578	1.00	0.00	C
ATOM	171	O	ALA	9	17.703	34.181	-5.871	1.00	0.00	O
ATOM	172	N	HIP	10	19.091	32.564	-5.175	1.00	0.00	N
ATOM	173	H	HIP	10	19.164	31.559	-5.057	1.00	0.00	H
ATOM	174	CA	HIP	10	20.351	33.341	-5.135	1.00	0.00	C
ATOM	175	HA	HIP	10	20.995	32.859	-4.398	1.00	0.00	H
ATOM	176	CB	HIP	10	21.095	33.215	-6.487	1.00	0.00	C
ATOM	177	HB2	HIP	10	22.021	33.791	-6.431	1.00	0.00	H
ATOM	178	HB3	HIP	10	21.381	32.170	-6.619	1.00	0.00	H
ATOM	179	CG	HIP	10	20.342	33.656	-7.726	1.00	0.00	C
ATOM	180	ND1	HIP	10	19.716	34.873	-7.899	1.00	0.00	N
ATOM	181	HD1	HIP	10	19.634	35.575	-7.159	1.00	0.00	H
ATOM	182	CE1	HIP	10	19.174	34.906	-9.132	1.00	0.00	C
ATOM	183	HE1	HIP	10	18.595	35.728	-9.545	1.00	0.00	H
ATOM	184	NE2	HIP	10	19.458	33.741	-9.755	1.00	0.00	N
ATOM	185	HE2	HIP	10	19.187	33.490	-10.702	1.00	0.00	H
ATOM	186	CD2	HIP	10	20.189	32.945	-8.885	1.00	0.00	C
ATOM	187	HD2	HIP	10	20.577	31.951	-9.081	1.00	0.00	H
ATOM	188	C	HIP	10	20.188	34.792	-4.617	1.00	0.00	C
ATOM	189	O	HIP	10	20.324	35.757	-5.371	1.00	0.00	O
ATOM	190	N	ALA	11	19.846	34.939	-3.335	1.00	0.00	N
ATOM	191	H	ALA	11	19.748	34.113	-2.769	1.00	0.00	H
ATOM	192	CA	ALA	11	19.615	36.236	-2.692	1.00	0.00	C
ATOM	193	HA	ALA	11	18.854	36.765	-3.268	1.00	0.00	H
ATOM	194	CB	ALA	11	19.054	35.984	-1.286	1.00	0.00	C
ATOM	195	HB1	ALA	11	19.792	35.458	-0.678	1.00	0.00	H
ATOM	196	HB2	ALA	11	18.813	36.933	-0.805	1.00	0.00	H
ATOM	197	HB3	ALA	11	18.144	35.385	-1.348	1.00	0.00	H
ATOM	198	C	ALA	11	20.873	37.135	-2.647	1.00	0.00	C
ATOM	199	O	ALA	11	22.003	36.650	-2.667	1.00	0.00	O
ATOM	200	N	ALA	12	20.671	38.455	-2.548	1.00	0.00	N
ATOM	201	H	ALA	12	19.718	38.779	-2.512	1.00	0.00	H
ATOM	202	CA	ALA	12	21.715	39.488	-2.593	1.00	0.00	C
ATOM	203	HA	ALA	12	22.404	39.225	-3.398	1.00	0.00	H
ATOM	204	CB	ALA	12	21.044	40.815	-2.980	1.00	0.00	C
ATOM	205	HB1	ALA	12	20.509	40.701	-3.924	1.00	0.00	H
ATOM	206	HB2	ALA	12	20.345	41.124	-2.202	1.00	0.00	H
ATOM	207	HB3	ALA	12	21.803	41.589	-3.104	1.00	0.00	H
ATOM	208	C	ALA	12	22.570	39.594	-1.302	1.00	0.00	C
ATOM	209	O	ALA	12	22.662	40.655	-0.677	1.00	0.00	O
ATOM	210	N	ALA	13	23.221	38.494	-0.905	1.00	0.00	N
ATOM	211	H	ALA	13	23.088	37.653	-1.458	1.00	0.00	H
ATOM	212	CA	ALA	13	24.002	38.373	0.334	1.00	0.00	C
ATOM	213	HA	ALA	13	23.324	38.493	1.180	1.00	0.00	H
ATOM	214	CB	ALA	13	24.580	36.954	0.384	1.00	0.00	C
ATOM	215	HB1	ALA	13	23.775	36.219	0.335	1.00	0.00	H
ATOM	216	HB2	ALA	13	25.265	36.795	-0.451	1.00	0.00	H
ATOM	217	HB3	ALA	13	25.125	36.814	1.319	1.00	0.00	H
ATOM	218	C	ALA	13	25.114	39.433	0.494	1.00	0.00	C
ATOM	219	O	ALA	13	25.445	39.810	1.615	1.00	0.00	O
ATOM	220	N	ALA	14	25.622	39.998	-0.610	1.00	0.00	N
ATOM	221	H	ALA	14	25.316	39.643	-1.502	1.00	0.00	H
ATOM	222	CA	ALA	14	26.591	41.098	-0.610	1.00	0.00	C
ATOM	223	HA	ALA	14	27.518	40.740	-0.157	1.00	0.00	H
ATOM	224	CB	ALA	14	26.883	41.468	-2.069	1.00	0.00	C
ATOM	225	HB1	ALA	14	27.640	42.254	-2.101	1.00	0.00	H
ATOM	226	HB2	ALA	14	27.264	40.599	-2.607	1.00	0.00	H

ATOM	227	HB3	ALA	14	25.977	41.831	-2.557	1.00	0.00	H
ATOM	228	C	ALA	14	26.144	42.338	0.200	1.00	0.00	C
ATOM	229	O	ALA	14	26.988	43.108	0.654	1.00	0.00	O
ATOM	230	N	HIP	15	24.840	42.508	0.477	1.00	0.00	N
ATOM	231	H	HIP	15	24.181	41.839	0.090	1.00	0.00	H
ATOM	232	CA	HIP	15	24.335	43.549	1.392	1.00	0.00	C
ATOM	233	HA	HIP	15	24.662	44.513	0.999	1.00	0.00	H
ATOM	234	CB	HIP	15	22.789	43.518	1.381	1.00	0.00	C
ATOM	235	HB2	HIP	15	22.457	43.346	0.355	1.00	0.00	H
ATOM	236	HB3	HIP	15	22.443	42.669	1.976	1.00	0.00	H
ATOM	237	CG	HIP	15	22.098	44.778	1.874	1.00	0.00	C
ATOM	238	ND1	HIP	15	22.459	45.556	2.961	1.00	0.00	N
ATOM	239	HD1	HIP	15	23.293	45.394	3.536	1.00	0.00	H
ATOM	240	CE1	HIP	15	21.576	46.567	3.082	1.00	0.00	C
ATOM	241	HE1	HIP	15	21.610	47.348	3.838	1.00	0.00	H
ATOM	242	NE2	HIP	15	20.654	46.449	2.103	1.00	0.00	N
ATOM	243	HE2	HIP	15	19.865	47.073	1.950	1.00	0.00	H
ATOM	244	CD2	HIP	15	20.965	45.333	1.340	1.00	0.00	C
ATOM	245	HD2	HIP	15	20.415	44.960	0.483	1.00	0.00	H
ATOM	246	C	HIP	15	24.920	43.442	2.822	1.00	0.00	C
ATOM	247	O	HIP	15	24.826	44.409	3.577	1.00	0.00	O
ATOM	248	N	ALA	16	25.519	42.311	3.206	1.00	0.00	N
ATOM	249	H	ALA	16	25.531	41.524	2.567	1.00	0.00	H
ATOM	250	CA	ALA	16	26.215	42.134	4.483	1.00	0.00	C
ATOM	251	HA	ALA	16	25.589	42.534	5.282	1.00	0.00	H
ATOM	252	CB	ALA	16	26.373	40.626	4.718	1.00	0.00	C
ATOM	253	HB1	ALA	16	25.399	40.137	4.691	1.00	0.00	H
ATOM	254	HB2	ALA	16	27.017	40.190	3.951	1.00	0.00	H
ATOM	255	HB3	ALA	16	26.824	40.453	5.696	1.00	0.00	H
ATOM	256	C	ALA	16	27.583	42.851	4.583	1.00	0.00	C
ATOM	257	O	ALA	16	28.127	42.939	5.680	1.00	0.00	O
ATOM	258	N	ALA	17	28.146	43.373	3.483	1.00	0.00	N
ATOM	259	H	ALA	17	27.650	43.284	2.605	1.00	0.00	H
ATOM	260	CA	ALA	17	29.525	43.885	3.394	1.00	0.00	C
ATOM	261	HA	ALA	17	30.164	43.132	3.856	1.00	0.00	H
ATOM	262	CB	ALA	17	29.907	43.953	1.908	1.00	0.00	C
ATOM	263	HB1	ALA	17	29.304	44.709	1.403	1.00	0.00	H
ATOM	264	HB2	ALA	17	30.961	44.216	1.807	1.00	0.00	H
ATOM	265	HB3	ALA	17	29.743	42.985	1.434	1.00	0.00	H
ATOM	266	C	ALA	17	29.831	45.211	4.149	1.00	0.00	C
ATOM	267	O	ALA	17	30.715	45.974	3.756	1.00	0.00	O
ATOM	268	N	ALA	18	29.120	45.507	5.241	1.00	0.00	N
ATOM	269	H	ALA	18	28.475	44.803	5.577	1.00	0.00	H
ATOM	270	CA	ALA	18	29.321	46.704	6.066	1.00	0.00	C
ATOM	271	HA	ALA	18	29.237	47.584	5.426	1.00	0.00	H
ATOM	272	CB	ALA	18	28.195	46.750	7.105	1.00	0.00	C
ATOM	273	HB1	ALA	18	27.226	46.764	6.605	1.00	0.00	H
ATOM	274	HB2	ALA	18	28.251	45.879	7.760	1.00	0.00	H
ATOM	275	HB3	ALA	18	28.293	47.654	7.709	1.00	0.00	H
ATOM	276	C	ALA	18	30.709	46.764	6.741	1.00	0.00	C
ATOM	277	O	ALA	18	31.175	47.846	7.103	1.00	0.00	O
ATOM	278	N	HIE	19	31.410	45.628	6.855	1.00	0.00	N
ATOM	279	H	HIE	19	31.007	44.779	6.480	1.00	0.00	H
ATOM	280	CA	HIE	19	32.787	45.547	7.362	1.00	0.00	C
ATOM	281	HA	HIE	19	32.810	45.950	8.375	1.00	0.00	H
ATOM	282	CB	HIE	19	33.219	44.074	7.414	1.00	0.00	C
ATOM	283	HB2	HIE	19	34.147	44.005	7.982	1.00	0.00	H
ATOM	284	HB3	HIE	19	32.462	43.490	7.940	1.00	0.00	H
ATOM	285	CG	HIE	19	33.443	43.470	6.050	1.00	0.00	C
ATOM	286	ND1	HIE	19	32.460	43.283	5.078	1.00	0.00	N
ATOM	287	CE1	HIE	19	33.093	42.772	4.012	1.00	0.00	C
ATOM	288	HE1	HIE	19	32.611	42.506	3.080	1.00	0.00	H
ATOM	289	NE2	HIE	19	34.406	42.636	4.263	1.00	0.00	N
ATOM	290	HE2	HIE	19	35.093	42.267	3.619	1.00	0.00	H
ATOM	291	CD2	HIE	19	34.647	43.077	5.544	1.00	0.00	C
ATOM	292	HD2	HIE	19	35.600	43.119	6.056	1.00	0.00	H
ATOM	293	C	HIE	19	33.787	46.368	6.531	1.00	0.00	C
ATOM	294	O	HIE	19	34.852	46.712	7.038	1.00	0.00	O
ATOM	295	N	ALA	20	33.434	46.762	5.300	1.00	0.00	N
ATOM	296	H	ALA	20	32.562	46.413	4.919	1.00	0.00	H
ATOM	297	CA	ALA	20	34.203	47.703	4.485	1.00	0.00	C
ATOM	298	HA	ALA	20	35.163	47.243	4.245	1.00	0.00	H
ATOM	299	CB	ALA	20	33.435	47.931	3.179	1.00	0.00	C
ATOM	300	HB1	ALA	20	34.006	48.595	2.528	1.00	0.00	H
ATOM	301	HB2	ALA	20	33.283	46.980	2.667	1.00	0.00	H
ATOM	302	HB3	ALA	20	32.464	48.382	3.388	1.00	0.00	H

ATOM	303	C	ALA	20	34.502	49.040	5.203	1.00	0.00	C
ATOM	304	O	ALA	20	35.508	49.683	4.897	1.00	0.00	O
ATOM	305	N	ALA	21	33.711	49.427	6.216	1.00	0.00	N
ATOM	306	H	ALA	21	32.897	48.861	6.431	1.00	0.00	H
ATOM	307	CA	ALA	21	33.989	50.570	7.095	1.00	0.00	C
ATOM	308	HA	ALA	21	33.977	51.478	6.492	1.00	0.00	H
ATOM	309	CB	ALA	21	32.852	50.658	8.119	1.00	0.00	C
ATOM	310	HB1	ALA	21	32.832	49.758	8.737	1.00	0.00	H
ATOM	311	HB2	ALA	21	33.000	51.527	8.761	1.00	0.00	H
ATOM	312	HB3	ALA	21	31.894	50.757	7.606	1.00	0.00	H
ATOM	313	C	ALA	21	35.368	50.506	7.798	1.00	0.00	C
ATOM	314	O	ALA	21	35.908	51.545	8.186	1.00	0.00	O
ATOM	315	N	ALA	22	35.980	49.318	7.888	1.00	0.00	N
ATOM	316	H	ALA	22	35.477	48.494	7.574	1.00	0.00	H
ATOM	317	CA	ALA	22	37.361	49.114	8.329	1.00	0.00	C
ATOM	318	HA	ALA	22	37.447	49.480	9.352	1.00	0.00	H
ATOM	319	CB	ALA	22	37.652	47.609	8.341	1.00	0.00	C
ATOM	320	HB1	ALA	22	38.656	47.432	8.729	1.00	0.00	H
ATOM	321	HB2	ALA	22	36.933	47.096	8.982	1.00	0.00	H
ATOM	322	HB3	ALA	22	37.585	47.205	7.330	1.00	0.00	H
ATOM	323	C	ALA	22	38.404	49.878	7.484	1.00	0.00	C
ATOM	324	O	ALA	22	39.526	50.068	7.948	1.00	0.00	O
ATOM	325	N	Y7	23	38.069	50.265	6.196	1.00	0.00	N
ATOM	326	C1	Y7	23	38.575	51.460	5.467	1.00	0.00	C
ATOM	327	C2	Y7	23	39.776	52.179	6.148	1.00	0.00	C
ATOM	328	C3	Y7	23	37.414	52.462	5.210	1.00	0.00	C
ATOM	329	O1	Y7	23	39.642	53.212	6.787	1.00	0.00	O
ATOM	330	S	Y7	23	37.878	53.631	3.857	1.00	0.00	S
ATOM	331	C4	Y7	23	42.730	61.639	8.733	1.00	0.00	C
ATOM	332	C5	Y7	23	52.171	57.269	4.924	1.00	0.00	C
ATOM	333	O2	Y7	23	37.847	55.932	6.341	1.00	0.00	O
ATOM	334	O3	Y7	23	37.847	57.947	2.281	1.00	0.00	O
ATOM	335	C6	Y7	23	37.569	57.138	3.140	1.00	0.00	C
ATOM	336	C7	Y7	23	36.622	55.966	2.970	1.00	0.00	C
ATOM	337	C8	Y7	23	36.853	55.124	4.255	1.00	0.00	C
ATOM	338	C9	Y7	23	37.645	56.080	5.149	1.00	0.00	C
ATOM	339	N2	Y7	23	38.100	57.124	4.398	1.00	0.00	N
ATOM	340	C10	Y7	23	39.168	58.055	4.815	1.00	0.00	C
ATOM	341	C11	Y7	23	38.657	59.284	5.614	1.00	0.00	C
ATOM	342	C12	Y7	23	38.599	59.096	7.156	1.00	0.00	C
ATOM	343	C13	Y7	23	39.980	58.745	7.768	1.00	0.00	C
ATOM	344	C14	Y7	23	40.001	58.807	9.309	1.00	0.00	C
ATOM	345	N3	Y7	23	41.234	58.192	9.824	1.00	0.00	N
ATOM	346	O4	Y7	23	40.364	56.116	10.008	1.00	0.00	O
ATOM	347	C15	Y7	23	41.340	56.842	10.067	1.00	0.00	C
ATOM	348	C16	Y7	23	45.087	55.903	9.408	1.00	0.00	C
ATOM	349	C17	Y7	23	43.717	56.561	9.142	1.00	0.00	C
ATOM	350	C18	Y7	23	42.748	56.300	10.321	1.00	0.00	C
ATOM	351	C19	Y7	23	43.389	56.808	11.638	1.00	0.00	C
ATOM	352	C20	Y7	23	44.780	56.164	11.848	1.00	0.00	C
ATOM	353	N4	Y7	23	45.668	56.387	10.680	1.00	0.00	N
ATOM	354	C21	Y7	23	43.186	59.174	6.097	1.00	0.00	C
ATOM	355	C22	Y7	23	42.725	58.873	4.804	1.00	0.00	C
ATOM	356	C23	Y7	23	42.210	59.891	3.988	1.00	0.00	C
ATOM	357	C24	Y7	23	42.163	61.209	4.464	1.00	0.00	C
ATOM	358	C25	Y7	23	42.625	61.510	5.756	1.00	0.00	C
ATOM	359	C26	Y7	23	54.411	58.795	7.235	1.00	0.00	C
ATOM	360	C27	Y7	23	55.774	58.850	7.572	1.00	0.00	C
ATOM	361	C28	Y7	23	56.599	57.739	7.338	1.00	0.00	C
ATOM	362	C29	Y7	23	56.060	56.575	6.769	1.00	0.00	C
ATOM	363	C30	Y7	23	54.697	56.524	6.431	1.00	0.00	C
ATOM	364	C31	Y7	23	53.865	57.637	6.655	1.00	0.00	C
ATOM	365	C32	Y7	23	43.133	60.494	6.593	1.00	0.00	C
ATOM	366	N5	Y7	23	43.597	60.779	7.887	1.00	0.00	N
ATOM	367	N6	Y7	23	52.509	57.596	6.329	1.00	0.00	N
ATOM	368	C33	Y7	23	50.636	57.860	9.524	1.00	0.00	C
ATOM	369	C34	Y7	23	51.658	57.607	8.599	1.00	0.00	C
ATOM	370	C35	Y7	23	51.474	57.860	7.231	1.00	0.00	C
ATOM	371	C36	Y7	23	50.241	58.376	6.804	1.00	0.00	C
ATOM	372	C37	Y7	23	45.877	59.951	7.601	1.00	0.00	C
ATOM	373	C38	Y7	23	44.777	60.295	8.447	1.00	0.00	C
ATOM	374	C39	Y7	23	44.922	60.090	9.867	1.00	0.00	C
ATOM	375	C40	Y7	23	46.072	59.547	10.395	1.00	0.00	C
ATOM	376	C41	Y7	23	49.408	58.387	9.086	1.00	0.00	C
ATOM	377	C42	Y7	23	49.220	58.631	7.723	1.00	0.00	C
ATOM	378	O5	Y7	23	48.041	59.135	7.242	1.00	0.00	O

ATOM	379	C43	Y7	23	47.036	59.436	8.113	1.00	0.00	C
ATOM	380	C44	Y7	23	47.163	59.202	9.523	1.00	0.00	C
ATOM	381	C45	Y7	23	48.325	58.638	10.028	1.00	0.00	C
ATOM	382	C46	Y7	23	48.394	58.325	11.463	1.00	0.00	C
ATOM	383	C47	Y7	23	47.945	57.086	11.987	1.00	0.00	C
ATOM	384	C48	Y7	23	48.065	56.829	13.369	1.00	0.00	C
ATOM	385	C49	Y7	23	48.620	57.793	14.224	1.00	0.00	C
ATOM	386	C50	Y7	23	49.064	59.019	13.708	1.00	0.00	C
ATOM	387	C51	Y7	23	48.953	59.280	12.333	1.00	0.00	C
ATOM	388	S1	Y7	23	47.234	55.799	10.934	1.00	0.00	S
ATOM	389	O7	Y7	23	47.078	54.551	11.678	1.00	0.00	O
ATOM	390	O8	Y7	23	47.886	55.833	9.627	1.00	0.00	O
ATOM	391	H	Y7	23	37.157	49.940	5.870	1.00	0.00	H
ATOM	392	H1	Y7	23	38.937	51.112	4.491	1.00	0.00	H
ATOM	393	H2	Y7	23	36.511	51.928	4.893	1.00	0.00	H
ATOM	394	H3	Y7	23	37.180	53.014	6.130	1.00	0.00	H
ATOM	395	H6	Y7	23	43.316	62.458	9.171	1.00	0.00	H
ATOM	396	H7	Y7	23	41.913	62.083	8.152	1.00	0.00	H
ATOM	397	H8	Y7	23	42.271	61.052	9.537	1.00	0.00	H
ATOM	398	H9	Y7	23	51.878	58.176	4.380	1.00	0.00	H
ATOM	399	H10	Y7	23	53.032	56.830	4.403	1.00	0.00	H
ATOM	400	H11	Y7	23	51.353	56.538	4.887	1.00	0.00	H
ATOM	401	H12	Y7	23	35.598	56.352	2.913	1.00	0.00	H
ATOM	402	H13	Y7	23	36.864	55.421	2.050	1.00	0.00	H
ATOM	403	H14	Y7	23	35.908	54.851	4.741	1.00	0.00	H
ATOM	404	H15	Y7	23	39.932	57.504	5.374	1.00	0.00	H
ATOM	405	H16	Y7	23	39.667	58.431	3.911	1.00	0.00	H
ATOM	406	H17	Y7	23	39.326	60.128	5.402	1.00	0.00	H
ATOM	407	H18	Y7	23	37.670	59.583	5.237	1.00	0.00	H
ATOM	408	H19	Y7	23	38.233	60.028	7.605	1.00	0.00	H
ATOM	409	H20	Y7	23	37.867	58.320	7.413	1.00	0.00	H
ATOM	410	H21	Y7	23	40.264	57.731	7.461	1.00	0.00	H
ATOM	411	H22	Y7	23	40.739	59.429	7.374	1.00	0.00	H
ATOM	412	H23	Y7	23	39.943	59.853	9.640	1.00	0.00	H
ATOM	413	H24	Y7	23	39.128	58.279	9.715	1.00	0.00	H
ATOM	414	H25	Y7	23	42.082	58.730	9.877	1.00	0.00	H
ATOM	415	H26	Y7	23	45.744	56.145	8.562	1.00	0.00	H
ATOM	416	H27	Y7	23	44.972	54.810	9.439	1.00	0.00	H
ATOM	417	H28	Y7	23	43.863	57.636	9.001	1.00	0.00	H
ATOM	418	H29	Y7	23	43.295	56.166	8.209	1.00	0.00	H
ATOM	419	H30	Y7	23	42.633	55.215	10.410	1.00	0.00	H
ATOM	420	H31	Y7	23	43.494	57.900	11.619	1.00	0.00	H
ATOM	421	H32	Y7	23	42.742	56.556	12.489	1.00	0.00	H
ATOM	422	H33	Y7	23	45.230	56.585	12.756	1.00	0.00	H
ATOM	423	H34	Y7	23	44.639	55.087	12.018	1.00	0.00	H
ATOM	424	H35	Y7	23	43.588	58.373	6.710	1.00	0.00	H
ATOM	425	H36	Y7	23	42.781	57.854	4.429	1.00	0.00	H
ATOM	426	H37	Y7	23	41.865	59.664	2.981	1.00	0.00	H
ATOM	427	H38	Y7	23	41.783	62.003	3.824	1.00	0.00	H
ATOM	428	H39	Y7	23	42.609	62.545	6.088	1.00	0.00	H
ATOM	429	H40	Y7	23	53.777	59.659	7.411	1.00	0.00	H
ATOM	430	H41	Y7	23	56.191	59.754	8.011	1.00	0.00	H
ATOM	431	H42	Y7	23	57.655	57.780	7.597	1.00	0.00	H
ATOM	432	H43	Y7	23	56.698	55.711	6.593	1.00	0.00	H
ATOM	433	H44	Y7	23	54.284	55.613	6.007	1.00	0.00	H
ATOM	434	H45	Y7	23	50.802	57.635	10.576	1.00	0.00	H
ATOM	435	H46	Y7	23	52.601	57.192	8.944	1.00	0.00	H
ATOM	436	H47	Y7	23	50.059	58.603	5.762	1.00	0.00	H
ATOM	437	H48	Y7	23	45.804	60.111	6.532	1.00	0.00	H
ATOM	438	H49	Y7	23	44.117	60.341	10.544	1.00	0.00	H
ATOM	439	H50	Y7	23	46.136	59.380	11.467	1.00	0.00	H
ATOM	440	H51	Y7	23	47.739	55.874	13.783	1.00	0.00	H
ATOM	441	H52	Y7	23	48.717	57.585	15.287	1.00	0.00	H
ATOM	442	H53	Y7	23	49.503	59.762	14.371	1.00	0.00	H
ATOM	443	H54	Y7	23	49.310	60.227	11.936	1.00	0.00	H
ATOM	444	N	ALA	24	41.016	51.582	6.015	1.00	0.00	N
ATOM	445	H	ALA	24	41.052	50.692	5.543	1.00	0.00	H
ATOM	446	CA	ALA	24	42.285	52.149	6.491	1.00	0.00	C
ATOM	447	HA	ALA	24	43.022	51.346	6.452	1.00	0.00	H
ATOM	448	CB	ALA	24	42.738	53.226	5.492	1.00	0.00	C
ATOM	449	HB1	ALA	24	42.056	54.076	5.525	1.00	0.00	H
ATOM	450	HB2	ALA	24	43.743	53.567	5.747	1.00	0.00	H
ATOM	451	HB3	ALA	24	42.757	52.816	4.481	1.00	0.00	H
ATOM	452	C	ALA	24	42.287	52.641	7.965	1.00	0.00	C
ATOM	453	O	ALA	24	43.000	53.586	8.293	1.00	0.00	O
ATOM	454	N	LYS	25	41.493	52.034	8.865	1.00	0.00	N

```
ATOM    455  H    LYS    25      40.907  51.271   8.535  1.00  0.00           H
ATOM    456  CA   LYS    25      41.279  52.520  10.250  1.00  0.00           C
ATOM    457  HA   LYS    25      40.985  53.567  10.199  1.00  0.00           H
ATOM    458  CB   LYS    25      40.137  51.720  10.912  1.00  0.00           C
ATOM    459  HB2  LYS    25      40.316  50.654  10.760  1.00  0.00           H
ATOM    460  HB3  LYS    25      40.132  51.904  11.988  1.00  0.00           H
ATOM    461  CG   LYS    25      38.752  52.082  10.351  1.00  0.00           C
ATOM    462  HG2  LYS    25      38.800  52.093   9.265  1.00  0.00           H
ATOM    463  HG3  LYS    25      38.042  51.307  10.641  1.00  0.00           H
ATOM    464  CD   LYS    25      38.216  53.432  10.858  1.00  0.00           C
ATOM    465  HD2  LYS    25      37.550  53.246  11.702  1.00  0.00           H
ATOM    466  HD3  LYS    25      39.030  54.066  11.215  1.00  0.00           H
ATOM    467  CE   LYS    25      37.442  54.171   9.760  1.00  0.00           C
ATOM    468  HE2  LYS    25      36.724  53.485   9.301  1.00  0.00           H
ATOM    469  HE3  LYS    25      36.890  54.995  10.220  1.00  0.00           H
ATOM    470  NZ   LYS    25      38.371  54.710   8.739  1.00  0.00           N
ATOM    471  HZ1  LYS    25      38.867  53.974   8.244  1.00  0.00           H
ATOM    472  HZ2  LYS    25      37.906  55.255   8.019  1.00  0.00           H
ATOM    473  HZ3  LYS    25      39.078  55.300   9.169  1.00  0.00           H
ATOM    474  C    LYS    25      42.527  52.521  11.150  1.00  0.00           C
ATOM    475  O    LYS    25      42.544  53.262  12.128  1.00  0.00           O
ATOM    476  N    NME    26      43.559  51.738  10.819  1.00  0.00           N
ATOM    477  H    NME    26      43.462  51.180   9.989  1.00  0.00           H
ATOM    478  CH3  NME    26      44.828  51.685  11.546  1.00  0.00           C
ATOM    479 HH31  NME    26      45.420  52.575  11.326  1.00  0.00           H
ATOM    480 HH32  NME    26      44.644  51.648  12.622  1.00  0.00           H
ATOM    481 HH33  NME    26      45.396  50.801  11.253  1.00  0.00           H
TER     482       NME    26
END
```

Supplementary Table 16. The list of atoms and their coordination in the structure #114 of cR6G-P1-QSY7.

ATOM	1	H1	ACE	1	17.906	16.392	-6.411	1.00	0.00	H
ATOM	2	CH3	ACE	1	18.598	16.881	-5.727	1.00	0.00	C
ATOM	3	H2	ACE	1	19.245	17.555	-6.286	1.00	0.00	H
ATOM	4	H3	ACE	1	19.201	16.128	-5.223	1.00	0.00	H
ATOM	5	C	ACE	1	17.817	17.666	-4.705	1.00	0.00	C
ATOM	6	O	ACE	1	17.988	18.874	-4.578	1.00	0.00	O
ATOM	7	N	QS	2	16.953	16.954	-3.914	1.00	0.00	N
ATOM	8	C1	QS	2	15.972	17.577	-2.993	1.00	0.00	C
ATOM	9	C2	QS	2	16.630	18.621	-2.054	1.00	0.00	C
ATOM	10	C3	QS	2	15.138	16.505	-2.236	1.00	0.00	C
ATOM	11	O1	QS	2	16.364	19.808	-2.126	1.00	0.00	O
ATOM	12	S	QS	2	16.171	15.335	-1.230	1.00	0.00	S
ATOM	13	C4	QS	2	15.333	4.547	-4.000	1.00	0.00	C
ATOM	14	C5	QS	2	15.325	5.760	-4.646	1.00	0.00	C
ATOM	15	C6	QS	2	15.905	6.936	-4.047	1.00	0.00	C
ATOM	16	C7	QS	2	16.510	6.785	-2.739	1.00	0.00	C
ATOM	17	C8	QS	2	16.562	5.567	-2.070	1.00	0.00	C
ATOM	18	C9	QS	2	15.925	4.414	-2.697	1.00	0.00	C
ATOM	19	C10	QS	2	15.889	8.132	-4.726	1.00	0.00	C
ATOM	20	N2	QS	2	16.443	9.318	-4.234	1.00	0.00	N
ATOM	21	O2	QS	2	15.386	8.248	-5.835	1.00	0.00	O
ATOM	22	C11	QS	2	16.724	10.492	-5.075	1.00	0.00	C
ATOM	23	C12	QS	2	17.625	11.516	-4.346	1.00	0.00	C
ATOM	24	N3	QS	2	16.951	12.161	-3.193	1.00	0.00	N
ATOM	25	C13	QS	2	16.371	13.392	-3.253	1.00	0.00	C
ATOM	26	C14	QS	2	15.668	13.691	-1.930	1.00	0.00	C
ATOM	27	C15	QS	2	16.103	12.506	-1.021	1.00	0.00	C
ATOM	28	C16	QS	2	16.792	11.555	-1.981	1.00	0.00	C
ATOM	29	O3	QS	2	17.116	10.413	-1.732	1.00	0.00	O
ATOM	30	O4	QS	2	16.436	14.153	-4.201	1.00	0.00	O
ATOM	31	C17	QS	2	15.835	3.169	-2.075	1.00	0.00	C
ATOM	32	C18	QS	2	16.156	1.971	-2.860	1.00	0.00	C
ATOM	33	C19	QS	2	15.714	0.733	-2.377	1.00	0.00	C
ATOM	34	O5	QS	2	14.997	0.652	-1.213	1.00	0.00	O
ATOM	35	C20	QS	2	14.598	1.751	-0.497	1.00	0.00	C
ATOM	36	C21	QS	2	14.960	3.047	-0.896	1.00	0.00	C
ATOM	37	C22	QS	2	13.778	1.541	0.611	1.00	0.00	C
ATOM	38	C23	QS	2	13.279	2.621	1.341	1.00	0.00	C
ATOM	39	C24	QS	2	13.590	3.935	0.934	1.00	0.00	C
ATOM	40	C25	QS	2	14.416	4.142	-0.188	1.00	0.00	C
ATOM	41	C26	QS	2	16.990	1.996	-3.998	1.00	0.00	C
ATOM	42	C27	QS	2	17.301	0.806	-4.684	1.00	0.00	C
ATOM	43	C28	QS	2	16.801	-0.427	-4.212	1.00	0.00	C
ATOM	44	C29	QS	2	16.020	-0.449	-3.050	1.00	0.00	C
ATOM	45	C30	QS	2	18.194	0.877	-5.926	1.00	0.00	C
ATOM	46	C31	QS	2	13.039	5.148	1.687	1.00	0.00	C
ATOM	47	N4	QS	2	12.465	2.413	2.445	1.00	0.00	N
ATOM	48	N5	QS	2	17.116	-1.598	-4.892	1.00	0.00	N
ATOM	49	C32	QS	2	12.074	1.103	2.984	1.00	0.00	C
ATOM	50	C33	QS	2	11.197	1.292	4.234	1.00	0.00	C
ATOM	51	C34	QS	2	16.639	-2.953	-4.570	1.00	0.00	C
ATOM	52	C35	QS	2	17.552	-3.600	-3.512	1.00	0.00	C
ATOM	53	C36	QS	2	17.334	5.538	-0.784	1.00	0.00	C
ATOM	54	O7	QS	2	17.797	6.516	-0.239	1.00	0.00	O
ATOM	55	O8	QS	2	17.612	4.343	-0.272	1.00	0.00	O
ATOM	56	H1	QS	2	16.855	15.958	-4.112	1.00	0.00	H
ATOM	57	H2	QS	2	15.272	18.160	-3.607	1.00	0.00	H
ATOM	58	H3	QS	2	14.426	17.006	-1.565	1.00	0.00	H
ATOM	59	H4	QS	2	14.552	15.932	-2.966	1.00	0.00	H
ATOM	60	H6	QS	2	14.848	3.695	-4.467	1.00	0.00	H
ATOM	61	H7	QS	2	14.861	5.837	-5.627	1.00	0.00	H
ATOM	62	H8	QS	2	16.979	7.652	-2.278	1.00	0.00	H
ATOM	63	H9	QS	2	16.718	9.354	-3.254	1.00	0.00	H
ATOM	64	H10	QS	2	17.228	10.157	-5.993	1.00	0.00	H
ATOM	65	H11	QS	2	15.777	10.965	-5.370	1.00	0.00	H
ATOM	66	H12	QS	2	18.535	11.009	-3.997	1.00	0.00	H
ATOM	67	H13	QS	2	17.937	12.286	-5.065	1.00	0.00	H
ATOM	68	H14	QS	2	14.588	13.650	-2.118	1.00	0.00	H
ATOM	69	H15	QS	2	15.252	12.003	-0.546	1.00	0.00	H
ATOM	70	H16	QS	2	16.828	12.810	-0.257	1.00	0.00	H
ATOM	71	H17	QS	2	13.533	0.521	0.877	1.00	0.00	H
ATOM	72	H18	QS	2	14.617	5.162	-0.498	1.00	0.00	H

ATOM	73	H19	QS	2	17.414	2.935	-4.347	1.00	0.00	H
ATOM	74	H20	QS	2	15.651	-1.379	-2.638	1.00	0.00	H
ATOM	75	H21	QS	2	17.675	0.482	-6.809	1.00	0.00	H
ATOM	76	H22	QS	2	18.491	1.907	-6.158	1.00	0.00	H
ATOM	77	H23	QS	2	19.115	0.298	-5.783	1.00	0.00	H
ATOM	78	H24	QS	2	13.364	5.142	2.736	1.00	0.00	H
ATOM	79	H25	QS	2	13.381	6.093	1.247	1.00	0.00	H
ATOM	80	H26	QS	2	11.942	5.159	1.669	1.00	0.00	H
ATOM	81	H27	QS	2	12.127	3.237	2.921	1.00	0.00	H
ATOM	82	H28	QS	2	17.712	-1.497	-5.702	1.00	0.00	H
ATOM	83	H29	QS	2	11.511	0.535	2.230	1.00	0.00	H
ATOM	84	H30	QS	2	12.967	0.522	3.254	1.00	0.00	H
ATOM	85	H31	QS	2	11.742	1.836	5.016	1.00	0.00	H
ATOM	86	H32	QS	2	10.283	1.848	3.991	1.00	0.00	H
ATOM	87	H33	QS	2	10.902	0.317	4.642	1.00	0.00	H
ATOM	88	H34	QS	2	15.602	-2.932	-4.210	1.00	0.00	H
ATOM	89	H35	QS	2	16.653	-3.574	-5.477	1.00	0.00	H
ATOM	90	H36	QS	2	17.205	-4.614	-3.275	1.00	0.00	H
ATOM	91	H37	QS	2	18.583	-3.671	-3.878	1.00	0.00	H
ATOM	92	H38	QS	2	17.556	-3.016	-2.584	1.00	0.00	H
ATOM	93	H40	QS	2	18.164	4.624	0.477	1.00	0.00	H
ATOM	94	N	ALA	3	17.611	18.165	-1.201	1.00	0.00	N
ATOM	95	H	ALA	3	17.795	17.173	-1.190	1.00	0.00	H
ATOM	96	CA	ALA	3	18.313	18.991	-0.215	1.00	0.00	C
ATOM	97	HA	ALA	3	17.624	19.759	0.141	1.00	0.00	H
ATOM	98	CB	ALA	3	18.665	18.091	0.977	1.00	0.00	C
ATOM	99	HB1	ALA	3	17.764	17.613	1.366	1.00	0.00	H
ATOM	100	HB2	ALA	3	19.380	17.325	0.673	1.00	0.00	H
ATOM	101	HB3	ALA	3	19.110	18.691	1.773	1.00	0.00	H
ATOM	102	C	ALA	3	19.551	19.739	-0.768	1.00	0.00	C
ATOM	103	O	ALA	3	20.315	20.313	0.004	1.00	0.00	O
ATOM	104	N	ALA	4	19.791	19.727	-2.087	1.00	0.00	N
ATOM	105	H	ALA	4	19.128	19.274	-2.698	1.00	0.00	H
ATOM	106	CA	ALA	4	20.949	20.401	-2.683	1.00	0.00	C
ATOM	107	HA	ALA	4	21.841	20.048	-2.161	1.00	0.00	H
ATOM	108	CB	ALA	4	21.070	19.984	-4.154	1.00	0.00	C
ATOM	109	HB1	ALA	4	21.113	18.896	-4.228	1.00	0.00	H
ATOM	110	HB2	ALA	4	20.214	20.347	-4.719	1.00	0.00	H
ATOM	111	HB3	ALA	4	21.982	20.398	-4.584	1.00	0.00	H
ATOM	112	C	ALA	4	20.896	21.936	-2.515	1.00	0.00	C
ATOM	113	O	ALA	4	19.826	22.525	-2.363	1.00	0.00	O
ATOM	114	N	ALA	5	22.055	22.599	-2.615	1.00	0.00	N
ATOM	115	H	ALA	5	22.884	22.046	-2.768	1.00	0.00	H
ATOM	116	CA	ALA	5	22.285	24.017	-2.288	1.00	0.00	C
ATOM	117	HA	ALA	5	21.885	24.178	-1.285	1.00	0.00	H
ATOM	118	CB	ALA	5	23.805	24.227	-2.206	1.00	0.00	C
ATOM	119	HB1	ALA	5	24.255	24.107	-3.192	1.00	0.00	H
ATOM	120	HB2	ALA	5	24.022	25.232	-1.840	1.00	0.00	H
ATOM	121	HB3	ALA	5	24.248	23.509	-1.514	1.00	0.00	H
ATOM	122	C	ALA	5	21.592	25.077	-3.191	1.00	0.00	C
ATOM	123	O	ALA	5	22.036	26.222	-3.274	1.00	0.00	O
ATOM	124	N	HIP	6	20.483	24.739	-3.858	1.00	0.00	N
ATOM	125	H	HIP	6	20.124	23.809	-3.669	1.00	0.00	H
ATOM	126	CA	HIP	6	19.614	25.658	-4.613	1.00	0.00	C
ATOM	127	HA	HIP	6	20.246	26.300	-5.230	1.00	0.00	H
ATOM	128	CB	HIP	6	18.716	24.833	-5.566	1.00	0.00	C
ATOM	129	HB2	HIP	6	17.974	25.490	-6.023	1.00	0.00	H
ATOM	130	HB3	HIP	6	19.341	24.447	-6.372	1.00	0.00	H
ATOM	131	CG	HIP	6	18.002	23.664	-4.918	1.00	0.00	C
ATOM	132	ND1	HIP	6	17.173	23.733	-3.814	1.00	0.00	N
ATOM	133	HD1	HIP	6	16.937	24.599	-3.329	1.00	0.00	H
ATOM	134	CE1	HIP	6	16.834	22.477	-3.462	1.00	0.00	C
ATOM	135	HE1	HIP	6	16.261	22.186	-2.584	1.00	0.00	H
ATOM	136	NE2	HIP	6	17.404	21.618	-4.330	1.00	0.00	N
ATOM	137	HE2	HIP	6	17.381	20.594	-4.253	1.00	0.00	H
ATOM	138	CD2	HIP	6	18.137	22.344	-5.252	1.00	0.00	C
ATOM	139	HD2	HIP	6	18.743	21.939	-6.054	1.00	0.00	H
ATOM	140	C	HIP	6	18.833	26.601	-3.669	1.00	0.00	C
ATOM	141	O	HIP	6	17.619	26.471	-3.504	1.00	0.00	O
ATOM	142	N	ALA	7	19.540	27.504	-2.990	1.00	0.00	N
ATOM	143	H	ALA	7	20.546	27.495	-3.117	1.00	0.00	H
ATOM	144	CA	ALA	7	18.979	28.456	-2.030	1.00	0.00	C
ATOM	145	HA	ALA	7	18.250	27.929	-1.412	1.00	0.00	H
ATOM	146	CB	ALA	7	20.110	28.942	-1.115	1.00	0.00	C
ATOM	147	HB1	ALA	7	20.864	29.473	-1.699	1.00	0.00	H
ATOM	148	HB2	ALA	7	19.712	29.615	-0.354	1.00	0.00	H

ATOM	149	HB3	ALA	7	20.579	28.091	-0.618	1.00	0.00	H
ATOM	150	C	ALA	7	18.260	29.640	-2.709	1.00	0.00	C
ATOM	151	O	ALA	7	18.687	30.130	-3.759	1.00	0.00	O
ATOM	152	N	ALA	8	17.194	30.144	-2.079	1.00	0.00	N
ATOM	153	H	ALA	8	16.858	29.674	-1.252	1.00	0.00	H
ATOM	154	CA	ALA	8	16.450	31.319	-2.546	1.00	0.00	C
ATOM	155	HA	ALA	8	16.383	31.290	-3.635	1.00	0.00	H
ATOM	156	CB	ALA	8	15.025	31.228	-1.986	1.00	0.00	C
ATOM	157	HB1	ALA	8	15.039	31.257	-0.896	1.00	0.00	H
ATOM	158	HB2	ALA	8	14.433	32.068	-2.354	1.00	0.00	H
ATOM	159	HB3	ALA	8	14.552	30.301	-2.317	1.00	0.00	H
ATOM	160	C	ALA	8	17.109	32.663	-2.161	1.00	0.00	C
ATOM	161	O	ALA	8	17.075	33.611	-2.943	1.00	0.00	O
ATOM	162	N	ALA	9	17.715	32.753	-0.973	1.00	0.00	N
ATOM	163	H	ALA	9	17.740	31.941	-0.380	1.00	0.00	H
ATOM	164	CA	ALA	9	18.262	33.998	-0.431	1.00	0.00	C
ATOM	165	HA	ALA	9	17.521	34.787	-0.566	1.00	0.00	H
ATOM	166	CB	ALA	9	18.467	33.813	1.079	1.00	0.00	C
ATOM	167	HB1	ALA	9	18.844	34.740	1.514	1.00	0.00	H
ATOM	168	HB2	ALA	9	17.520	33.561	1.558	1.00	0.00	H
ATOM	169	HB3	ALA	9	19.191	33.018	1.268	1.00	0.00	H
ATOM	170	C	ALA	9	19.562	34.434	-1.138	1.00	0.00	C
ATOM	171	O	ALA	9	20.610	33.815	-0.964	1.00	0.00	O
ATOM	172	N	HIP	10	19.485	35.502	-1.943	1.00	0.00	N
ATOM	173	H	HIP	10	18.560	35.873	-2.115	1.00	0.00	H
ATOM	174	CA	HIP	10	20.622	36.219	-2.558	1.00	0.00	C
ATOM	175	HA	HIP	10	21.307	36.538	-1.770	1.00	0.00	H
ATOM	176	CB	HIP	10	21.398	35.305	-3.534	1.00	0.00	C
ATOM	177	HB2	HIP	10	21.990	35.910	-4.223	1.00	0.00	H
ATOM	178	HB3	HIP	10	22.104	34.709	-2.952	1.00	0.00	H
ATOM	179	CG	HIP	10	20.526	34.358	-4.326	1.00	0.00	C
ATOM	180	ND1	HIP	10	19.416	34.703	-5.076	1.00	0.00	N
ATOM	181	HD1	HIP	10	19.053	35.655	-5.154	1.00	0.00	H
ATOM	182	CE1	HIP	10	18.780	33.565	-5.423	1.00	0.00	C
ATOM	183	HE1	HIP	10	17.799	33.513	-5.886	1.00	0.00	H
ATOM	184	NE2	HIP	10	19.467	32.514	-4.924	1.00	0.00	N
ATOM	185	HE2	HIP	10	19.139	31.544	-4.874	1.00	0.00	H
ATOM	186	CD2	HIP	10	20.558	32.996	-4.227	1.00	0.00	C
ATOM	187	HD2	HIP	10	21.242	32.421	-3.612	1.00	0.00	H
ATOM	188	C	HIP	10	20.122	37.469	-3.296	1.00	0.00	C
ATOM	189	O	HIP	10	19.204	37.358	-4.110	1.00	0.00	O
ATOM	190	N	ALA	11	20.739	38.622	-3.041	1.00	0.00	N
ATOM	191	H	ALA	11	21.532	38.619	-2.413	1.00	0.00	H
ATOM	192	CA	ALA	11	20.416	39.926	-3.630	1.00	0.00	C
ATOM	193	HA	ALA	11	20.136	39.797	-4.677	1.00	0.00	H
ATOM	194	CB	ALA	11	19.221	40.522	-2.867	1.00	0.00	C
ATOM	195	HB1	ALA	11	19.483	40.673	-1.819	1.00	0.00	H
ATOM	196	HB2	ALA	11	18.936	41.480	-3.303	1.00	0.00	H
ATOM	197	HB3	ALA	11	18.365	39.849	-2.929	1.00	0.00	H
ATOM	198	C	ALA	11	21.648	40.856	-3.572	1.00	0.00	C
ATOM	199	O	ALA	11	22.671	40.477	-3.003	1.00	0.00	O
ATOM	200	N	ALA	12	21.553	42.065	-4.136	1.00	0.00	N
ATOM	201	H	ALA	12	20.675	42.348	-4.545	1.00	0.00	H
ATOM	202	CA	ALA	12	22.623	43.067	-4.134	1.00	0.00	C
ATOM	203	HA	ALA	12	23.281	42.883	-3.282	1.00	0.00	H
ATOM	204	CB	ALA	12	23.452	42.912	-5.417	1.00	0.00	C
ATOM	205	HB1	ALA	12	24.274	43.629	-5.418	1.00	0.00	H
ATOM	206	HB2	ALA	12	23.867	41.904	-5.470	1.00	0.00	H
ATOM	207	HB3	ALA	12	22.824	43.090	-6.291	1.00	0.00	H
ATOM	208	C	ALA	12	22.063	44.495	-3.984	1.00	0.00	C
ATOM	209	O	ALA	12	20.906	44.754	-4.315	1.00	0.00	O
ATOM	210	N	ALA	13	22.880	45.424	-3.480	1.00	0.00	N
ATOM	211	H	ALA	13	23.827	45.165	-3.256	1.00	0.00	H
ATOM	212	CA	ALA	13	22.485	46.819	-3.280	1.00	0.00	C
ATOM	213	HA	ALA	13	21.504	46.828	-2.801	1.00	0.00	H
ATOM	214	CB	ALA	13	23.485	47.482	-2.325	1.00	0.00	C
ATOM	215	HB1	ALA	13	23.517	46.933	-1.382	1.00	0.00	H
ATOM	216	HB2	ALA	13	24.481	47.489	-2.771	1.00	0.00	H
ATOM	217	HB3	ALA	13	23.178	48.509	-2.121	1.00	0.00	H
ATOM	218	C	ALA	13	22.371	47.598	-4.608	1.00	0.00	C
ATOM	219	O	ALA	13	23.193	47.440	-5.510	1.00	0.00	O
ATOM	220	N	ALA	14	21.393	48.505	-4.705	1.00	0.00	N
ATOM	221	H	ALA	14	20.746	48.577	-3.934	1.00	0.00	H
ATOM	222	CA	ALA	14	21.087	49.303	-5.900	1.00	0.00	C
ATOM	223	HA	ALA	14	21.130	48.633	-6.762	1.00	0.00	H
ATOM	224	CB	ALA	14	19.636	49.793	-5.777	1.00	0.00	C

ATOM	225	HB1	ALA	14	18.965	48.943	-5.642	1.00	0.00	H
ATOM	226	HB2	ALA	14	19.537	50.470	-4.927	1.00	0.00	H
ATOM	227	HB3	ALA	14	19.347	50.322	-6.686	1.00	0.00	H
ATOM	228	C	ALA	14	22.097	50.448	-6.182	1.00	0.00	C
ATOM	229	O	ALA	14	21.721	51.614	-6.274	1.00	0.00	O
ATOM	230	N	HIP	15	23.394	50.123	-6.281	1.00	0.00	N
ATOM	231	H	HIP	15	23.592	49.132	-6.157	1.00	0.00	H
ATOM	232	CA	HIP	15	24.552	50.980	-6.628	1.00	0.00	C
ATOM	233	HA	HIP	15	25.435	50.378	-6.404	1.00	0.00	H
ATOM	234	CB	HIP	15	24.601	51.241	-8.150	1.00	0.00	C
ATOM	235	HB2	HIP	15	25.575	51.667	-8.399	1.00	0.00	H
ATOM	236	HB3	HIP	15	24.540	50.282	-8.669	1.00	0.00	H
ATOM	237	CG	HIP	15	23.537	52.167	-8.707	1.00	0.00	C
ATOM	238	ND1	HIP	15	23.267	53.466	-8.304	1.00	0.00	N
ATOM	239	HD1	HIP	15	23.701	53.972	-7.527	1.00	0.00	H
ATOM	240	CE1	HIP	15	22.253	53.938	-9.053	1.00	0.00	C
ATOM	241	HE1	HIP	15	21.804	54.922	-8.941	1.00	0.00	H
ATOM	242	NE2	HIP	15	21.882	52.985	-9.934	1.00	0.00	N
ATOM	243	HE2	HIP	15	21.140	53.066	-10.623	1.00	0.00	H
ATOM	244	CD2	HIP	15	22.679	51.870	-9.731	1.00	0.00	C
ATOM	245	HD2	HIP	15	22.638	50.934	-10.278	1.00	0.00	H
ATOM	246	C	HIP	15	24.789	52.272	-5.818	1.00	0.00	C
ATOM	247	O	HIP	15	25.770	52.954	-6.095	1.00	0.00	O
ATOM	248	N	ALA	16	23.926	52.648	-4.870	1.00	0.00	N
ATOM	249	H	ALA	16	23.073	52.114	-4.796	1.00	0.00	H
ATOM	250	CA	ALA	16	24.006	53.912	-4.122	1.00	0.00	C
ATOM	251	HA	ALA	16	23.050	54.055	-3.616	1.00	0.00	H
ATOM	252	CB	ALA	16	25.076	53.766	-3.023	1.00	0.00	C
ATOM	253	HB1	ALA	16	24.876	52.874	-2.429	1.00	0.00	H
ATOM	254	HB2	ALA	16	26.069	53.676	-3.469	1.00	0.00	H
ATOM	255	HB3	ALA	16	25.055	54.630	-2.358	1.00	0.00	H
ATOM	256	C	ALA	16	24.180	55.135	-5.062	1.00	0.00	C
ATOM	257	O	ALA	16	23.548	55.180	-6.119	1.00	0.00	O
ATOM	258	N	ALA	17	25.022	56.111	-4.694	1.00	0.00	N
ATOM	259	H	ALA	17	25.544	55.971	-3.844	1.00	0.00	H
ATOM	260	CA	ALA	17	25.392	57.275	-5.519	1.00	0.00	C
ATOM	261	HA	ALA	17	26.008	57.912	-4.884	1.00	0.00	H
ATOM	262	CB	ALA	17	26.303	56.813	-6.669	1.00	0.00	C
ATOM	263	HB1	ALA	17	25.736	56.214	-7.381	1.00	0.00	H
ATOM	264	HB2	ALA	17	26.711	57.682	-7.188	1.00	0.00	H
ATOM	265	HB3	ALA	17	27.130	56.218	-6.278	1.00	0.00	H
ATOM	266	C	ALA	17	24.217	58.183	-5.961	1.00	0.00	C
ATOM	267	O	ALA	17	24.259	58.801	-7.022	1.00	0.00	O
ATOM	268	N	ALA	18	23.161	58.275	-5.146	1.00	0.00	N
ATOM	269	H	ALA	18	23.173	57.750	-4.289	1.00	0.00	H
ATOM	270	CA	ALA	18	22.035	59.180	-5.382	1.00	0.00	C
ATOM	271	HA	ALA	18	21.682	59.027	-6.404	1.00	0.00	H
ATOM	272	CB	ALA	18	20.900	58.803	-4.421	1.00	0.00	C
ATOM	273	HB1	ALA	18	20.023	59.420	-4.624	1.00	0.00	H
ATOM	274	HB2	ALA	18	20.627	57.756	-4.563	1.00	0.00	H
ATOM	275	HB3	ALA	18	21.214	58.959	-3.388	1.00	0.00	H
ATOM	276	C	ALA	18	22.424	60.670	-5.245	1.00	0.00	C
ATOM	277	O	ALA	18	23.377	61.026	-4.548	1.00	0.00	O
ATOM	278	N	HIE	19	21.629	61.556	-5.854	1.00	0.00	N
ATOM	279	H	HIE	19	20.873	61.193	-6.429	1.00	0.00	H
ATOM	280	CA	HIE	19	21.871	63.005	-5.974	1.00	0.00	C
ATOM	281	HA	HIE	19	22.803	63.144	-6.516	1.00	0.00	H
ATOM	282	CB	HIE	19	20.737	63.628	-6.808	1.00	0.00	C
ATOM	283	HB2	HIE	19	19.831	63.662	-6.201	1.00	0.00	H
ATOM	284	HB3	HIE	19	21.005	64.655	-7.061	1.00	0.00	H
ATOM	285	CG	HIE	19	20.408	62.880	-8.079	1.00	0.00	C
ATOM	286	ND1	HIE	19	19.776	61.634	-8.133	1.00	0.00	N
ATOM	287	CE1	HIE	19	19.652	61.342	-9.437	1.00	0.00	C
ATOM	288	HE1	HIE	19	19.184	60.446	-9.825	1.00	0.00	H
ATOM	289	NE2	HIE	19	20.181	62.321	-10.190	1.00	0.00	N
ATOM	290	HE2	HIE	19	20.204	62.339	-11.201	1.00	0.00	H
ATOM	291	CD2	HIE	19	20.663	63.301	-9.351	1.00	0.00	C
ATOM	292	HD2	HIE	19	21.143	64.224	-9.642	1.00	0.00	H
ATOM	293	C	HIE	19	22.015	63.760	-4.640	1.00	0.00	C
ATOM	294	O	HIE	19	22.539	64.872	-4.620	1.00	0.00	O
ATOM	295	N	ALA	20	21.600	63.151	-3.526	1.00	0.00	N
ATOM	296	H	ALA	20	21.179	62.241	-3.625	1.00	0.00	H
ATOM	297	CA	ALA	20	21.829	63.647	-2.169	1.00	0.00	C
ATOM	298	HA	ALA	20	21.410	64.651	-2.091	1.00	0.00	H
ATOM	299	CB	ALA	20	21.069	62.732	-1.201	1.00	0.00	C
ATOM	300	HB1	ALA	20	20.007	62.722	-1.452	1.00	0.00	H

ATOM	301	HB2	ALA	20	21.465	61.717	-1.252	1.00	0.00	H
ATOM	302	HB3	ALA	20	21.183	63.105	-0.182	1.00	0.00	H
ATOM	303	C	ALA	20	23.323	63.745	-1.785	1.00	0.00	C
ATOM	304	O	ALA	20	23.660	64.517	-0.891	1.00	0.00	O
ATOM	305	N	ALA	21	24.223	63.025	-2.471	1.00	0.00	N
ATOM	306	H	ALA	21	23.891	62.401	-3.198	1.00	0.00	H
ATOM	307	CA	ALA	21	25.673	63.166	-2.304	1.00	0.00	C
ATOM	308	HA	ALA	21	25.929	62.849	-1.291	1.00	0.00	H
ATOM	309	CB	ALA	21	26.372	62.230	-3.298	1.00	0.00	C
ATOM	310	HB1	ALA	21	26.126	62.523	-4.320	1.00	0.00	H
ATOM	311	HB2	ALA	21	27.452	62.281	-3.162	1.00	0.00	H
ATOM	312	HB3	ALA	21	26.042	61.203	-3.134	1.00	0.00	H
ATOM	313	C	ALA	21	26.136	64.630	-2.476	1.00	0.00	C
ATOM	314	O	ALA	21	25.505	65.390	-3.209	1.00	0.00	O
ATOM	315	N	ALA	22	27.230	65.015	-1.802	1.00	0.00	N
ATOM	316	H	ALA	22	27.701	64.312	-1.257	1.00	0.00	H
ATOM	317	CA	ALA	22	27.601	66.409	-1.511	1.00	0.00	C
ATOM	318	HA	ALA	22	26.947	66.754	-0.707	1.00	0.00	H
ATOM	319	CB	ALA	22	29.033	66.437	-0.962	1.00	0.00	C
ATOM	320	HB1	ALA	22	29.311	67.464	-0.717	1.00	0.00	H
ATOM	321	HB2	ALA	22	29.095	65.835	-0.054	1.00	0.00	H
ATOM	322	HB3	ALA	22	29.732	66.046	-1.698	1.00	0.00	H
ATOM	323	C	ALA	22	27.389	67.401	-2.674	1.00	0.00	C
ATOM	324	O	ALA	22	26.437	68.181	-2.627	1.00	0.00	O
ATOM	325	N	Y7	23	28.232	67.354	-3.767	1.00	0.00	N
ATOM	326	C1	Y7	23	28.067	68.246	-4.940	1.00	0.00	C
ATOM	327	C2	Y7	23	27.992	69.741	-4.501	1.00	0.00	C
ATOM	328	C3	Y7	23	26.846	67.762	-5.767	1.00	0.00	C
ATOM	329	O1	Y7	23	28.919	70.294	-3.935	1.00	0.00	O
ATOM	330	S	Y7	23	27.062	66.065	-6.458	1.00	0.00	S
ATOM	331	C4	Y7	23	39.348	71.551	-10.960	1.00	0.00	C
ATOM	332	C5	Y7	23	30.455	67.816	-8.156	1.00	0.00	C
ATOM	333	O2	Y7	23	23.889	67.349	-6.079	1.00	0.00	O
ATOM	334	O3	Y7	23	23.681	64.595	-9.661	1.00	0.00	O
ATOM	335	C6	Y7	23	24.088	65.046	-8.611	1.00	0.00	C
ATOM	336	C7	Y7	23	25.198	64.442	-7.772	1.00	0.00	C
ATOM	337	C8	Y7	23	25.318	65.403	-6.556	1.00	0.00	C
ATOM	338	C9	Y7	23	24.213	66.441	-6.822	1.00	0.00	C
ATOM	339	N2	Y7	23	23.595	66.160	-8.001	1.00	0.00	N
ATOM	340	C10	Y7	23	22.550	67.019	-8.603	1.00	0.00	C
ATOM	341	C11	Y7	23	23.146	68.272	-9.297	1.00	0.00	C
ATOM	342	C12	Y7	23	22.978	69.572	-8.459	1.00	0.00	C
ATOM	343	C13	Y7	23	24.043	70.652	-8.775	1.00	0.00	C
ATOM	344	C14	Y7	23	25.411	70.294	-8.160	1.00	0.00	C
ATOM	345	N3	Y7	23	26.431	71.319	-8.406	1.00	0.00	N
ATOM	346	O4	Y7	23	28.171	70.043	-7.728	1.00	0.00	O
ATOM	347	C15	Y7	23	27.772	71.101	-8.180	1.00	0.00	C
ATOM	348	C16	Y7	23	30.144	74.194	-7.726	1.00	0.00	C
ATOM	349	C17	Y7	23	29.098	73.113	-7.370	1.00	0.00	C
ATOM	350	C18	Y7	23	28.709	72.244	-8.596	1.00	0.00	C
ATOM	351	C19	Y7	23	29.988	71.720	-9.302	1.00	0.00	C
ATOM	352	C20	Y7	23	30.981	72.856	-9.622	1.00	0.00	C
ATOM	353	N4	Y7	23	31.323	73.612	-8.401	1.00	0.00	N
ATOM	354	C21	Y7	23	40.637	74.289	-10.911	1.00	0.00	C
ATOM	355	C22	Y7	23	41.694	75.203	-10.765	1.00	0.00	C
ATOM	356	C23	Y7	23	42.324	75.357	-9.521	1.00	0.00	C
ATOM	357	C24	Y7	23	41.895	74.598	-8.421	1.00	0.00	C
ATOM	358	C25	Y7	23	40.837	73.685	-8.566	1.00	0.00	C
ATOM	359	C26	Y7	23	31.907	67.298	-4.691	1.00	0.00	C
ATOM	360	C27	Y7	23	31.897	66.352	-3.653	1.00	0.00	C
ATOM	361	C28	Y7	23	31.232	65.129	-3.821	1.00	0.00	C
ATOM	362	C29	Y7	23	30.568	64.861	-5.026	1.00	0.00	C
ATOM	363	C30	Y7	23	30.583	65.806	-6.065	1.00	0.00	C
ATOM	364	C31	Y7	23	31.256	67.040	-5.916	1.00	0.00	C
ATOM	365	C32	Y7	23	40.203	73.520	-9.813	1.00	0.00	C
ATOM	366	N5	Y7	23	39.147	72.615	-9.948	1.00	0.00	N
ATOM	367	N6	Y7	23	31.293	67.994	-6.949	1.00	0.00	N
ATOM	368	C33	Y7	23	32.367	71.465	-6.241	1.00	0.00	C
ATOM	369	C34	Y7	23	31.613	70.283	-6.235	1.00	0.00	C
ATOM	370	C35	Y7	23	32.052	69.156	-6.936	1.00	0.00	C
ATOM	371	C36	Y7	23	33.252	69.213	-7.642	1.00	0.00	C
ATOM	372	C37	Y7	23	37.117	71.469	-9.110	1.00	0.00	C
ATOM	373	C38	Y7	23	37.968	72.622	-9.206	1.00	0.00	C
ATOM	374	C39	Y7	23	37.569	73.814	-8.512	1.00	0.00	C
ATOM	375	C40	Y7	23	36.405	73.870	-7.782	1.00	0.00	C
ATOM	376	C41	Y7	23	33.578	71.519	-6.956	1.00	0.00	C

ATOM	377	C42	Y7	23	34.013	70.385	-7.652	1.00	0.00	C
ATOM	378	O5	Y7	23	35.189	70.378	-8.350	1.00	0.00	O
ATOM	379	C43	Y7	23	35.954	71.508	-8.387	1.00	0.00	C
ATOM	380	C44	Y7	23	35.565	72.703	-7.692	1.00	0.00	C
ATOM	381	C45	Y7	23	34.366	72.743	-6.995	1.00	0.00	C
ATOM	382	C46	Y7	23	33.982	74.003	-6.339	1.00	0.00	C
ATOM	383	C47	Y7	23	33.188	74.982	-6.988	1.00	0.00	C
ATOM	384	C48	Y7	23	32.879	76.186	-6.320	1.00	0.00	C
ATOM	385	C49	Y7	23	33.345	76.412	-5.016	1.00	0.00	C
ATOM	386	C50	Y7	23	34.120	75.440	-4.368	1.00	0.00	C
ATOM	387	C51	Y7	23	34.436	74.243	-5.029	1.00	0.00	C
ATOM	388	S1	Y7	23	32.551	74.742	-8.663	1.00	0.00	S
ATOM	389	O7	Y7	23	33.558	74.049	-9.463	1.00	0.00	O
ATOM	390	O8	Y7	23	31.878	75.949	-9.137	1.00	0.00	O
ATOM	391	H	Y7	23	29.013	66.712	-3.769	1.00	0.00	H
ATOM	392	H1	Y7	23	28.955	68.181	-5.575	1.00	0.00	H
ATOM	393	H2	Y7	23	25.977	67.770	-5.103	1.00	0.00	H
ATOM	394	H3	Y7	23	26.660	68.440	-6.604	1.00	0.00	H
ATOM	395	H6	Y7	23	39.506	70.582	-10.470	1.00	0.00	H
ATOM	396	H7	Y7	23	38.478	71.486	-11.626	1.00	0.00	H
ATOM	397	H8	Y7	23	40.231	71.757	-11.578	1.00	0.00	H
ATOM	398	H9	Y7	23	29.404	67.667	-7.878	1.00	0.00	H
ATOM	399	H10	Y7	23	30.501	68.705	-8.797	1.00	0.00	H
ATOM	400	H11	Y7	23	30.806	66.959	-8.745	1.00	0.00	H
ATOM	401	H12	Y7	23	24.923	63.424	-7.472	1.00	0.00	H
ATOM	402	H13	Y7	23	26.114	64.410	-8.374	1.00	0.00	H
ATOM	403	H14	Y7	23	25.058	64.887	-5.625	1.00	0.00	H
ATOM	404	H15	Y7	23	21.996	66.437	-9.352	1.00	0.00	H
ATOM	405	H16	Y7	23	21.828	67.318	-7.831	1.00	0.00	H
ATOM	406	H17	Y7	23	24.199	68.087	-9.540	1.00	0.00	H
ATOM	407	H18	Y7	23	22.640	68.417	-10.260	1.00	0.00	H
ATOM	408	H19	Y7	23	21.979	69.987	-8.651	1.00	0.00	H
ATOM	409	H20	Y7	23	23.007	69.351	-7.385	1.00	0.00	H
ATOM	410	H21	Y7	23	24.137	70.780	-9.862	1.00	0.00	H
ATOM	411	H22	Y7	23	23.707	71.614	-8.367	1.00	0.00	H
ATOM	412	H23	Y7	23	25.289	70.173	-7.076	1.00	0.00	H
ATOM	413	H24	Y7	23	25.765	69.340	-8.570	1.00	0.00	H
ATOM	414	H25	Y7	23	26.160	72.205	-8.806	1.00	0.00	H
ATOM	415	H26	Y7	23	30.447	74.705	-6.802	1.00	0.00	H
ATOM	416	H27	Y7	23	29.669	74.942	-8.377	1.00	0.00	H
ATOM	417	H28	Y7	23	29.505	72.470	-6.579	1.00	0.00	H
ATOM	418	H29	Y7	23	28.205	73.600	-6.959	1.00	0.00	H
ATOM	419	H30	Y7	23	28.171	72.881	-9.310	1.00	0.00	H
ATOM	420	H31	Y7	23	30.495	70.994	-8.658	1.00	0.00	H
ATOM	421	H32	Y7	23	29.720	71.194	-10.228	1.00	0.00	H
ATOM	422	H33	Y7	23	31.879	72.402	-10.062	1.00	0.00	H
ATOM	423	H34	Y7	23	30.545	73.528	-10.374	1.00	0.00	H
ATOM	424	H35	Y7	23	40.151	74.188	-11.878	1.00	0.00	H
ATOM	425	H36	Y7	23	42.025	75.793	-11.617	1.00	0.00	H
ATOM	426	H37	Y7	23	43.144	76.063	-9.410	1.00	0.00	H
ATOM	427	H38	Y7	23	42.387	74.715	-7.458	1.00	0.00	H
ATOM	428	H39	Y7	23	40.519	73.097	-7.710	1.00	0.00	H
ATOM	429	H40	Y7	23	32.441	68.231	-4.540	1.00	0.00	H
ATOM	430	H41	Y7	23	32.426	66.564	-2.726	1.00	0.00	H
ATOM	431	H42	Y7	23	31.251	64.386	-3.028	1.00	0.00	H
ATOM	432	H43	Y7	23	30.064	63.908	-5.169	1.00	0.00	H
ATOM	433	H44	Y7	23	30.080	65.553	-6.992	1.00	0.00	H
ATOM	434	H45	Y7	23	32.001	72.337	-5.703	1.00	0.00	H
ATOM	435	H46	Y7	23	30.673	70.243	-5.699	1.00	0.00	H
ATOM	436	H47	Y7	23	33.608	68.343	-8.182	1.00	0.00	H
ATOM	437	H48	Y7	23	37.375	70.546	-9.608	1.00	0.00	H
ATOM	438	H49	Y7	23	38.188	74.700	-8.589	1.00	0.00	H
ATOM	439	H50	Y7	23	36.135	74.804	-7.298	1.00	0.00	H
ATOM	440	H51	Y7	23	32.286	76.954	-6.816	1.00	0.00	H
ATOM	441	H52	Y7	23	33.109	77.346	-4.510	1.00	0.00	H
ATOM	442	H53	Y7	23	34.484	75.619	-3.358	1.00	0.00	H
ATOM	443	H54	Y7	23	35.048	73.497	-4.526	1.00	0.00	H
ATOM	444	N	ALA	24	26.819	70.419	-4.755	1.00	0.00	N
ATOM	445	H	ALA	24	26.122	69.928	-5.292	1.00	0.00	H
ATOM	446	CA	ALA	24	26.278	71.436	-3.858	1.00	0.00	C
ATOM	447	HA	ALA	24	26.923	71.529	-2.982	1.00	0.00	H
ATOM	448	CB	ALA	24	26.255	72.783	-4.589	1.00	0.00	C
ATOM	449	HB1	ALA	24	25.834	73.552	-3.940	1.00	0.00	H
ATOM	450	HB2	ALA	24	27.273	73.068	-4.851	1.00	0.00	H
ATOM	451	HB3	ALA	24	25.651	72.709	-5.495	1.00	0.00	H
ATOM	452	C	ALA	24	24.878	71.014	-3.370	1.00	0.00	C

```
ATOM    453  O    ALA    24      24.221  70.187  -4.007  1.00  0.00           O
ATOM    454  N    LYS    25      24.411  71.587  -2.257  1.00  0.00           N
ATOM    455  H    LYS    25      24.977  72.313  -1.833  1.00  0.00           H
ATOM    456  CA   LYS    25      23.070  71.397  -1.671  1.00  0.00           C
ATOM    457  HA   LYS    25      22.412  70.922  -2.397  1.00  0.00           H
ATOM    458  CB   LYS    25      23.156  70.530  -0.395  1.00  0.00           C
ATOM    459  HB2 LYS    25      23.807  71.054   0.309  1.00  0.00           H
ATOM    460  HB3 LYS    25      22.171  70.478   0.069  1.00  0.00           H
ATOM    461  CG   LYS    25      23.700  69.094  -0.538  1.00  0.00           C
ATOM    462  HG2 LYS    25      24.609  69.096  -1.133  1.00  0.00           H
ATOM    463  HG3 LYS    25      23.985  68.771   0.465  1.00  0.00           H
ATOM    464  CD   LYS    25      22.710  68.039  -1.070  1.00  0.00           C
ATOM    465  HD2 LYS    25      23.051  67.058  -0.737  1.00  0.00           H
ATOM    466  HD3 LYS    25      21.736  68.208  -0.609  1.00  0.00           H
ATOM    467  CE   LYS    25      22.534  67.990  -2.593  1.00  0.00           C
ATOM    468  HE2 LYS    25      21.700  67.325  -2.830  1.00  0.00           H
ATOM    469  HE3 LYS    25      22.286  68.989  -2.960  1.00  0.00           H
ATOM    470  NZ   LYS    25      23.748  67.504  -3.285  1.00  0.00           N
ATOM    471  HZ1 LYS    25      24.550  68.097  -3.087  1.00  0.00           H
ATOM    472  HZ2 LYS    25      24.009  66.554  -3.035  1.00  0.00           H
ATOM    473  HZ3 LYS    25      23.623  67.521  -4.294  1.00  0.00           H
ATOM    474  C    LYS    25      22.463  72.770  -1.342  1.00  0.00           C
ATOM    475  O    LYS    25      23.200  73.745  -1.233  1.00  0.00           O
ATOM    476  N    NME    26      21.138  72.836  -1.176  1.00  0.00           N
ATOM    477  H    NME    26      20.603  71.990  -1.269  1.00  0.00           H
ATOM    478  CH3 NME    26      20.417  74.067  -0.846  1.00  0.00           C
ATOM    479 HH31 NME    26      20.581  74.815  -1.625  1.00  0.00           H
ATOM    480 HH32 NME    26      19.347  73.872  -0.759  1.00  0.00           H
ATOM    481 HH33 NME    26      20.784  74.471   0.100  1.00  0.00           H
TER    482       NME    26
END
```

Supplementary Table 17. List of atoms and their coordination in the structure #227 of cR6G-P2-QSY7.

ATOM	1	H1	ACE	1	18.498	25.203	-12.231	1.00	0.00	H
ATOM	2	CH3	ACE	1	18.954	24.471	-11.567	1.00	0.00	C
ATOM	3	H2	ACE	1	18.791	24.771	-10.534	1.00	0.00	H
ATOM	4	H3	ACE	1	20.023	24.411	-11.761	1.00	0.00	H
ATOM	5	C	ACE	1	18.318	23.120	-11.806	1.00	0.00	C
ATOM	6	O	ACE	1	17.350	23.015	-12.556	1.00	0.00	O
ATOM	7	N	LYS	2	18.861	22.067	-11.191	1.00	0.00	N
ATOM	8	H	LYS	2	19.573	22.260	-10.491	1.00	0.00	H
ATOM	9	CA	LYS	2	18.503	20.666	-11.477	1.00	0.00	C
ATOM	10	HA	LYS	2	17.789	20.664	-12.299	1.00	0.00	H
ATOM	11	CB	LYS	2	19.759	19.910	-11.956	1.00	0.00	C
ATOM	12	HB2	LYS	2	20.336	20.547	-12.630	1.00	0.00	H
ATOM	13	HB3	LYS	2	20.394	19.684	-11.097	1.00	0.00	H
ATOM	14	CG	LYS	2	19.427	18.601	-12.694	1.00	0.00	C
ATOM	15	HG2	LYS	2	20.336	18.001	-12.764	1.00	0.00	H
ATOM	16	HG3	LYS	2	18.695	18.043	-12.112	1.00	0.00	H
ATOM	17	CD	LYS	2	18.899	18.848	-14.121	1.00	0.00	C
ATOM	18	HD2	LYS	2	18.029	19.506	-14.095	1.00	0.00	H
ATOM	19	HD3	LYS	2	19.677	19.349	-14.700	1.00	0.00	H
ATOM	20	CE	LYS	2	18.510	17.557	-14.854	1.00	0.00	C
ATOM	21	HE2	LYS	2	18.227	17.818	-15.878	1.00	0.00	H
ATOM	22	HE3	LYS	2	19.373	16.887	-14.900	1.00	0.00	H
ATOM	23	NZ	LYS	2	17.373	16.885	-14.187	1.00	0.00	N
ATOM	24	HZ1	LYS	2	16.924	16.176	-14.757	1.00	0.00	H
ATOM	25	HZ2	LYS	2	17.649	16.448	-13.309	1.00	0.00	H
ATOM	26	HZ3	LYS	2	16.645	17.549	-13.934	1.00	0.00	H
ATOM	27	C	LYS	2	17.799	19.998	-10.290	1.00	0.00	C
ATOM	28	O	LYS	2	18.435	19.456	-9.387	1.00	0.00	O
ATOM	29	N	ALA	3	16.467	20.026	-10.295	1.00	0.00	N
ATOM	30	H	ALA	3	16.011	20.448	-11.084	1.00	0.00	H
ATOM	31	CA	ALA	3	15.618	19.410	-9.274	1.00	0.00	C
ATOM	32	HA	ALA	3	16.028	19.641	-8.290	1.00	0.00	H
ATOM	33	CB	ALA	3	14.226	20.054	-9.356	1.00	0.00	C
ATOM	34	HB1	ALA	3	13.786	19.884	-10.338	1.00	0.00	H
ATOM	35	HB2	ALA	3	13.575	19.616	-8.598	1.00	0.00	H
ATOM	36	HB3	ALA	3	14.303	21.125	-9.176	1.00	0.00	H
ATOM	37	C	ALA	3	15.597	17.871	-9.408	1.00	0.00	C
ATOM	38	O	ALA	3	14.627	17.284	-9.876	1.00	0.00	O
ATOM	39	N	QS	4	16.733	17.205	-8.996	1.00	0.00	N
ATOM	40	C1	QS	4	17.062	15.789	-9.300	1.00	0.00	C
ATOM	41	C2	QS	4	16.723	15.388	-10.767	1.00	0.00	C
ATOM	42	C3	QS	4	16.597	14.782	-8.203	1.00	0.00	C
ATOM	43	O1	QS	4	17.441	15.727	-11.696	1.00	0.00	O
ATOM	44	S	QS	4	14.758	14.764	-7.932	1.00	0.00	S
ATOM	45	C4	QS	4	13.662	5.473	-2.381	1.00	0.00	C
ATOM	46	C5	QS	4	14.265	5.692	-3.596	1.00	0.00	C
ATOM	47	C6	QS	4	14.599	7.021	-4.041	1.00	0.00	C
ATOM	48	C7	QS	4	14.249	8.122	-3.168	1.00	0.00	C
ATOM	49	C8	QS	4	13.586	7.941	-1.959	1.00	0.00	C
ATOM	50	C9	QS	4	13.319	6.575	-1.522	1.00	0.00	C
ATOM	51	C10	QS	4	15.205	7.207	-5.260	1.00	0.00	C
ATOM	52	N2	QS	4	15.572	8.461	-5.763	1.00	0.00	N
ATOM	53	O2	QS	4	15.464	6.274	-6.009	1.00	0.00	O
ATOM	54	C11	QS	4	15.978	8.698	-7.158	1.00	0.00	C
ATOM	55	C12	QS	4	16.717	10.046	-7.316	1.00	0.00	C
ATOM	56	N3	QS	4	15.868	11.219	-6.989	1.00	0.00	N
ATOM	57	C13	QS	4	15.225	11.967	-7.930	1.00	0.00	C
ATOM	58	C14	QS	4	14.428	13.070	-7.239	1.00	0.00	C
ATOM	59	C15	QS	4	14.827	12.929	-5.744	1.00	0.00	C
ATOM	60	C16	QS	4	15.630	11.644	-5.715	1.00	0.00	C
ATOM	61	O3	QS	4	15.997	11.064	-4.715	1.00	0.00	O
ATOM	62	O4	QS	4	15.342	11.829	-9.131	1.00	0.00	O
ATOM	63	C17	QS	4	12.770	6.272	-0.275	1.00	0.00	C
ATOM	64	C18	QS	4	11.699	5.270	-0.194	1.00	0.00	C
ATOM	65	C19	QS	4	11.383	4.758	1.071	1.00	0.00	C
ATOM	66	O5	QS	4	12.048	5.188	2.188	1.00	0.00	O
ATOM	67	C20	QS	4	13.120	6.041	2.143	1.00	0.00	C
ATOM	68	C21	QS	4	13.556	6.593	0.929	1.00	0.00	C
ATOM	69	C22	QS	4	13.796	6.297	3.335	1.00	0.00	C
ATOM	70	C23	QS	4	14.944	7.092	3.344	1.00	0.00	C
ATOM	71	C24	QS	4	15.436	7.613	2.129	1.00	0.00	C
ATOM	72	C25	QS	4	14.749	7.350	0.928	1.00	0.00	C
ATOM	73	C26	QS	4	10.897	4.897	-1.294	1.00	0.00	C
ATOM	74	C27	QS	4	9.864	3.953	-1.141	1.00	0.00	C

ATOM	75	C28	QS	4	9.601	3.402	0.131	1.00	0.00	C
ATOM	76	C29	QS	4	10.356	3.827	1.231	1.00	0.00	C
ATOM	77	C30	QS	4	9.036	3.552	-2.365	1.00	0.00	C
ATOM	78	C31	QS	4	16.713	8.455	2.088	1.00	0.00	C
ATOM	79	N4	QS	4	15.618	7.349	4.529	1.00	0.00	N
ATOM	80	N5	QS	4	8.579	2.470	0.275	1.00	0.00	N
ATOM	81	C32	QS	4	15.202	6.894	5.864	1.00	0.00	C
ATOM	82	C33	QS	4	16.159	7.454	6.929	1.00	0.00	C
ATOM	83	C34	QS	4	8.226	1.740	1.504	1.00	0.00	C
ATOM	84	C35	QS	4	7.239	2.568	2.347	1.00	0.00	C
ATOM	85	C36	QS	4	13.141	9.185	-1.247	1.00	0.00	C
ATOM	86	O7	QS	4	13.405	10.314	-1.603	1.00	0.00	O
ATOM	87	O8	QS	4	12.284	9.029	-0.241	1.00	0.00	O
ATOM	88	H1	QS	4	17.513	17.809	-8.738	1.00	0.00	H
ATOM	89	H2	QS	4	18.158	15.734	-9.291	1.00	0.00	H
ATOM	90	H3	QS	4	16.948	13.783	-8.490	1.00	0.00	H
ATOM	91	H4	QS	4	17.077	15.042	-7.251	1.00	0.00	H
ATOM	92	H6	QS	4	13.482	4.453	-2.056	1.00	0.00	H
ATOM	93	H7	QS	4	14.519	4.841	-4.225	1.00	0.00	H
ATOM	94	H8	QS	4	14.470	9.134	-3.495	1.00	0.00	H
ATOM	95	H9	QS	4	15.713	9.214	-5.093	1.00	0.00	H
ATOM	96	H10	QS	4	16.646	7.889	-7.485	1.00	0.00	H
ATOM	97	H11	QS	4	15.091	8.679	-7.805	1.00	0.00	H
ATOM	98	H12	QS	4	17.600	10.046	-6.662	1.00	0.00	H
ATOM	99	H13	QS	4	17.077	10.131	-8.351	1.00	0.00	H
ATOM	100	H14	QS	4	13.369	12.815	-7.374	1.00	0.00	H
ATOM	101	H15	QS	4	13.956	12.837	-5.084	1.00	0.00	H
ATOM	102	H16	QS	4	15.466	13.750	-5.401	1.00	0.00	H
ATOM	103	H17	QS	4	13.415	5.850	4.243	1.00	0.00	H
ATOM	104	H18	QS	4	15.166	7.733	0.002	1.00	0.00	H
ATOM	105	H19	QS	4	11.061	5.345	-2.271	1.00	0.00	H
ATOM	106	H20	QS	4	10.154	3.458	2.228	1.00	0.00	H
ATOM	107	H21	QS	4	9.114	2.475	-2.560	1.00	0.00	H
ATOM	108	H22	QS	4	7.976	3.799	-2.224	1.00	0.00	H
ATOM	109	H23	QS	4	9.372	4.070	-3.273	1.00	0.00	H
ATOM	110	H24	QS	4	17.576	7.881	2.449	1.00	0.00	H
ATOM	111	H25	QS	4	16.950	8.790	1.070	1.00	0.00	H
ATOM	112	H26	QS	4	16.614	9.353	2.710	1.00	0.00	H
ATOM	113	H27	QS	4	16.468	7.889	4.460	1.00	0.00	H
ATOM	114	H28	QS	4	8.065	2.237	-0.564	1.00	0.00	H
ATOM	115	H29	QS	4	14.181	7.237	6.080	1.00	0.00	H
ATOM	116	H30	QS	4	15.212	5.796	5.910	1.00	0.00	H
ATOM	117	H31	QS	4	16.154	8.551	6.922	1.00	0.00	H
ATOM	118	H32	QS	4	15.853	7.120	7.929	1.00	0.00	H
ATOM	119	H33	QS	4	17.185	7.107	6.756	1.00	0.00	H
ATOM	120	H34	QS	4	7.752	0.784	1.242	1.00	0.00	H
ATOM	121	H35	QS	4	9.121	1.506	2.095	1.00	0.00	H
ATOM	122	H36	QS	4	7.679	3.530	2.640	1.00	0.00	H
ATOM	123	H37	QS	4	6.319	2.769	1.784	1.00	0.00	H
ATOM	124	H38	QS	4	6.966	2.026	3.261	1.00	0.00	H
ATOM	125	H40	QS	4	12.115	9.963	-0.036	1.00	0.00	H
ATOM	126	N	ALA	5	15.592	14.637	-11.004	1.00	0.00	N
ATOM	127	H	ALA	5	15.021	14.370	-10.215	1.00	0.00	H
ATOM	128	CA	ALA	5	15.122	14.277	-12.344	1.00	0.00	C
ATOM	129	HA	ALA	5	15.950	13.832	-12.899	1.00	0.00	H
ATOM	130	CB	ALA	5	14.027	13.214	-12.195	1.00	0.00	C
ATOM	131	HB1	ALA	5	13.674	12.912	-13.183	1.00	0.00	H
ATOM	132	HB2	ALA	5	14.427	12.336	-11.686	1.00	0.00	H
ATOM	133	HB3	ALA	5	13.186	13.610	-11.624	1.00	0.00	H
ATOM	134	C	ALA	5	14.618	15.493	-13.148	1.00	0.00	C
ATOM	135	O	ALA	5	14.927	15.620	-14.333	1.00	0.00	O
ATOM	136	N	ALA	6	13.915	16.424	-12.499	1.00	0.00	N
ATOM	137	H	ALA	6	13.825	16.326	-11.494	1.00	0.00	H
ATOM	138	CA	ALA	6	13.345	17.637	-13.088	1.00	0.00	C
ATOM	139	HA	ALA	6	13.055	17.429	-14.120	1.00	0.00	H
ATOM	140	CB	ALA	6	12.064	17.948	-12.295	1.00	0.00	C
ATOM	141	HB1	ALA	6	12.309	18.192	-11.260	1.00	0.00	H
ATOM	142	HB2	ALA	6	11.526	18.783	-12.745	1.00	0.00	H
ATOM	143	HB3	ALA	6	11.405	17.078	-12.305	1.00	0.00	H
ATOM	144	C	ALA	6	14.347	18.819	-13.117	1.00	0.00	C
ATOM	145	O	ALA	6	15.562	18.642	-12.968	1.00	0.00	O
ATOM	146	N	ALA	7	13.846	20.040	-13.314	1.00	0.00	N
ATOM	147	H	ALA	7	12.844	20.137	-13.413	1.00	0.00	H
ATOM	148	CA	ALA	7	14.606	21.290	-13.289	1.00	0.00	C
ATOM	149	HA	ALA	7	15.556	21.128	-12.783	1.00	0.00	H
ATOM	150	CB	ALA	7	14.915	21.719	-14.728	1.00	0.00	C

ATOM	151	HB1	ALA	7	15.510	22.634	-14.720	1.00	0.00	H
ATOM	152	HB2	ALA	7	15.478	20.937	-15.238	1.00	0.00	H
ATOM	153	HB3	ALA	7	13.986	21.905	-15.270	1.00	0.00	H
ATOM	154	C	ALA	7	13.840	22.374	-12.509	1.00	0.00	C
ATOM	155	O	ALA	7	12.613	22.414	-12.543	1.00	0.00	O
ATOM	156	N	HIE	8	14.566	23.235	-11.792	1.00	0.00	N
ATOM	157	H	HIE	8	15.578	23.170	-11.864	1.00	0.00	H
ATOM	158	CA	HIE	8	14.038	24.399	-11.065	1.00	0.00	C
ATOM	159	HA	HIE	8	13.331	24.929	-11.706	1.00	0.00	H
ATOM	160	CB	HIE	8	13.308	23.964	-9.782	1.00	0.00	C
ATOM	161	HB2	HIE	8	12.566	23.204	-10.025	1.00	0.00	H
ATOM	162	HB3	HIE	8	14.025	23.525	-9.088	1.00	0.00	H
ATOM	163	CG	HIE	8	12.606	25.104	-9.087	1.00	0.00	C
ATOM	164	ND1	HIE	8	13.231	26.172	-8.437	1.00	0.00	N
ATOM	165	CE1	HIE	8	12.239	26.947	-7.969	1.00	0.00	C
ATOM	166	HE1	HIE	8	12.383	27.859	-7.401	1.00	0.00	H
ATOM	167	NE2	HIE	8	11.043	26.432	-8.301	1.00	0.00	N
ATOM	168	HE2	HIE	8	10.144	26.829	-8.057	1.00	0.00	H
ATOM	169	CD2	HIE	8	11.255	25.271	-9.009	1.00	0.00	C
ATOM	170	HD2	HIE	8	10.503	24.610	-9.423	1.00	0.00	H
ATOM	171	C	HIE	8	15.196	25.333	-10.706	1.00	0.00	C
ATOM	172	O	HIE	8	16.207	24.867	-10.184	1.00	0.00	O
ATOM	173	N	ALA	9	15.062	26.639	-10.947	1.00	0.00	N
ATOM	174	H	ALA	9	14.182	26.974	-11.309	1.00	0.00	H
ATOM	175	CA	ALA	9	16.157	27.607	-10.811	1.00	0.00	C
ATOM	176	HA	ALA	9	16.939	27.335	-11.521	1.00	0.00	H
ATOM	177	CB	ALA	9	15.613	28.986	-11.204	1.00	0.00	C
ATOM	178	HB1	ALA	9	14.820	29.288	-10.517	1.00	0.00	H
ATOM	179	HB2	ALA	9	16.417	29.721	-11.162	1.00	0.00	H
ATOM	180	HB3	ALA	9	15.219	28.957	-12.221	1.00	0.00	H
ATOM	181	C	ALA	9	16.819	27.651	-9.415	1.00	0.00	C
ATOM	182	O	ALA	9	18.002	27.973	-9.316	1.00	0.00	O
ATOM	183	N	ALA	10	16.096	27.306	-8.343	1.00	0.00	N
ATOM	184	H	ALA	10	15.132	27.020	-8.479	1.00	0.00	H
ATOM	185	CA	ALA	10	16.646	27.251	-6.987	1.00	0.00	C
ATOM	186	HA	ALA	10	17.256	28.140	-6.820	1.00	0.00	H
ATOM	187	CB	ALA	10	15.475	27.288	-5.997	1.00	0.00	C
ATOM	188	HB1	ALA	10	14.869	28.179	-6.169	1.00	0.00	H
ATOM	189	HB2	ALA	10	14.854	26.399	-6.113	1.00	0.00	H
ATOM	190	HB3	ALA	10	15.861	27.319	-4.977	1.00	0.00	H
ATOM	191	C	ALA	10	17.543	26.021	-6.730	1.00	0.00	C
ATOM	192	O	ALA	10	18.421	26.073	-5.866	1.00	0.00	O
ATOM	193	N	ALA	11	17.350	24.916	-7.463	1.00	0.00	N
ATOM	194	H	ALA	11	16.690	24.965	-8.232	1.00	0.00	H
ATOM	195	CA	ALA	11	18.015	23.631	-7.231	1.00	0.00	C
ATOM	196	HA	ALA	11	17.976	23.417	-6.161	1.00	0.00	H
ATOM	197	CB	ALA	11	17.212	22.534	-7.941	1.00	0.00	C
ATOM	198	HB1	ALA	11	17.673	21.566	-7.747	1.00	0.00	H
ATOM	199	HB2	ALA	11	16.189	22.522	-7.565	1.00	0.00	H
ATOM	200	HB3	ALA	11	17.195	22.717	-9.014	1.00	0.00	H
ATOM	201	C	ALA	11	19.505	23.672	-7.637	1.00	0.00	C
ATOM	202	O	ALA	11	19.895	23.234	-8.720	1.00	0.00	O
ATOM	203	N	HIP	12	20.330	24.242	-6.750	1.00	0.00	N
ATOM	204	H	HIP	12	19.866	24.598	-5.920	1.00	0.00	H
ATOM	205	CA	HIP	12	21.664	24.789	-7.032	1.00	0.00	C
ATOM	206	HA	HIP	12	22.010	25.247	-6.104	1.00	0.00	H
ATOM	207	CB	HIP	12	22.685	23.680	-7.369	1.00	0.00	C
ATOM	208	HB2	HIP	12	22.588	22.879	-6.634	1.00	0.00	H
ATOM	209	HB3	HIP	12	22.455	23.258	-8.349	1.00	0.00	H
ATOM	210	CG	HIP	12	24.125	24.158	-7.380	1.00	0.00	C
ATOM	211	ND1	HIP	12	24.615	25.268	-8.049	1.00	0.00	N
ATOM	212	HD1	HIP	12	24.044	25.909	-8.610	1.00	0.00	H
ATOM	213	CE1	HIP	12	25.939	25.353	-7.818	1.00	0.00	C
ATOM	214	HE1	HIP	12	26.593	26.128	-8.206	1.00	0.00	H
ATOM	215	NE2	HIP	12	26.306	24.320	-7.028	1.00	0.00	N
ATOM	216	HE2	HIP	12	27.248	24.124	-6.699	1.00	0.00	H
ATOM	217	CD2	HIP	12	25.181	23.562	-6.745	1.00	0.00	C
ATOM	218	HD2	HIP	12	25.136	22.661	-6.141	1.00	0.00	H
ATOM	219	C	HIP	12	21.606	25.937	-8.056	1.00	0.00	C
ATOM	220	O	HIP	12	22.128	25.824	-9.165	1.00	0.00	O
ATOM	221	N	ALA	13	21.057	27.074	-7.632	1.00	0.00	N
ATOM	222	H	ALA	13	20.578	27.074	-6.744	1.00	0.00	H
ATOM	223	CA	ALA	13	21.142	28.346	-8.353	1.00	0.00	C
ATOM	224	HA	ALA	13	20.589	28.245	-9.290	1.00	0.00	H
ATOM	225	CB	ALA	13	20.441	29.411	-7.497	1.00	0.00	C
ATOM	226	HB1	ALA	13	19.422	29.096	-7.269	1.00	0.00	H

ATOM	227	HB2	ALA	13	20.987	29.562	-6.564	1.00	0.00	H
ATOM	228	HB3	ALA	13	20.395	30.357	-8.037	1.00	0.00	H
ATOM	229	C	ALA	13	22.591	28.776	-8.706	1.00	0.00	C
ATOM	230	O	ALA	13	23.578	28.198	-8.232	1.00	0.00	O
ATOM	231	N	ALA	14	22.710	29.805	-9.551	1.00	0.00	N
ATOM	232	H	ALA	14	21.862	30.239	-9.879	1.00	0.00	H
ATOM	233	CA	ALA	14	23.969	30.484	-9.864	1.00	0.00	C
ATOM	234	HA	ALA	14	24.758	29.735	-9.947	1.00	0.00	H
ATOM	235	CB	ALA	14	23.819	31.171	-11.228	1.00	0.00	C
ATOM	236	HB1	ALA	14	23.550	30.436	-11.987	1.00	0.00	H
ATOM	237	HB2	ALA	14	23.043	31.936	-11.177	1.00	0.00	H
ATOM	238	HB3	ALA	14	24.761	31.642	-11.513	1.00	0.00	H
ATOM	239	C	ALA	14	24.384	31.490	-8.767	1.00	0.00	C
ATOM	240	O	ALA	14	23.555	31.966	-7.994	1.00	0.00	O
ATOM	241	N	ALA	15	25.672	31.845	-8.713	1.00	0.00	N
ATOM	242	H	ALA	15	26.309	31.464	-9.393	1.00	0.00	H
ATOM	243	CA	ALA	15	26.192	32.828	-7.760	1.00	0.00	C
ATOM	244	HA	ALA	15	25.790	32.587	-6.774	1.00	0.00	H
ATOM	245	CB	ALA	15	27.718	32.692	-7.694	1.00	0.00	C
ATOM	246	HB1	ALA	15	27.989	31.673	-7.413	1.00	0.00	H
ATOM	247	HB2	ALA	15	28.158	32.927	-8.665	1.00	0.00	H
ATOM	248	HB3	ALA	15	28.119	33.378	-6.946	1.00	0.00	H
ATOM	249	C	ALA	15	25.767	34.272	-8.106	1.00	0.00	C
ATOM	250	O	ALA	15	25.808	34.685	-9.265	1.00	0.00	O
ATOM	251	N	ALA	16	25.438	35.073	-7.084	1.00	0.00	N
ATOM	252	H	ALA	16	25.403	34.665	-6.162	1.00	0.00	H
ATOM	253	CA	ALA	16	24.895	36.432	-7.229	1.00	0.00	C
ATOM	254	HA	ALA	16	24.023	36.378	-7.884	1.00	0.00	H
ATOM	255	CB	ALA	16	24.415	36.888	-5.845	1.00	0.00	C
ATOM	256	HB1	ALA	16	25.257	36.958	-5.155	1.00	0.00	H
ATOM	257	HB2	ALA	16	23.942	37.868	-5.927	1.00	0.00	H
ATOM	258	HB3	ALA	16	23.682	36.182	-5.452	1.00	0.00	H
ATOM	259	C	ALA	16	25.851	37.477	-7.855	1.00	0.00	C
ATOM	260	O	ALA	16	25.400	38.554	-8.238	1.00	0.00	O
ATOM	261	N	HIP	17	27.154	37.174	-7.971	1.00	0.00	N
ATOM	262	H	HIP	17	27.425	36.259	-7.646	1.00	0.00	H
ATOM	263	CA	HIP	17	28.167	37.957	-8.712	1.00	0.00	C
ATOM	264	HA	HIP	17	29.142	37.617	-8.363	1.00	0.00	H
ATOM	265	CB	HIP	17	28.102	37.598	-10.216	1.00	0.00	C
ATOM	266	HB2	HIP	17	28.932	38.088	-10.729	1.00	0.00	H
ATOM	267	HB3	HIP	17	28.250	36.522	-10.326	1.00	0.00	H
ATOM	268	CG	HIP	17	26.820	37.981	-10.924	1.00	0.00	C
ATOM	269	ND1	HIP	17	26.359	39.270	-11.099	1.00	0.00	N
ATOM	270	HD1	HIP	17	26.828	40.092	-10.714	1.00	0.00	H
ATOM	271	CE1	HIP	17	25.149	39.213	-11.690	1.00	0.00	C
ATOM	272	HE1	HIP	17	24.513	40.067	-11.905	1.00	0.00	H
ATOM	273	NE2	HIP	17	24.840	37.916	-11.908	1.00	0.00	N
ATOM	274	HE2	HIP	17	23.975	37.571	-12.313	1.00	0.00	H
ATOM	275	CD2	HIP	17	25.874	37.129	-11.425	1.00	0.00	C
ATOM	276	HD2	HIP	17	25.914	36.045	-11.398	1.00	0.00	H
ATOM	277	C	HIP	17	28.171	39.476	-8.405	1.00	0.00	C
ATOM	278	O	HIP	17	28.177	40.304	-9.318	1.00	0.00	O
ATOM	279	N	ALA	18	28.134	39.844	-7.122	1.00	0.00	N
ATOM	280	H	ALA	18	28.119	39.127	-6.418	1.00	0.00	H
ATOM	281	CA	ALA	18	28.305	41.229	-6.675	1.00	0.00	C
ATOM	282	HA	ALA	18	27.612	41.862	-7.232	1.00	0.00	H
ATOM	283	CB	ALA	18	27.939	41.311	-5.188	1.00	0.00	C
ATOM	284	HB1	ALA	18	26.916	40.965	-5.035	1.00	0.00	H
ATOM	285	HB2	ALA	18	28.622	40.695	-4.600	1.00	0.00	H
ATOM	286	HB3	ALA	18	28.014	42.344	-4.844	1.00	0.00	H
ATOM	287	C	ALA	18	29.737	41.749	-6.939	1.00	0.00	C
ATOM	288	O	ALA	18	30.678	40.963	-7.043	1.00	0.00	O
ATOM	289	N	ALA	19	29.906	43.071	-7.035	1.00	0.00	N
ATOM	290	H	ALA	19	29.111	43.676	-6.906	1.00	0.00	H
ATOM	291	CA	ALA	19	31.213	43.698	-7.239	1.00	0.00	C
ATOM	292	HA	ALA	19	31.718	43.175	-8.054	1.00	0.00	H
ATOM	293	CB	ALA	19	31.003	45.154	-7.671	1.00	0.00	C
ATOM	294	HB1	ALA	19	30.494	45.709	-6.881	1.00	0.00	H
ATOM	295	HB2	ALA	19	31.968	45.622	-7.868	1.00	0.00	H
ATOM	296	HB3	ALA	19	30.403	45.190	-8.581	1.00	0.00	H
ATOM	297	C	ALA	19	32.103	43.600	-5.983	1.00	0.00	C
ATOM	298	O	ALA	19	31.671	43.922	-4.876	1.00	0.00	O
ATOM	299	N	ALA	20	33.366	43.200	-6.159	1.00	0.00	N
ATOM	300	H	ALA	20	33.657	42.953	-7.092	1.00	0.00	H
ATOM	301	CA	ALA	20	34.334	42.965	-5.083	1.00	0.00	C
ATOM	302	HA	ALA	20	33.820	42.434	-4.280	1.00	0.00	H

ATOM	303	CB	ALA	20	35.422	42.027	-5.628	1.00	0.00	C
ATOM	304	HB1	ALA	20	35.969	42.514	-6.436	1.00	0.00	H
ATOM	305	HB2	ALA	20	36.123	41.776	-4.831	1.00	0.00	H
ATOM	306	HB3	ALA	20	34.970	41.105	-5.999	1.00	0.00	H
ATOM	307	C	ALA	20	34.905	44.264	-4.460	1.00	0.00	C
ATOM	308	O	ALA	20	36.108	44.518	-4.505	1.00	0.00	O
ATOM	309	N	HIP	21	34.050	45.110	-3.871	1.00	0.00	N
ATOM	310	H	HIP	21	33.066	44.858	-3.881	1.00	0.00	H
ATOM	311	CA	HIP	21	34.475	46.316	-3.143	1.00	0.00	C
ATOM	312	HA	HIP	21	35.187	46.863	-3.765	1.00	0.00	H
ATOM	313	CB	HIP	21	33.264	47.239	-2.894	1.00	0.00	C
ATOM	314	HB2	HIP	21	32.789	47.463	-3.850	1.00	0.00	H
ATOM	315	HB3	HIP	21	32.531	46.713	-2.278	1.00	0.00	H
ATOM	316	CG	HIP	21	33.613	48.546	-2.206	1.00	0.00	C
ATOM	317	ND1	HIP	21	34.140	48.692	-0.932	1.00	0.00	N
ATOM	318	HD1	HIP	21	34.374	47.944	-0.267	1.00	0.00	H
ATOM	319	CE1	HIP	21	34.347	50.000	-0.705	1.00	0.00	C
ATOM	320	HE1	HIP	21	34.780	50.406	0.203	1.00	0.00	H
ATOM	321	NE2	HIP	21	33.945	50.698	-1.784	1.00	0.00	N
ATOM	322	HE2	HIP	21	33.973	51.720	-1.866	1.00	0.00	H
ATOM	323	CD2	HIP	21	33.486	49.803	-2.737	1.00	0.00	C
ATOM	324	HD2	HIP	21	33.107	50.048	-3.722	1.00	0.00	H
ATOM	325	C	HIP	21	35.205	45.943	-1.841	1.00	0.00	C
ATOM	326	O	HIP	21	34.577	45.837	-0.790	1.00	0.00	O
ATOM	327	N	ALA	22	36.527	45.788	-1.896	1.00	0.00	N
ATOM	328	H	ALA	22	36.972	45.792	-2.803	1.00	0.00	H
ATOM	329	CA	ALA	22	37.364	45.638	-0.705	1.00	0.00	C
ATOM	330	HA	ALA	22	37.039	44.742	-0.174	1.00	0.00	H
ATOM	331	CB	ALA	22	38.818	45.433	-1.145	1.00	0.00	C
ATOM	332	HB1	ALA	22	39.174	46.315	-1.681	1.00	0.00	H
ATOM	333	HB2	ALA	22	39.450	45.271	-0.271	1.00	0.00	H
ATOM	334	HB3	ALA	22	38.891	44.561	-1.798	1.00	0.00	H
ATOM	335	C	ALA	22	37.216	46.841	0.253	1.00	0.00	C
ATOM	336	O	ALA	22	37.043	47.975	-0.196	1.00	0.00	O
ATOM	337	N	ALA	23	37.291	46.573	1.564	1.00	0.00	N
ATOM	338	H	ALA	23	37.446	45.613	1.824	1.00	0.00	H
ATOM	339	CA	ALA	23	37.058	47.523	2.662	1.00	0.00	C
ATOM	340	HA	ALA	23	37.089	46.942	3.585	1.00	0.00	H
ATOM	341	CB	ALA	23	38.234	48.513	2.733	1.00	0.00	C
ATOM	342	HB1	ALA	23	38.224	49.172	1.863	1.00	0.00	H
ATOM	343	HB2	ALA	23	38.162	49.121	3.635	1.00	0.00	H
ATOM	344	HB3	ALA	23	39.179	47.969	2.756	1.00	0.00	H
ATOM	345	C	ALA	23	35.672	48.218	2.651	1.00	0.00	C
ATOM	346	O	ALA	23	34.884	48.090	1.713	1.00	0.00	O
ATOM	347	N	ALA	24	35.366	48.949	3.727	1.00	0.00	N
ATOM	348	H	ALA	24	36.039	48.995	4.476	1.00	0.00	H
ATOM	349	CA	ALA	24	34.175	49.795	3.838	1.00	0.00	C
ATOM	350	HA	ALA	24	33.397	49.413	3.175	1.00	0.00	H
ATOM	351	CB	ALA	24	33.635	49.699	5.274	1.00	0.00	C
ATOM	352	HB1	ALA	24	34.393	50.000	5.997	1.00	0.00	H
ATOM	353	HB2	ALA	24	32.759	50.342	5.381	1.00	0.00	H
ATOM	354	HB3	ALA	24	33.337	48.671	5.484	1.00	0.00	H
ATOM	355	C	ALA	24	34.484	51.233	3.372	1.00	0.00	C
ATOM	356	O	ALA	24	34.371	51.542	2.186	1.00	0.00	O
ATOM	357	N	Y7	25	34.934	52.128	4.317	1.00	0.00	N
ATOM	358	C1	Y7	25	35.393	53.501	4.026	1.00	0.00	C
ATOM	359	C2	Y7	25	36.665	53.843	4.831	1.00	0.00	C
ATOM	360	C3	Y7	25	34.265	54.540	4.283	1.00	0.00	C
ATOM	361	O1	Y7	25	37.054	53.134	5.744	1.00	0.00	O
ATOM	362	S	Y7	25	32.890	54.356	3.064	1.00	0.00	S
ATOM	363	C4	Y7	25	35.381	72.131	0.786	1.00	0.00	C
ATOM	364	C5	Y7	25	40.393	68.200	-6.584	1.00	0.00	C
ATOM	365	O2	Y7	25	35.821	55.836	2.016	1.00	0.00	O
ATOM	366	O3	Y7	25	34.007	53.482	-1.406	1.00	0.00	O
ATOM	367	C6	Y7	25	34.045	54.213	-0.430	1.00	0.00	C
ATOM	368	C7	Y7	25	32.838	54.758	0.311	1.00	0.00	C
ATOM	369	C8	Y7	25	33.443	55.371	1.604	1.00	0.00	C
ATOM	370	C9	Y7	25	34.952	55.330	1.329	1.00	0.00	C
ATOM	371	N2	Y7	25	35.187	54.652	0.169	1.00	0.00	N
ATOM	372	C10	Y7	25	36.526	54.555	-0.445	1.00	0.00	C
ATOM	373	C11	Y7	25	36.990	55.920	-1.009	1.00	0.00	C
ATOM	374	C12	Y7	25	38.310	55.820	-1.817	1.00	0.00	C
ATOM	375	C13	Y7	25	39.010	57.192	-2.028	1.00	0.00	C
ATOM	376	C14	Y7	25	38.086	58.276	-2.629	1.00	0.00	C
ATOM	377	N3	Y7	25	38.797	59.544	-2.838	1.00	0.00	N
ATOM	378	O4	Y7	25	36.921	60.777	-3.034	1.00	0.00	O

ATOM	379	C15	Y7	25	38.134	60.734	-3.043	1.00	0.00	C
ATOM	380	C16	Y7	25	37.598	63.725	-2.031	1.00	0.00	C
ATOM	381	C17	Y7	25	38.179	63.280	-3.396	1.00	0.00	C
ATOM	382	C18	Y7	25	39.022	61.975	-3.282	1.00	0.00	C
ATOM	383	C19	Y7	25	40.096	62.181	-2.173	1.00	0.00	C
ATOM	384	C20	Y7	25	39.460	62.633	-0.838	1.00	0.00	C
ATOM	385	N4	Y7	25	38.667	63.865	-1.016	1.00	0.00	N
ATOM	386	C21	Y7	25	35.155	71.975	3.801	1.00	0.00	C
ATOM	387	C22	Y7	25	35.184	72.475	5.113	1.00	0.00	C
ATOM	388	C23	Y7	25	36.388	72.947	5.659	1.00	0.00	C
ATOM	389	C24	Y7	25	37.563	72.916	4.892	1.00	0.00	C
ATOM	390	C25	Y7	25	37.533	72.414	3.580	1.00	0.00	C
ATOM	391	C26	Y7	25	41.404	65.371	-7.258	1.00	0.00	C
ATOM	392	C27	Y7	25	41.411	64.155	-7.963	1.00	0.00	C
ATOM	393	C28	Y7	25	40.288	63.313	-7.922	1.00	0.00	C
ATOM	394	C29	Y7	25	39.159	63.687	-7.175	1.00	0.00	C
ATOM	395	C30	Y7	25	39.157	64.903	-6.472	1.00	0.00	C
ATOM	396	C31	Y7	25	40.275	65.751	-6.510	1.00	0.00	C
ATOM	397	C32	Y7	25	36.329	71.944	3.022	1.00	0.00	C
ATOM	398	N5	Y7	25	36.308	71.446	1.717	1.00	0.00	N
ATOM	399	N6	Y7	25	40.258	66.949	-5.803	1.00	0.00	N
ATOM	400	C33	Y7	25	40.540	66.169	-2.192	1.00	0.00	C
ATOM	401	C34	Y7	25	40.702	66.100	-3.583	1.00	0.00	C
ATOM	402	C35	Y7	25	40.089	67.039	-4.423	1.00	0.00	C
ATOM	403	C36	Y7	25	39.302	68.046	-3.855	1.00	0.00	C
ATOM	404	C37	Y7	25	37.357	70.241	-0.176	1.00	0.00	C
ATOM	405	C38	Y7	25	37.110	70.417	1.227	1.00	0.00	C
ATOM	406	C39	Y7	25	37.717	69.488	2.139	1.00	0.00	C
ATOM	407	C40	Y7	25	38.513	68.457	1.699	1.00	0.00	C
ATOM	408	C41	Y7	25	39.746	67.184	-1.628	1.00	0.00	C
ATOM	409	C42	Y7	25	39.128	68.113	-2.471	1.00	0.00	C
ATOM	410	O5	Y7	25	38.347	69.121	-1.977	1.00	0.00	O
ATOM	411	C43	Y7	25	38.149	69.220	-0.631	1.00	0.00	C
ATOM	412	C44	Y7	25	38.763	68.304	0.288	1.00	0.00	C
ATOM	413	C45	Y7	25	39.553	67.266	-0.186	1.00	0.00	C
ATOM	414	C46	Y7	25	40.189	66.374	0.796	1.00	0.00	C
ATOM	415	C47	Y7	25	39.621	65.133	1.181	1.00	0.00	C
ATOM	416	C48	Y7	25	40.257	64.355	2.173	1.00	0.00	C
ATOM	417	C49	Y7	25	41.454	64.792	2.760	1.00	0.00	C
ATOM	418	C50	Y7	25	42.027	66.009	2.364	1.00	0.00	C
ATOM	419	C51	Y7	25	41.394	66.794	1.389	1.00	0.00	C
ATOM	420	S1	Y7	25	38.095	64.497	0.444	1.00	0.00	S
ATOM	421	O7	Y7	25	37.231	65.619	0.086	1.00	0.00	O
ATOM	422	O8	Y7	25	37.608	63.348	1.206	1.00	0.00	O
ATOM	423	H	Y7	25	35.094	51.800	5.262	1.00	0.00	H
ATOM	424	H1	Y7	25	35.699	53.537	2.976	1.00	0.00	H
ATOM	425	H2	Y7	25	33.860	54.390	5.292	1.00	0.00	H
ATOM	426	H3	Y7	25	34.653	55.565	4.234	1.00	0.00	H
ATOM	427	H6	Y7	25	34.776	71.397	0.237	1.00	0.00	H
ATOM	428	H7	Y7	25	34.695	72.794	1.330	1.00	0.00	H
ATOM	429	H8	Y7	25	35.942	72.748	0.073	1.00	0.00	H
ATOM	430	H9	Y7	25	40.978	68.941	-6.024	1.00	0.00	H
ATOM	431	H10	Y7	25	40.915	68.015	-7.532	1.00	0.00	H
ATOM	432	H11	Y7	25	39.404	68.618	-6.812	1.00	0.00	H
ATOM	433	H12	Y7	25	32.355	55.523	-0.308	1.00	0.00	H
ATOM	434	H13	Y7	25	32.131	53.946	0.518	1.00	0.00	H
ATOM	435	H14	Y7	25	33.146	56.419	1.734	1.00	0.00	H
ATOM	436	H15	Y7	25	37.245	54.208	0.310	1.00	0.00	H
ATOM	437	H16	Y7	25	36.508	53.813	-1.255	1.00	0.00	H
ATOM	438	H17	Y7	25	36.201	56.332	-1.651	1.00	0.00	H
ATOM	439	H18	Y7	25	37.125	56.623	-0.177	1.00	0.00	H
ATOM	440	H19	Y7	25	39.009	55.152	-1.297	1.00	0.00	H
ATOM	441	H20	Y7	25	38.104	55.359	-2.792	1.00	0.00	H
ATOM	442	H21	Y7	25	39.396	57.548	-1.064	1.00	0.00	H
ATOM	443	H22	Y7	25	39.879	57.050	-2.683	1.00	0.00	H
ATOM	444	H23	Y7	25	37.682	57.931	-3.590	1.00	0.00	H
ATOM	445	H24	Y7	25	37.239	58.458	-1.953	1.00	0.00	H
ATOM	446	H25	Y7	25	39.803	59.553	-2.878	1.00	0.00	H
ATOM	447	H26	Y7	25	36.847	62.997	-1.692	1.00	0.00	H
ATOM	448	H27	Y7	25	37.082	64.683	-2.181	1.00	0.00	H
ATOM	449	H28	Y7	25	37.365	63.150	-4.120	1.00	0.00	H
ATOM	450	H29	Y7	25	38.814	64.087	-3.779	1.00	0.00	H
ATOM	451	H30	Y7	25	39.534	61.809	-4.238	1.00	0.00	H
ATOM	452	H31	Y7	25	40.683	61.269	-2.014	1.00	0.00	H
ATOM	453	H32	Y7	25	40.805	62.952	-2.499	1.00	0.00	H
ATOM	454	H33	Y7	25	38.824	61.824	-0.452	1.00	0.00	H

ATOM	455	H34	Y7	25	40.265	62.798	-0.111	1.00	0.00	H
ATOM	456	H35	Y7	25	34.217	71.605	3.394	1.00	0.00	H
ATOM	457	H36	Y7	25	34.274	72.499	5.707	1.00	0.00	H
ATOM	458	H37	Y7	25	36.410	73.339	6.673	1.00	0.00	H
ATOM	459	H38	Y7	25	38.495	73.287	5.312	1.00	0.00	H
ATOM	460	H39	Y7	25	38.447	72.406	2.991	1.00	0.00	H
ATOM	461	H40	Y7	25	42.278	66.015	-7.286	1.00	0.00	H
ATOM	462	H41	Y7	25	42.284	63.870	-8.545	1.00	0.00	H
ATOM	463	H42	Y7	25	40.289	62.378	-8.477	1.00	0.00	H
ATOM	464	H43	Y7	25	38.282	63.044	-7.152	1.00	0.00	H
ATOM	465	H44	Y7	25	38.277	65.203	-5.910	1.00	0.00	H
ATOM	466	H45	Y7	25	41.020	65.427	-1.557	1.00	0.00	H
ATOM	467	H46	Y7	25	41.314	65.316	-4.018	1.00	0.00	H
ATOM	468	H47	Y7	25	38.795	68.774	-4.476	1.00	0.00	H
ATOM	469	H48	Y7	25	36.930	70.915	-0.904	1.00	0.00	H
ATOM	470	H49	Y7	25	37.525	69.594	3.201	1.00	0.00	H
ATOM	471	H50	Y7	25	38.932	67.769	2.428	1.00	0.00	H
ATOM	472	H51	Y7	25	39.819	63.411	2.495	1.00	0.00	H
ATOM	473	H52	Y7	25	41.936	64.190	3.526	1.00	0.00	H
ATOM	474	H53	Y7	25	42.954	66.349	2.821	1.00	0.00	H
ATOM	475	H54	Y7	25	41.834	67.744	1.096	1.00	0.00	H
ATOM	476	N	NME	26	37.351	54.975	4.434	1.00	0.00	N
ATOM	477	H	NME	26	36.981	55.501	3.647	1.00	0.00	H
ATOM	478	CH3	NME	26	38.558	55.473	5.090	1.00	0.00	C
ATOM	479	HH31	NME	26	38.349	55.677	6.142	1.00	0.00	H
ATOM	480	HH32	NME	26	39.348	54.721	5.032	1.00	0.00	H
ATOM	481	HH33	NME	26	38.898	56.391	4.608	1.00	0.00	H
TER	482		NME	26						
END										

Supplementary Table 18. The list of atoms and their coordination in the structure #3646 of cR6G-P2-QSY7.

ATOM	1	H1	ACE	1	0.920	21.593	-0.933	1.00	0.00	H
ATOM	2	CH3	ACE	1	0.528	22.606	-0.851	1.00	0.00	C
ATOM	3	H2	ACE	1	1.340	23.286	-0.596	1.00	0.00	H
ATOM	4	H3	ACE	1	-0.234	22.636	-0.074	1.00	0.00	H
ATOM	5	C	ACE	1	-0.078	23.014	-2.172	1.00	0.00	C
ATOM	6	O	ACE	1	-0.070	22.232	-3.121	1.00	0.00	O
ATOM	7	N	LYS	2	-0.602	24.243	-2.243	1.00	0.00	N
ATOM	8	H	LYS	2	-0.543	24.819	-1.415	1.00	0.00	H
ATOM	9	CA	LYS	2	-1.238	24.820	-3.444	1.00	0.00	C
ATOM	10	HA	LYS	2	-1.843	24.038	-3.901	1.00	0.00	H
ATOM	11	CB	LYS	2	-2.193	25.969	-3.079	1.00	0.00	C
ATOM	12	HB2	LYS	2	-1.668	26.721	-2.486	1.00	0.00	H
ATOM	13	HB3	LYS	2	-2.518	26.444	-4.003	1.00	0.00	H
ATOM	14	CG	LYS	2	-3.427	25.467	-2.313	1.00	0.00	C
ATOM	15	HG2	LYS	2	-3.756	24.508	-2.717	1.00	0.00	H
ATOM	16	HG3	LYS	2	-3.141	25.312	-1.272	1.00	0.00	H
ATOM	17	CD	LYS	2	-4.606	26.454	-2.361	1.00	0.00	C
ATOM	18	HD2	LYS	2	-5.188	26.314	-1.449	1.00	0.00	H
ATOM	19	HD3	LYS	2	-4.241	27.483	-2.360	1.00	0.00	H
ATOM	20	CE	LYS	2	-5.560	26.222	-3.547	1.00	0.00	C
ATOM	21	HE2	LYS	2	-5.872	25.174	-3.548	1.00	0.00	H
ATOM	22	HE3	LYS	2	-6.453	26.831	-3.378	1.00	0.00	H
ATOM	23	NZ	LYS	2	-4.975	26.581	-4.863	1.00	0.00	N
ATOM	24	HZ1	LYS	2	-4.185	25.997	-5.136	1.00	0.00	H
ATOM	25	HZ2	LYS	2	-5.654	26.514	-5.606	1.00	0.00	H
ATOM	26	HZ3	LYS	2	-4.610	27.533	-4.864	1.00	0.00	H
ATOM	27	C	LYS	2	-0.230	25.225	-4.531	1.00	0.00	C
ATOM	28	O	LYS	2	-0.123	26.392	-4.896	1.00	0.00	O
ATOM	29	N	ALA	3	0.491	24.245	-5.068	1.00	0.00	N
ATOM	30	H	ALA	3	0.344	23.314	-4.696	1.00	0.00	H
ATOM	31	CA	ALA	3	1.341	24.400	-6.245	1.00	0.00	C
ATOM	32	HA	ALA	3	2.131	25.107	-6.004	1.00	0.00	H
ATOM	33	CB	ALA	3	1.979	23.044	-6.569	1.00	0.00	C
ATOM	34	HB1	ALA	3	2.638	23.147	-7.432	1.00	0.00	H
ATOM	35	HB2	ALA	3	2.565	22.697	-5.718	1.00	0.00	H
ATOM	36	HB3	ALA	3	1.207	22.307	-6.798	1.00	0.00	H
ATOM	37	C	ALA	3	0.554	24.973	-7.439	1.00	0.00	C
ATOM	38	O	ALA	3	0.990	25.926	-8.084	1.00	0.00	O
ATOM	39	N	QS	4	-0.660	24.388	-7.736	1.00	0.00	N
ATOM	40	C1	QS	4	-1.662	25.036	-8.603	1.00	0.00	C
ATOM	41	C2	QS	4	-2.493	26.094	-7.827	1.00	0.00	C
ATOM	42	C3	QS	4	-2.519	24.028	-9.419	1.00	0.00	C
ATOM	43	O1	QS	4	-3.050	25.866	-6.759	1.00	0.00	O
ATOM	44	S	QS	4	-3.768	23.090	-8.423	1.00	0.00	S
ATOM	45	C4	QS	4	0.212	15.797	-7.909	1.00	0.00	C
ATOM	46	C5	QS	4	0.065	17.145	-7.677	1.00	0.00	C
ATOM	47	C6	QS	4	-0.009	17.673	-6.338	1.00	0.00	C
ATOM	48	C7	QS	4	0.110	16.728	-5.249	1.00	0.00	C
ATOM	49	C8	QS	4	0.246	15.360	-5.443	1.00	0.00	C
ATOM	50	C9	QS	4	0.326	14.872	-6.814	1.00	0.00	C
ATOM	51	C10	QS	4	-0.195	19.019	-6.123	1.00	0.00	C
ATOM	52	N2	QS	4	-0.383	19.608	-4.873	1.00	0.00	N
ATOM	53	O2	QS	4	-0.268	19.840	-7.031	1.00	0.00	O
ATOM	54	C11	QS	4	-1.592	19.300	-4.087	1.00	0.00	C
ATOM	55	C12	QS	4	-2.705	20.360	-4.268	1.00	0.00	C
ATOM	56	N3	QS	4	-2.991	20.647	-5.691	1.00	0.00	N
ATOM	57	C13	QS	4	-2.486	21.721	-6.357	1.00	0.00	C
ATOM	58	C14	QS	4	-2.802	21.616	-7.843	1.00	0.00	C
ATOM	59	C15	QS	4	-3.627	20.302	-7.935	1.00	0.00	C
ATOM	60	C16	QS	4	-3.576	19.744	-6.526	1.00	0.00	C
ATOM	61	O3	QS	4	-3.944	18.639	-6.191	1.00	0.00	O
ATOM	62	O4	QS	4	-1.864	22.631	-5.842	1.00	0.00	O
ATOM	63	C17	QS	4	0.532	13.529	-7.123	1.00	0.00	C
ATOM	64	C18	QS	4	1.850	12.939	-6.831	1.00	0.00	C
ATOM	65	C19	QS	4	2.159	11.720	-7.455	1.00	0.00	C
ATOM	66	O5	QS	4	1.292	11.142	-8.345	1.00	0.00	O
ATOM	67	C20	QS	4	0.091	11.700	-8.698	1.00	0.00	C
ATOM	68	C21	QS	4	-0.334	12.907	-8.128	1.00	0.00	C
ATOM	69	C22	QS	4	-0.711	11.020	-9.612	1.00	0.00	C
ATOM	70	C23	QS	4	-1.974	11.512	-9.953	1.00	0.00	C
ATOM	71	C24	QS	4	-2.438	12.706	-9.359	1.00	0.00	C
ATOM	72	C25	QS	4	-1.622	13.389	-8.438	1.00	0.00	C

ATOM	73	C26	QS	4	2.829	13.541	-6.012	1.00	0.00	C
ATOM	74	C27	QS	4	4.061	12.901	-5.773	1.00	0.00	C
ATOM	75	C28	QS	4	4.331	11.653	-6.373	1.00	0.00	C
ATOM	76	C29	QS	4	3.378	11.084	-7.224	1.00	0.00	C
ATOM	77	C30	QS	4	5.085	13.578	-4.858	1.00	0.00	C
ATOM	78	C31	QS	4	-3.830	13.258	-9.674	1.00	0.00	C
ATOM	79	N4	QS	4	-2.783	10.827	-10.848	1.00	0.00	N
ATOM	80	N5	QS	4	5.546	11.023	-6.129	1.00	0.00	N
ATOM	81	C32	QS	4	-2.469	9.528	-11.462	1.00	0.00	C
ATOM	82	C33	QS	4	-3.653	9.057	-12.323	1.00	0.00	C
ATOM	83	C34	QS	4	5.933	9.672	-6.569	1.00	0.00	C
ATOM	84	C35	QS	4	6.569	9.731	-7.969	1.00	0.00	C
ATOM	85	C36	QS	4	0.223	14.505	-4.211	1.00	0.00	C
ATOM	86	O7	QS	4	0.214	14.939	-3.078	1.00	0.00	O
ATOM	87	O8	QS	4	0.101	13.192	-4.385	1.00	0.00	O
ATOM	88	H1	QS	4	-1.004	23.707	-7.061	1.00	0.00	H
ATOM	89	H2	QS	4	-1.095	25.604	-9.353	1.00	0.00	H
ATOM	90	H3	QS	4	-3.075	24.584	-10.186	1.00	0.00	H
ATOM	91	H4	QS	4	-1.864	23.321	-9.943	1.00	0.00	H
ATOM	92	H6	QS	4	0.283	15.434	-8.931	1.00	0.00	H
ATOM	93	H7	QS	4	-0.006	17.825	-8.522	1.00	0.00	H
ATOM	94	H8	QS	4	0.065	17.106	-4.231	1.00	0.00	H
ATOM	95	H9	QS	4	-0.043	20.560	-4.798	1.00	0.00	H
ATOM	96	H10	QS	4	-1.995	18.323	-4.389	1.00	0.00	H
ATOM	97	H11	QS	4	-1.324	19.235	-3.024	1.00	0.00	H
ATOM	98	H12	QS	4	-3.622	19.999	-3.783	1.00	0.00	H
ATOM	99	H13	QS	4	-2.405	21.286	-3.760	1.00	0.00	H
ATOM	100	H14	QS	4	-1.844	21.511	-8.368	1.00	0.00	H
ATOM	101	H15	QS	4	-3.182	19.578	-8.627	1.00	0.00	H
ATOM	102	H16	QS	4	-4.675	20.481	-8.203	1.00	0.00	H
ATOM	103	H17	QS	4	-0.337	10.094	-10.030	1.00	0.00	H
ATOM	104	H18	QS	4	-2.007	14.281	-7.947	1.00	0.00	H
ATOM	105	H19	QS	4	2.652	14.509	-5.554	1.00	0.00	H
ATOM	106	H20	QS	4	3.566	10.146	-7.730	1.00	0.00	H
ATOM	107	H21	QS	4	4.719	14.538	-4.473	1.00	0.00	H
ATOM	108	H22	QS	4	5.317	12.948	-3.990	1.00	0.00	H
ATOM	109	H23	QS	4	6.021	13.779	-5.395	1.00	0.00	H
ATOM	110	H24	QS	4	-4.613	12.544	-9.387	1.00	0.00	H
ATOM	111	H25	QS	4	-4.031	14.193	-9.135	1.00	0.00	H
ATOM	112	H26	QS	4	-3.939	13.471	-10.746	1.00	0.00	H
ATOM	113	H27	QS	4	-3.695	11.221	-11.029	1.00	0.00	H
ATOM	114	H28	QS	4	6.186	11.510	-5.516	1.00	0.00	H
ATOM	115	H29	QS	4	-1.574	9.614	-12.094	1.00	0.00	H
ATOM	116	H30	QS	4	-2.269	8.779	-10.684	1.00	0.00	H
ATOM	117	H31	QS	4	-3.865	9.774	-13.126	1.00	0.00	H
ATOM	118	H32	QS	4	-3.427	8.088	-12.785	1.00	0.00	H
ATOM	119	H33	QS	4	-4.558	8.938	-11.713	1.00	0.00	H
ATOM	120	H34	QS	4	6.664	9.250	-5.865	1.00	0.00	H
ATOM	121	H35	QS	4	5.067	8.997	-6.577	1.00	0.00	H
ATOM	122	H36	QS	4	5.866	10.137	-8.706	1.00	0.00	H
ATOM	123	H37	QS	4	7.465	10.364	-7.964	1.00	0.00	H
ATOM	124	H38	QS	4	6.865	8.726	-8.298	1.00	0.00	H
ATOM	125	H40	QS	4	0.033	12.916	-3.454	1.00	0.00	H
ATOM	126	N	ALA	5	-2.535	27.346	-8.410	1.00	0.00	N
ATOM	127	H	ALA	5	-2.050	27.470	-9.285	1.00	0.00	H
ATOM	128	CA	ALA	5	-3.143	28.542	-7.819	1.00	0.00	C
ATOM	129	HA	ALA	5	-2.832	29.398	-8.414	1.00	0.00	H
ATOM	130	CB	ALA	5	-4.669	28.456	-7.993	1.00	0.00	C
ATOM	131	HB1	ALA	5	-4.915	28.380	-9.053	1.00	0.00	H
ATOM	132	HB2	ALA	5	-5.067	27.584	-7.477	1.00	0.00	H
ATOM	133	HB3	ALA	5	-5.137	29.357	-7.593	1.00	0.00	H
ATOM	134	C	ALA	5	-2.679	28.836	-6.364	1.00	0.00	C
ATOM	135	O	ALA	5	-3.498	28.817	-5.444	1.00	0.00	O
ATOM	136	N	ALA	6	-1.396	29.085	-6.076	1.00	0.00	N
ATOM	137	H	ALA	6	-1.205	29.268	-5.101	1.00	0.00	H
ATOM	138	CA	ALA	6	-0.196	29.000	-6.920	1.00	0.00	C
ATOM	139	HA	ALA	6	-0.218	28.085	-7.509	1.00	0.00	H
ATOM	140	CB	ALA	6	-0.108	30.217	-7.857	1.00	0.00	C
ATOM	141	HB1	ALA	6	-0.136	31.139	-7.274	1.00	0.00	H
ATOM	142	HB2	ALA	6	0.832	30.186	-8.411	1.00	0.00	H
ATOM	143	HB3	ALA	6	-0.920	30.225	-8.579	1.00	0.00	H
ATOM	144	C	ALA	6	1.073	28.965	-6.044	1.00	0.00	C
ATOM	145	O	ALA	6	1.112	29.595	-4.985	1.00	0.00	O
ATOM	146	N	ALA	7	2.145	28.329	-6.530	1.00	0.00	N
ATOM	147	H	ALA	7	2.025	27.773	-7.366	1.00	0.00	H
ATOM	148	CA	ALA	7	3.499	28.425	-5.967	1.00	0.00	C

ATOM	149	HA	ALA	7	3.429	28.293	-4.886	1.00	0.00	H
ATOM	150	CB	ALA	7	4.342	27.269	-6.529	1.00	0.00	C
ATOM	151	HB1	ALA	7	5.349	27.312	-6.112	1.00	0.00	H
ATOM	152	HB2	ALA	7	3.899	26.314	-6.258	1.00	0.00	H
ATOM	153	HB3	ALA	7	4.405	27.345	-7.616	1.00	0.00	H
ATOM	154	C	ALA	7	4.151	29.812	-6.211	1.00	0.00	C
ATOM	155	O	ALA	7	5.209	29.913	-6.840	1.00	0.00	O
ATOM	156	N	HIE	8	3.500	30.893	-5.755	1.00	0.00	N
ATOM	157	H	HIE	8	2.632	30.716	-5.258	1.00	0.00	H
ATOM	158	CA	HIE	8	3.813	32.289	-6.116	1.00	0.00	C
ATOM	159	HA	HIE	8	3.009	32.924	-5.744	1.00	0.00	H
ATOM	160	CB	HIE	8	5.105	32.700	-5.365	1.00	0.00	C
ATOM	161	HB2	HIE	8	4.989	32.450	-4.310	1.00	0.00	H
ATOM	162	HB3	HIE	8	5.942	32.118	-5.746	1.00	0.00	H
ATOM	163	CG	HIE	8	5.483	34.158	-5.448	1.00	0.00	C
ATOM	164	ND1	HIE	8	6.409	34.697	-6.345	1.00	0.00	N
ATOM	165	CE1	HIE	8	6.391	36.024	-6.138	1.00	0.00	C
ATOM	166	HE1	HIE	8	6.986	36.744	-6.686	1.00	0.00	H
ATOM	167	NE2	HIE	8	5.512	36.335	-5.170	1.00	0.00	N
ATOM	168	HE2	HIE	8	5.313	37.271	-4.840	1.00	0.00	H
ATOM	169	CD2	HIE	8	4.926	35.171	-4.725	1.00	0.00	C
ATOM	170	HD2	HIE	8	4.164	35.073	-3.963	1.00	0.00	H
ATOM	171	C	HIE	8	3.817	32.472	-7.655	1.00	0.00	C
ATOM	172	O	HIE	8	3.227	31.669	-8.380	1.00	0.00	O
ATOM	173	N	ALA	9	4.477	33.501	-8.188	1.00	0.00	N
ATOM	174	H	ALA	9	4.952	34.136	-7.558	1.00	0.00	H
ATOM	175	CA	ALA	9	4.824	33.613	-9.605	1.00	0.00	C
ATOM	176	HA	ALA	9	3.952	33.352	-10.208	1.00	0.00	H
ATOM	177	CB	ALA	9	5.184	35.078	-9.894	1.00	0.00	C
ATOM	178	HB1	ALA	9	6.042	35.383	-9.292	1.00	0.00	H
ATOM	179	HB2	ALA	9	5.431	35.195	-10.950	1.00	0.00	H
ATOM	180	HB3	ALA	9	4.335	35.722	-9.658	1.00	0.00	H
ATOM	181	C	ALA	9	5.955	32.619	-9.959	1.00	0.00	C
ATOM	182	O	ALA	9	7.082	33.018	-10.262	1.00	0.00	O
ATOM	183	N	ALA	10	5.693	31.315	-9.805	1.00	0.00	N
ATOM	184	H	ALA	10	4.758	31.069	-9.494	1.00	0.00	H
ATOM	185	CA	ALA	10	6.690	30.241	-9.873	1.00	0.00	C
ATOM	186	HA	ALA	10	6.248	29.357	-9.410	1.00	0.00	H
ATOM	187	CB	ALA	10	6.972	29.897	-11.344	1.00	0.00	C
ATOM	188	HB1	ALA	10	6.043	29.616	-11.843	1.00	0.00	H
ATOM	189	HB2	ALA	10	7.407	30.756	-11.856	1.00	0.00	H
ATOM	190	HB3	ALA	10	7.667	29.058	-11.398	1.00	0.00	H
ATOM	191	C	ALA	10	7.948	30.583	-9.042	1.00	0.00	C
ATOM	192	O	ALA	10	9.055	30.678	-9.574	1.00	0.00	O
ATOM	193	N	ALA	11	7.746	30.897	-7.759	1.00	0.00	N
ATOM	194	H	ALA	11	6.808	30.743	-7.400	1.00	0.00	H
ATOM	195	CA	ALA	11	8.761	31.328	-6.790	1.00	0.00	C
ATOM	196	HA	ALA	11	8.215	31.626	-5.895	1.00	0.00	H
ATOM	197	CB	ALA	11	9.610	30.112	-6.386	1.00	0.00	C
ATOM	198	HB1	ALA	11	8.963	29.290	-6.078	1.00	0.00	H
ATOM	199	HB2	ALA	11	10.228	29.791	-7.225	1.00	0.00	H
ATOM	200	HB3	ALA	11	10.260	30.376	-5.551	1.00	0.00	H
ATOM	201	C	ALA	11	9.603	32.578	-7.162	1.00	0.00	C
ATOM	202	O	ALA	11	10.628	32.825	-6.528	1.00	0.00	O
ATOM	203	N	HIP	12	9.203	33.399	-8.148	1.00	0.00	N
ATOM	204	H	HIP	12	8.336	33.187	-8.628	1.00	0.00	H
ATOM	205	CA	HIP	12	9.914	34.642	-8.521	1.00	0.00	C
ATOM	206	HA	HIP	12	10.987	34.445	-8.468	1.00	0.00	H
ATOM	207	CB	HIP	12	9.604	35.049	-9.978	1.00	0.00	C
ATOM	208	HB2	HIP	12	8.525	35.134	-10.113	1.00	0.00	H
ATOM	209	HB3	HIP	12	10.022	36.043	-10.151	1.00	0.00	H
ATOM	210	CG	HIP	12	10.177	34.128	-11.033	1.00	0.00	C
ATOM	211	ND1	HIP	12	9.994	32.762	-11.131	1.00	0.00	N
ATOM	212	HD1	HIP	12	9.443	32.184	-10.490	1.00	0.00	H
ATOM	213	CE1	HIP	12	10.681	32.313	-12.198	1.00	0.00	C
ATOM	214	HE1	HIP	12	10.733	31.275	-12.513	1.00	0.00	H
ATOM	215	NE2	HIP	12	11.292	33.362	-12.790	1.00	0.00	N
ATOM	216	HE2	HIP	12	11.880	33.321	-13.617	1.00	0.00	H
ATOM	217	CD2	HIP	12	10.986	34.506	-12.071	1.00	0.00	C
ATOM	218	HD2	HIP	12	11.321	35.516	-12.279	1.00	0.00	H
ATOM	219	C	HIP	12	9.670	35.791	-7.521	1.00	0.00	C
ATOM	220	O	HIP	12	9.124	36.835	-7.878	1.00	0.00	O
ATOM	221	N	ALA	13	10.056	35.596	-6.262	1.00	0.00	N
ATOM	222	H	ALA	13	10.489	34.706	-6.046	1.00	0.00	H
ATOM	223	CA	ALA	13	10.144	36.658	-5.260	1.00	0.00	C
ATOM	224	HA	ALA	13	9.233	37.256	-5.301	1.00	0.00	H

ATOM	225	CB	ALA	13	10.228	36.005	-3.874	1.00	0.00	C
ATOM	226	HB1	ALA	13	11.136	35.404	-3.801	1.00	0.00	H
ATOM	227	HB2	ALA	13	10.246	36.773	-3.100	1.00	0.00	H
ATOM	228	HB3	ALA	13	9.360	35.365	-3.714	1.00	0.00	H
ATOM	229	C	ALA	13	11.338	37.601	-5.531	1.00	0.00	C
ATOM	230	O	ALA	13	12.267	37.251	-6.259	1.00	0.00	O
ATOM	231	N	ALA	14	11.326	38.790	-4.923	1.00	0.00	N
ATOM	232	H	ALA	14	10.561	39.015	-4.302	1.00	0.00	H
ATOM	233	CA	ALA	14	12.389	39.793	-5.020	1.00	0.00	C
ATOM	234	HA	ALA	14	13.351	39.284	-5.103	1.00	0.00	H
ATOM	235	CB	ALA	14	12.168	40.629	-6.290	1.00	0.00	C
ATOM	236	HB1	ALA	14	12.171	39.980	-7.166	1.00	0.00	H
ATOM	237	HB2	ALA	14	11.211	41.149	-6.232	1.00	0.00	H
ATOM	238	HB3	ALA	14	12.968	41.363	-6.397	1.00	0.00	H
ATOM	239	C	ALA	14	12.425	40.677	-3.759	1.00	0.00	C
ATOM	240	O	ALA	14	11.445	40.740	-3.018	1.00	0.00	O
ATOM	241	N	ALA	15	13.543	41.371	-3.525	1.00	0.00	N
ATOM	242	H	ALA	15	14.304	41.305	-4.185	1.00	0.00	H
ATOM	243	CA	ALA	15	13.734	42.282	-2.394	1.00	0.00	C
ATOM	244	HA	ALA	15	12.778	42.752	-2.155	1.00	0.00	H
ATOM	245	CB	ALA	15	14.193	41.474	-1.172	1.00	0.00	C
ATOM	246	HB1	ALA	15	15.143	40.982	-1.383	1.00	0.00	H
ATOM	247	HB2	ALA	15	14.316	42.134	-0.313	1.00	0.00	H
ATOM	248	HB3	ALA	15	13.445	40.718	-0.925	1.00	0.00	H
ATOM	249	C	ALA	15	14.738	43.399	-2.736	1.00	0.00	C
ATOM	250	O	ALA	15	15.603	43.230	-3.598	1.00	0.00	O
ATOM	251	N	ALA	16	14.642	44.543	-2.051	1.00	0.00	N
ATOM	252	H	ALA	16	13.939	44.623	-1.333	1.00	0.00	H
ATOM	253	CA	ALA	16	15.558	45.667	-2.245	1.00	0.00	C
ATOM	254	HA	ALA	16	15.592	45.900	-3.311	1.00	0.00	H
ATOM	255	CB	ALA	16	14.999	46.892	-1.510	1.00	0.00	C
ATOM	256	HB1	ALA	16	14.943	46.692	-0.438	1.00	0.00	H
ATOM	257	HB2	ALA	16	15.649	47.751	-1.675	1.00	0.00	H
ATOM	258	HB3	ALA	16	14.002	47.129	-1.884	1.00	0.00	H
ATOM	259	C	ALA	16	16.987	45.325	-1.776	1.00	0.00	C
ATOM	260	O	ALA	16	17.195	44.869	-0.652	1.00	0.00	O
ATOM	261	N	HIP	17	17.997	45.578	-2.620	1.00	0.00	N
ATOM	262	H	HIP	17	17.769	45.949	-3.530	1.00	0.00	H
ATOM	263	CA	HIP	17	19.409	45.326	-2.290	1.00	0.00	C
ATOM	264	HA	HIP	17	19.459	44.379	-1.751	1.00	0.00	H
ATOM	265	CB	HIP	17	20.233	45.169	-3.583	1.00	0.00	C
ATOM	266	HB2	HIP	17	19.766	44.415	-4.219	1.00	0.00	H
ATOM	267	HB3	HIP	17	20.235	46.114	-4.131	1.00	0.00	H
ATOM	268	CG	HIP	17	21.660	44.755	-3.307	1.00	0.00	C
ATOM	269	ND1	HIP	17	22.093	43.489	-2.949	1.00	0.00	N
ATOM	270	HD1	HIP	17	21.517	42.655	-2.923	1.00	0.00	H
ATOM	271	CE1	HIP	17	23.397	43.574	-2.608	1.00	0.00	C
ATOM	272	HE1	HIP	17	24.014	42.756	-2.247	1.00	0.00	H
ATOM	273	NE2	HIP	17	23.796	44.857	-2.738	1.00	0.00	N
ATOM	274	HE2	HIP	17	24.705	45.239	-2.450	1.00	0.00	H
ATOM	275	CD2	HIP	17	22.722	45.607	-3.176	1.00	0.00	C
ATOM	276	HD2	HIP	17	22.690	46.686	-3.293	1.00	0.00	H
ATOM	277	C	HIP	17	19.981	46.395	-1.336	1.00	0.00	C
ATOM	278	O	HIP	17	20.734	47.274	-1.751	1.00	0.00	O
ATOM	279	N	ALA	18	19.618	46.327	-0.058	1.00	0.00	N
ATOM	280	H	ALA	18	18.929	45.633	0.206	1.00	0.00	H
ATOM	281	CA	ALA	18	20.163	47.199	0.980	1.00	0.00	C
ATOM	282	HA	ALA	18	20.058	48.233	0.646	1.00	0.00	H
ATOM	283	CB	ALA	18	19.319	47.025	2.249	1.00	0.00	C
ATOM	284	HB1	ALA	18	19.669	47.706	3.026	1.00	0.00	H
ATOM	285	HB2	ALA	18	18.273	47.246	2.034	1.00	0.00	H
ATOM	286	HB3	ALA	18	19.401	45.999	2.615	1.00	0.00	H
ATOM	287	C	ALA	18	21.662	46.938	1.257	1.00	0.00	C
ATOM	288	O	ALA	18	22.139	45.809	1.134	1.00	0.00	O
ATOM	289	N	ALA	19	22.369	47.993	1.687	1.00	0.00	N
ATOM	290	H	ALA	19	21.869	48.868	1.708	1.00	0.00	H
ATOM	291	CA	ALA	19	23.704	48.030	2.309	1.00	0.00	C
ATOM	292	HA	ALA	19	23.962	49.087	2.400	1.00	0.00	H
ATOM	293	CB	ALA	19	23.583	47.502	3.746	1.00	0.00	C
ATOM	294	HB1	ALA	19	22.818	48.061	4.287	1.00	0.00	H
ATOM	295	HB2	ALA	19	23.316	46.444	3.741	1.00	0.00	H
ATOM	296	HB3	ALA	19	24.535	47.624	4.265	1.00	0.00	H
ATOM	297	C	ALA	19	24.906	47.397	1.566	1.00	0.00	C
ATOM	298	O	ALA	19	25.909	48.076	1.346	1.00	0.00	O
ATOM	299	N	ALA	20	24.867	46.105	1.235	1.00	0.00	N
ATOM	300	H	ALA	20	23.986	45.621	1.371	1.00	0.00	H

ATOM	301	CA	ALA	20	26.049	45.319	0.874	1.00	0.00	C
ATOM	302	HA	ALA	20	26.778	45.431	1.679	1.00	0.00	H
ATOM	303	CB	ALA	20	25.646	43.840	0.821	1.00	0.00	C
ATOM	304	HB1	ALA	20	25.254	43.530	1.791	1.00	0.00	H
ATOM	305	HB2	ALA	20	24.879	43.687	0.062	1.00	0.00	H
ATOM	306	HB3	ALA	20	26.517	43.228	0.583	1.00	0.00	H
ATOM	307	C	ALA	20	26.721	45.779	-0.438	1.00	0.00	C
ATOM	308	O	ALA	20	26.089	45.801	-1.499	1.00	0.00	O
ATOM	309	N	HIP	21	28.026	46.071	-0.377	1.00	0.00	N
ATOM	310	H	HIP	21	28.488	45.983	0.520	1.00	0.00	H
ATOM	311	CA	HIP	21	28.876	46.482	-1.506	1.00	0.00	C
ATOM	312	HA	HIP	21	28.469	46.057	-2.424	1.00	0.00	H
ATOM	313	CB	HIP	21	28.848	48.021	-1.632	1.00	0.00	C
ATOM	314	HB2	HIP	21	29.417	48.317	-2.515	1.00	0.00	H
ATOM	315	HB3	HIP	21	27.816	48.334	-1.806	1.00	0.00	H
ATOM	316	CG	HIP	21	29.387	48.797	-0.445	1.00	0.00	C
ATOM	317	ND1	HIP	21	30.717	48.955	-0.092	1.00	0.00	N
ATOM	318	HD1	HIP	21	31.538	48.570	-0.567	1.00	0.00	H
ATOM	319	CE1	HIP	21	30.775	49.749	0.989	1.00	0.00	C
ATOM	320	HE1	HIP	21	31.693	50.082	1.459	1.00	0.00	H
ATOM	321	NE2	HIP	21	29.526	50.100	1.346	1.00	0.00	N
ATOM	322	HE2	HIP	21	29.289	50.720	2.117	1.00	0.00	H
ATOM	323	CD2	HIP	21	28.642	49.513	0.455	1.00	0.00	C
ATOM	324	HD2	HIP	21	27.562	49.612	0.456	1.00	0.00	H
ATOM	325	C	HIP	21	30.301	45.928	-1.344	1.00	0.00	C
ATOM	326	O	HIP	21	30.676	45.530	-0.245	1.00	0.00	O
ATOM	327	N	ALA	22	31.098	45.928	-2.418	1.00	0.00	N
ATOM	328	H	ALA	22	30.732	46.265	-3.292	1.00	0.00	H
ATOM	329	CA	ALA	22	32.519	45.555	-2.373	1.00	0.00	C
ATOM	330	HA	ALA	22	32.596	44.529	-2.006	1.00	0.00	H
ATOM	331	CB	ALA	22	33.075	45.598	-3.803	1.00	0.00	C
ATOM	332	HB1	ALA	22	32.500	44.933	-4.448	1.00	0.00	H
ATOM	333	HB2	ALA	22	33.024	46.615	-4.196	1.00	0.00	H
ATOM	334	HB3	ALA	22	34.115	45.270	-3.809	1.00	0.00	H
ATOM	335	C	ALA	22	33.331	46.456	-1.414	1.00	0.00	C
ATOM	336	O	ALA	22	32.868	47.536	-1.051	1.00	0.00	O
ATOM	337	N	ALA	23	34.537	46.030	-1.021	1.00	0.00	N
ATOM	338	H	ALA	23	34.872	45.157	-1.399	1.00	0.00	H
ATOM	339	CA	ALA	23	35.298	46.574	0.118	1.00	0.00	C
ATOM	340	HA	ALA	23	34.698	46.426	1.017	1.00	0.00	H
ATOM	341	CB	ALA	23	36.568	45.727	0.265	1.00	0.00	C
ATOM	342	HB1	ALA	23	36.309	44.674	0.386	1.00	0.00	H
ATOM	343	HB2	ALA	23	37.206	45.846	-0.613	1.00	0.00	H
ATOM	344	HB3	ALA	23	37.123	46.049	1.148	1.00	0.00	H
ATOM	345	C	ALA	23	35.647	48.083	0.083	1.00	0.00	C
ATOM	346	O	ALA	23	36.024	48.636	1.115	1.00	0.00	O
ATOM	347	N	ALA	24	35.491	48.765	-1.058	1.00	0.00	N
ATOM	348	H	ALA	24	35.132	48.270	-1.856	1.00	0.00	H
ATOM	349	CA	ALA	24	35.593	50.221	-1.156	1.00	0.00	C
ATOM	350	HA	ALA	24	36.619	50.511	-0.920	1.00	0.00	H
ATOM	351	CB	ALA	24	35.295	50.642	-2.600	1.00	0.00	C
ATOM	352	HB1	ALA	24	36.002	50.167	-3.280	1.00	0.00	H
ATOM	353	HB2	ALA	24	34.278	50.354	-2.871	1.00	0.00	H
ATOM	354	HB3	ALA	24	35.392	51.725	-2.691	1.00	0.00	H
ATOM	355	C	ALA	24	34.653	50.911	-0.146	1.00	0.00	C
ATOM	356	O	ALA	24	33.437	50.708	-0.164	1.00	0.00	O
ATOM	357	N	Y7	25	35.247	51.716	0.798	1.00	0.00	N
ATOM	358	C1	Y7	25	34.584	52.306	1.982	1.00	0.00	C
ATOM	359	C2	Y7	25	33.668	51.300	2.733	1.00	0.00	C
ATOM	360	C3	Y7	25	33.870	53.635	1.620	1.00	0.00	C
ATOM	361	O1	Y7	25	32.516	51.558	3.046	1.00	0.00	O
ATOM	362	S	Y7	25	34.946	54.815	0.672	1.00	0.00	S
ATOM	363	C4	Y7	25	52.167	63.335	0.146	1.00	0.00	C
ATOM	364	C5	Y7	25	44.550	67.659	-3.887	1.00	0.00	C
ATOM	365	O2	Y7	25	37.662	53.077	1.156	1.00	0.00	O
ATOM	366	O3	Y7	25	39.118	57.365	1.377	1.00	0.00	O
ATOM	367	C6	Y7	25	38.347	56.432	1.455	1.00	0.00	C
ATOM	368	C7	Y7	25	36.873	56.539	1.798	1.00	0.00	C
ATOM	369	C8	Y7	25	36.383	55.064	1.817	1.00	0.00	C
ATOM	370	C9	Y7	25	37.620	54.276	1.369	1.00	0.00	C
ATOM	371	N2	Y7	25	38.675	55.126	1.234	1.00	0.00	N
ATOM	372	C10	Y7	25	40.022	54.686	0.821	1.00	0.00	C
ATOM	373	C11	Y7	25	40.171	54.576	-0.716	1.00	0.00	C
ATOM	374	C12	Y7	25	40.075	55.945	-1.437	1.00	0.00	C
ATOM	375	C13	Y7	25	40.360	55.828	-2.951	1.00	0.00	C
ATOM	376	C14	Y7	25	40.251	57.193	-3.662	1.00	0.00	C

ATOM	377	N3	Y7	25	40.488	57.053	-5.106	1.00	0.00	N
ATOM	378	O4	Y7	25	41.563	57.175	-7.024	1.00	0.00	O
ATOM	379	C15	Y7	25	41.569	57.467	-5.848	1.00	0.00	C
ATOM	380	C16	Y7	25	44.795	58.365	-3.749	1.00	0.00	C
ATOM	381	C17	Y7	25	43.637	57.507	-4.311	1.00	0.00	C
ATOM	382	C18	Y7	25	42.673	58.338	-5.207	1.00	0.00	C
ATOM	383	C19	Y7	25	43.504	59.088	-6.288	1.00	0.00	C
ATOM	384	C20	Y7	25	44.651	59.912	-5.669	1.00	0.00	C
ATOM	385	N4	Y7	25	45.519	59.061	-4.830	1.00	0.00	N
ATOM	386	C21	Y7	25	51.876	59.808	-0.060	1.00	0.00	C
ATOM	387	C22	Y7	25	52.538	58.632	0.330	1.00	0.00	C
ATOM	388	C23	Y7	25	53.935	58.623	0.461	1.00	0.00	C
ATOM	389	C24	Y7	25	54.669	59.790	0.201	1.00	0.00	C
ATOM	390	C25	Y7	25	54.007	60.966	-0.188	1.00	0.00	C
ATOM	391	C26	Y7	25	42.945	67.646	-6.465	1.00	0.00	C
ATOM	392	C27	Y7	25	42.399	68.184	-7.643	1.00	0.00	C
ATOM	393	C28	Y7	25	43.245	68.594	-8.686	1.00	0.00	C
ATOM	394	C29	Y7	25	44.636	68.460	-8.552	1.00	0.00	C
ATOM	395	C30	Y7	25	45.178	67.920	-7.374	1.00	0.00	C
ATOM	396	C31	Y7	25	44.339	67.515	-6.321	1.00	0.00	C
ATOM	397	C32	Y7	25	52.604	60.985	-0.319	1.00	0.00	C
ATOM	398	N5	Y7	25	51.939	62.150	-0.714	1.00	0.00	N
ATOM	399	N6	Y7	25	44.891	66.977	-5.159	1.00	0.00	N
ATOM	400	C33	Y7	25	46.589	63.828	-6.158	1.00	0.00	C
ATOM	401	C34	Y7	25	45.724	64.931	-6.160	1.00	0.00	C
ATOM	402	C35	Y7	25	45.766	65.886	-5.132	1.00	0.00	C
ATOM	403	C36	Y7	25	46.698	65.715	-4.097	1.00	0.00	C
ATOM	404	C37	Y7	25	50.148	63.361	-1.928	1.00	0.00	C
ATOM	405	C38	Y7	25	51.063	62.262	-1.794	1.00	0.00	C
ATOM	406	C39	Y7	25	51.067	61.261	-2.825	1.00	0.00	C
ATOM	407	C40	Y7	25	50.218	61.330	-3.904	1.00	0.00	C
ATOM	408	C41	Y7	25	47.521	63.670	-5.116	1.00	0.00	C
ATOM	409	C42	Y7	25	47.561	64.616	-4.089	1.00	0.00	C
ATOM	410	O5	Y7	25	48.450	64.514	-3.053	1.00	0.00	O
ATOM	411	C43	Y7	25	49.294	63.443	-2.995	1.00	0.00	C
ATOM	412	C44	Y7	25	49.298	62.433	-4.015	1.00	0.00	C
ATOM	413	C45	Y7	25	48.416	62.521	-5.081	1.00	0.00	C
ATOM	414	C46	Y7	25	48.410	61.440	-6.073	1.00	0.00	C
ATOM	415	C47	Y7	25	47.775	60.200	-5.823	1.00	0.00	C
ATOM	416	C48	Y7	25	47.856	59.168	-6.781	1.00	0.00	C
ATOM	417	C49	Y7	25	48.530	59.379	-7.993	1.00	0.00	C
ATOM	418	C50	Y7	25	49.138	60.615	-8.253	1.00	0.00	C
ATOM	419	C51	Y7	25	49.080	61.638	-7.294	1.00	0.00	C
ATOM	420	S1	Y7	25	46.895	59.862	-4.280	1.00	0.00	S
ATOM	421	O7	Y7	25	46.421	61.077	-3.621	1.00	0.00	O
ATOM	422	O8	Y7	25	47.676	58.846	-3.580	1.00	0.00	O
ATOM	423	H	Y7	25	36.252	51.881	0.729	1.00	0.00	H
ATOM	424	H1	Y7	25	35.378	52.540	2.701	1.00	0.00	H
ATOM	425	H2	Y7	25	32.992	53.409	1.002	1.00	0.00	H
ATOM	426	H3	Y7	25	33.515	54.126	2.536	1.00	0.00	H
ATOM	427	H6	Y7	25	51.258	63.579	0.710	1.00	0.00	H
ATOM	428	H7	Y7	25	52.966	63.146	0.875	1.00	0.00	H
ATOM	429	H8	Y7	25	52.463	64.200	-0.462	1.00	0.00	H
ATOM	430	H9	Y7	25	43.683	68.320	-4.013	1.00	0.00	H
ATOM	431	H10	Y7	25	44.297	66.922	-3.113	1.00	0.00	H
ATOM	432	H11	Y7	25	45.392	68.274	-3.545	1.00	0.00	H
ATOM	433	H12	Y7	25	36.759	57.028	2.772	1.00	0.00	H
ATOM	434	H13	Y7	25	36.375	57.135	1.024	1.00	0.00	H
ATOM	435	H14	Y7	25	36.136	54.758	2.840	1.00	0.00	H
ATOM	436	H15	Y7	25	40.773	55.386	1.213	1.00	0.00	H
ATOM	437	H16	Y7	25	40.227	53.704	1.270	1.00	0.00	H
ATOM	438	H17	Y7	25	41.145	54.121	-0.937	1.00	0.00	H
ATOM	439	H18	Y7	25	39.408	53.892	-1.110	1.00	0.00	H
ATOM	440	H19	Y7	25	39.073	56.369	-1.292	1.00	0.00	H
ATOM	441	H20	Y7	25	40.788	56.647	-0.984	1.00	0.00	H
ATOM	442	H21	Y7	25	41.362	55.409	-3.109	1.00	0.00	H
ATOM	443	H22	Y7	25	39.647	55.126	-3.403	1.00	0.00	H
ATOM	444	H23	Y7	25	39.240	57.597	-3.515	1.00	0.00	H
ATOM	445	H24	Y7	25	40.955	57.903	-3.210	1.00	0.00	H
ATOM	446	H25	Y7	25	39.821	56.538	-5.663	1.00	0.00	H
ATOM	447	H26	Y7	25	45.475	57.704	-3.195	1.00	0.00	H
ATOM	448	H27	Y7	25	44.391	59.097	-3.035	1.00	0.00	H
ATOM	449	H28	Y7	25	44.063	56.686	-4.904	1.00	0.00	H
ATOM	450	H29	Y7	25	43.097	57.047	-3.477	1.00	0.00	H
ATOM	451	H30	Y7	25	42.177	59.096	-4.590	1.00	0.00	H
ATOM	452	H31	Y7	25	43.934	58.363	-6.992	1.00	0.00	H

```
ATOM     453   H32   Y7     25          42.850   59.747   -6.874   1.00   0.00                 H
ATOM     454   H33   Y7     25          45.228   60.368   -6.484   1.00   0.00                 H
ATOM     455   H34   Y7     25          44.225   60.727   -5.068   1.00   0.00                 H
ATOM     456   H35   Y7     25          50.792   59.805   -0.152   1.00   0.00                 H
ATOM     457   H36   Y7     25          51.968   57.728    0.533   1.00   0.00                 H
ATOM     458   H37   Y7     25          54.449   57.713    0.764   1.00   0.00                 H
ATOM     459   H38   Y7     25          55.753   59.783    0.300   1.00   0.00                 H
ATOM     460   H39   Y7     25          54.590   61.859   -0.398   1.00   0.00                 H
ATOM     461   H40   Y7     25          42.284   67.319   -5.669   1.00   0.00                 H
ATOM     462   H41   Y7     25          41.321   68.286   -7.747   1.00   0.00                 H
ATOM     463   H42   Y7     25          42.824   69.016   -9.596   1.00   0.00                 H
ATOM     464   H43   Y7     25          45.293   68.782   -9.357   1.00   0.00                 H
ATOM     465   H44   Y7     25          46.256   67.827   -7.269   1.00   0.00                 H
ATOM     466   H45   Y7     25          46.528   63.099   -6.964   1.00   0.00                 H
ATOM     467   H46   Y7     25          45.003   65.043   -6.965   1.00   0.00                 H
ATOM     468   H47   Y7     25          46.783   66.431   -3.291   1.00   0.00                 H
ATOM     469   H48   Y7     25          50.101   64.140   -1.181   1.00   0.00                 H
ATOM     470   H49   Y7     25          51.761   60.431   -2.753   1.00   0.00                 H
ATOM     471   H50   Y7     25          50.260   60.543   -4.653   1.00   0.00                 H
ATOM     472   H51   Y7     25          47.393   58.201   -6.582   1.00   0.00                 H
ATOM     473   H52   Y7     25          48.586   58.579   -8.729   1.00   0.00                 H
ATOM     474   H53   Y7     25          49.666   60.776   -9.191   1.00   0.00                 H
ATOM     475   H54   Y7     25          49.573   62.588   -7.491   1.00   0.00                 H
ATOM     476   N     NME     26          34.212   50.055    3.001   1.00   0.00                 N
ATOM     477   H     NME     26          35.127   49.849    2.611   1.00   0.00                 H
ATOM     478   CH3 NME       26          33.562   49.006    3.783   1.00   0.00                 C
ATOM     479 HH31 NME       26          34.318   48.338    4.201   1.00   0.00                 H
ATOM     480 HH32 NME       26          32.991   49.447    4.603   1.00   0.00                 H
ATOM     481 HH33 NME       26          32.891   48.428    3.147   1.00   0.00                 H
TER     482          NME       26
END
```

Supplementary Note 5.8. DMS separations

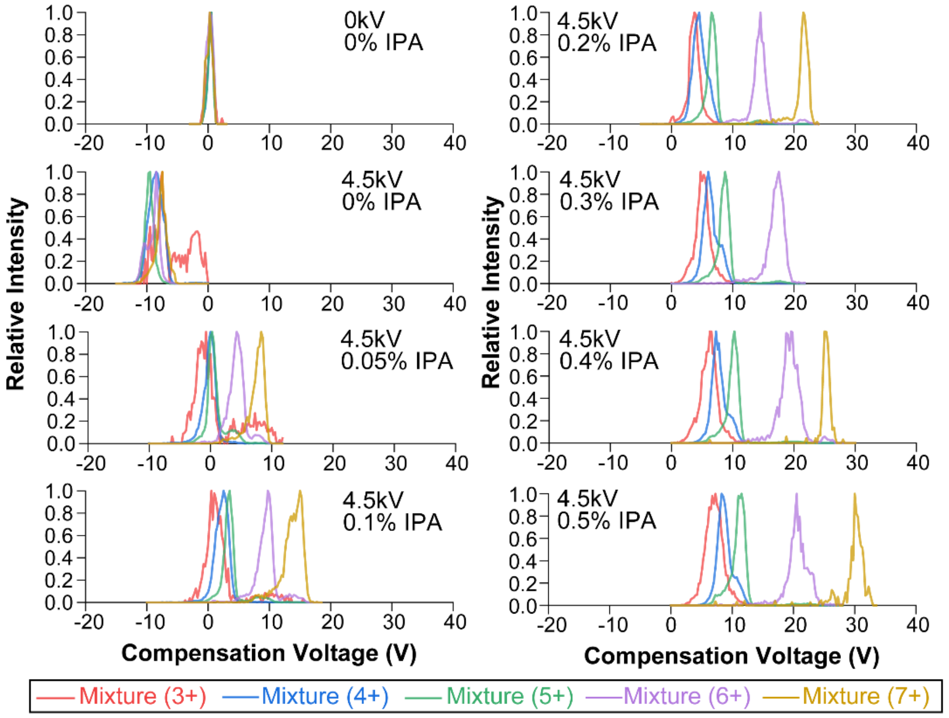

Supplementary Figure 34. Normalized DMS ionograms of the protonated ions of cR6G-**P1**-QSY7 and cR6G-**P2**-QSY7 mixture ([M+nH]$^{n+}$, n=3 to 7) in 0 to 0.5 mol % IPA concentration. DV was set to 4.5 kV for all experiments.

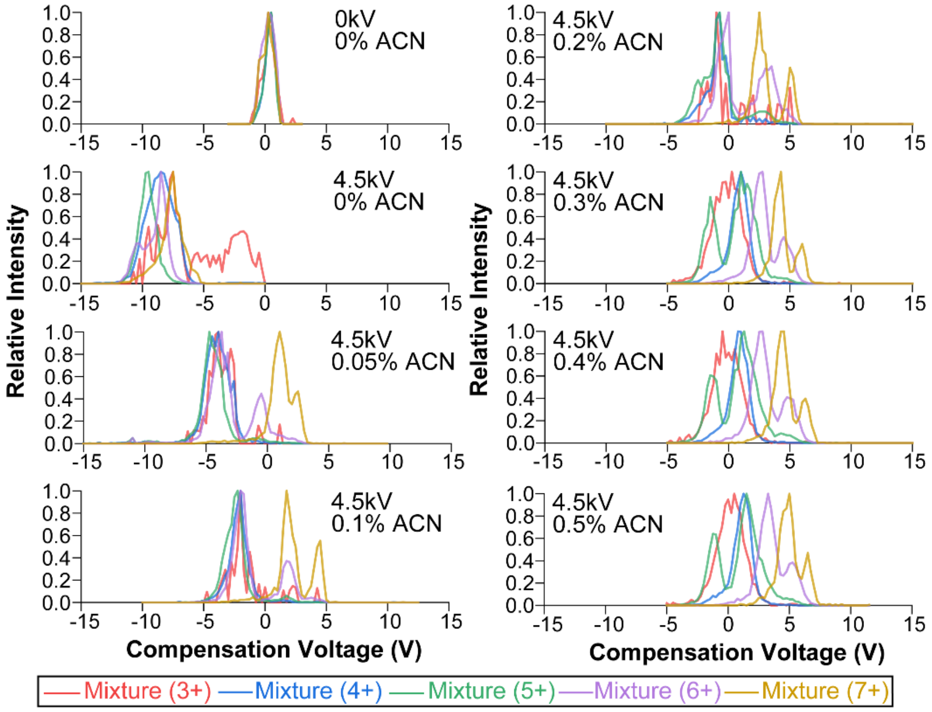

Supplementary Figure 35. Normalized DMS ionograms of the protonated ions of cR6G-**P1**-QSY7 and cR6G-**P2**-QSY7 mixture ($[M+nH]^{n+}$, n=3 to 7) in 0 to 0.5 mol % acetonitrile (ACN). DV was set to 4.5 kV for all experiments.

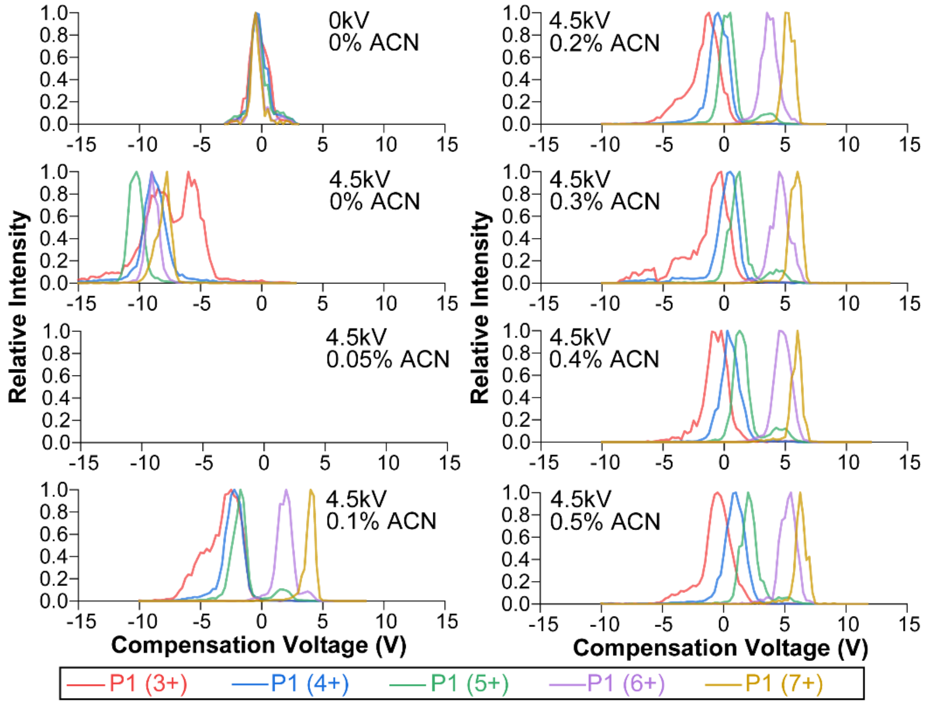

Supplementary Figure 36. Normalized DMS ionograms of the protonated ions of cR6G-**P1**-QSY7 ($[M+nH]^{n+}$, n=3 to 7) in 0 to 0.5 mol % ACN. DV was set to 4.5 kV for all experiments.

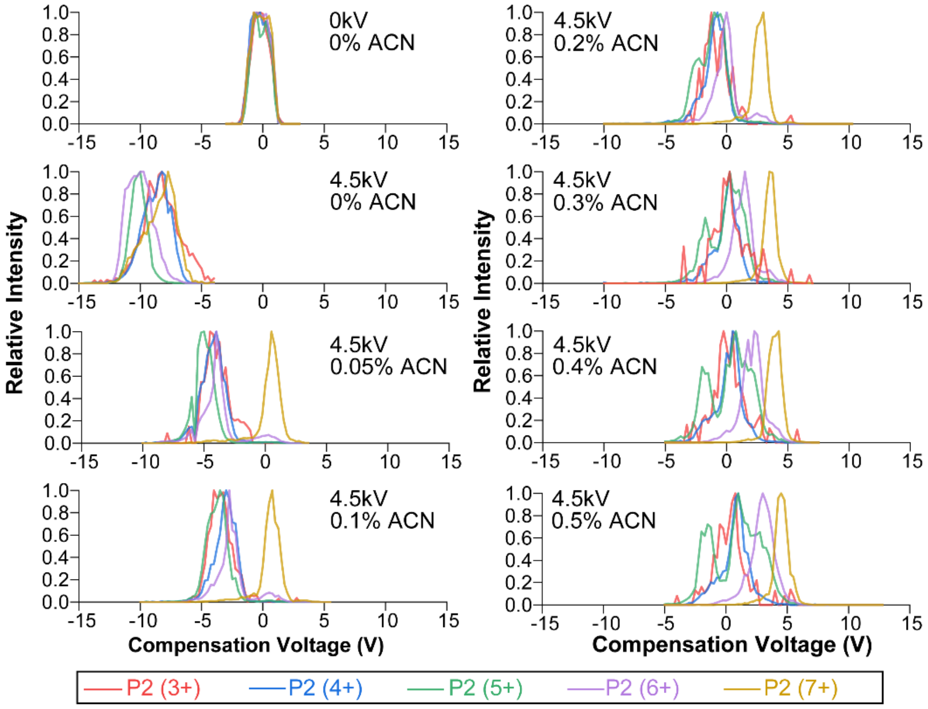

Supplementary Figure 37. Normalized DMS ionograms of the protonated ions of cR6G-**P2**-QSY7 ($[M+nH]^{n+}$, n=3 to 7) in 0 to 0.5 mol % ACN. DV was set to 4.5 kV for all experiments.

Supplementary Note 6. Microsolvation studies

Supplementary Table 19. Calculated parameters of 20 selected structures, with the positively charged cR6G and QSY7 as favorable interaction site.

Peptide	Conf. #	#Frame / Structure	Solvation sites	Solvation free Energy (kcal/mol)
cR6G-**P1**-QSY7	1	415	9	-129
		2114	13	-197
		2959	16	-195
		3207	11	-165
		4181	15	-217
		**Average**	**12.8**	**-181**
	2	114	15	-202
		3911	12	-158
		3934	11	-152
		4107	12	-186
		4519	11	-157
		**Average**	**12.2**	**-171**
cR6G-**P2**-QSY7	1	38	10	-136
		227	10	-142
		1464	9	-137
		3562	8	-119
		4962	10	-139
		**Average**	**9.4**	**-135**
	2	406	11	-165
		407	10	-153
		1995	10	-154
		2874	8	-120
		3646	7	-107
		**Average**	**9.2**	**-140**

Supplementary Note 7. References

1. Tiwari, P., Wu, R., Metternich, J. B. & Zenobi, R. Transition Metal Ion FRET in the Gas Phase: A 10–40 Å Range Molecular Ruler for Mass-Selected Biomolecular Ions. *J. Am. Chem. Soc.* **143**, 11291–11295 (2021).
2. Stiving, A. Q. *et al.* Surface-Induced Dissociation: An Effective Method for Characterization of Protein Quaternary Structure. *Anal. Chem.* **91**, 190–209 (2019).
3. Metternich JB, Katzberger P, Kamenik AS, Tiwari P, Wu R, Riniker S, et al. Influence of the fluorophore mobility on distance measurements by gas phase FRET. ChemRxiv. Cambridge: Cambridge Open Engage; 2023; This content is a preprint and has not been peer-reviewed.
4. Miller, R. A., Nazarov, E. G., Eiceman, G. A. & King, A. T. A MEMS radio-frequency ion mobility spectrometer for chemical vapor detection. *Sensors Actuators, A Phys.* **91**, 301–312 (2001).
5. Levin, D. S., Vouros, P., Miller, R. A., Nazarov, E. G. & Morris, J. C. Characterization of Gas-Phase Molecular Interactions on Differential Mobility Ion Behavior Utilizing an Electrospray Ionization-Differential Mobility-Mass Spectrometer System. *Anal. Chem.* **78**, 96–106 (2006).
6. Bylda, C., Thiele, R., Kobold, U., Bujotzek, A. & Volmer, D. A. Rapid quantification of digitoxin and its metabolites using differential ion mobility spectrometry-tandem mass spectrometry. *Anal. Chem.* **87**, 2121–2128 (2015).
7. Ruskic, D., Klont, F. & Hopfgartner, G. Clustering and Nonclustering Modifier Mixtures in Differential Mobility Spectrometry for Multidimensional Liquid Chromatography Ion Mobility–Mass Spectrometry Analysis. *Anal. Chem.* **93**, 6638–6645 (2021).
8. Tiwari, P., Czar, M. F. & Zenobi, R. Fluorescence-Based Detection of the Desolvation Process of Protein Ions Generated in an Aqueous Electrospray Plume. *Anal. Chem.* **93**, 3635–3642 (2021).
