# Supplementary material for: Determining the gas-phase structures of α-helical peptides from shape, microsolvation, and intramolecular distance data
Source: Nat Commun. 2023 May 22;14:2913. doi: 10.1038/s41467-023-38463-z (PMC10203302; doi:10.1038/s41467-023-38463-z)
Supplement: Supplementary file 2 — Reporting Summary [file 41467_2023_38463_MOESM2_ESM.pdf]

nature portfolio

Corresponding author(s): Renato Zenobi; Sereina Riniker

Last updated by author(s): 17.2.2023

Reporting Summary

Nature Portfolio wishes to improve the reproducibility of the work that we publish. This form provides structure for consistency and transparency in reporting. For further information on Nature Portfolio policies, see our Editorial Policies and the Editorial Policy Checklist.

Statistics

For all statistical analyses, confirm that the following items are present in the figure legend, table legend, main text, or Methods section.

n/a	Confirmed	
☐	☒	The exact sample size (*n*) for each experimental group/condition, given as a discrete number and unit of measurement
☒	☐	A statement on whether measurements were taken from distinct samples or whether the same sample was measured repeatedly
☒	☐	The statistical test(s) used AND whether they are one- or two-sided<br>*Only common tests should be described solely by name; describe more complex techniques in the Methods section.*
☒	☐	A description of all covariates tested
☒	☐	A description of any assumptions or corrections, such as tests of normality and adjustment for multiple comparisons
☐	☒	A full description of the statistical parameters including central tendency (e.g. means) or other basic estimates (e.g. regression coefficient) AND variation (e.g. standard deviation) or associated estimates of uncertainty (e.g. confidence intervals)
☒	☐	For null hypothesis testing, the test statistic (e.g. *F*, *t*, *r*) with confidence intervals, effect sizes, degrees of freedom and *P* value noted<br>*Give P values as exact values whenever suitable.*
☒	☐	For Bayesian analysis, information on the choice of priors and Markov chain Monte Carlo settings
☒	☐	For hierarchical and complex designs, identification of the appropriate level for tests and full reporting of outcomes
☒	☐	Estimates of effect sizes (e.g. Cohen's *d*, Pearson's *r*), indicating how they were calculated

Our web collection on statistics for biologists contains articles on many of the points above.

Software and code

Policy information about availability of computer code

Data collection	All data was collected using instrument-specific, commercial data collection software, as already specified in the manuscript: for mass spectrometry: LCQ Tune Plus (version: 2.0, Thermo Fisher) and MassLynx (version: 4.1, Waters); for fluorescence spectroscopy: Solis software (version: 4.31.30024.0, Andor) and Time Harp 260 (version: 3.0.0.0, PicoQuant).
Data analysis	Standard, commercial software was used throughout, as already specified in the manuscript. For general data analysis/ploting: OriginPro 2021, adobe Illustrator 2022, MATLAB (version R2021a, Mathworks), and MSConvert (Version: 3.0.21193-ccb3e0136, ProteoWizard); for CCS evaluations: Collidoscope and IMPACT (version: 0.9.1, University of Oxford); for 3D model creation: NX 11.0 software (Siemens Software); for force field: AmberTools21; for calculations and calculating interaction energies: SHAKE algorithm, GROMACS 2020.5 simulation engine, ParmEd, LINCS algorithm, grid inhomogeneous solvation theory (GIST, GPU-accelerated version of cpptraj); for fluorescence decays: DecayFit software (version 1.4, FluorTools).

For manuscripts utilizing custom algorithms or software that are central to the research but not yet described in published literature, software must be made available to editors and reviewers. We strongly encourage code deposition in a community repository (e.g. GitHub). See the Nature Portfolio guidelines for submitting code & software for further information.

March 2021

Data

Policy information about availability of data

All manuscripts must include a data availability statement. This statement should provide the following information, where applicable:

- Accession codes, unique identifiers, or web links for publicly available datasets
- A description of any restrictions on data availability
- For clinical datasets or third party data, please ensure that the statement adheres to our policy

Source data are provided with this paper. PDB structures shown in the manuscript are attached to the supplementary Tables 15–18. The original data used in this publication are made available in a curated data archive at ETH Zurich (https://www.researchcollection.ethz.ch) under the DOI: 10.3929/ethz-b-000545943.

Human research participants

Policy information about studies involving human research participants and Sex and Gender in Research.

Reporting on sex and gender	n/a
Population characteristics	n/a
Recruitment	n/a
Ethics oversight	n/a

Note that full information on the approval of the study protocol must also be provided in the manuscript.

Field-specific reporting

Please select the one below that is the best fit for your research. If you are not sure, read the appropriate sections before making your selection.

☒ Life sciences ☐ Behavioural & social sciences ☐ Ecological, evolutionary & environmental sciences

For a reference copy of the document with all sections, see nature.com/documents/nr-reporting-summary-flat.pdf

Life sciences study design

All studies must disclose on these points even when the disclosure is negative.

Sample size	n/a
Data exclusions	n/a
Replication	n/a
Randomization	n/a
Blinding	n/a

Reporting for specific materials, systems and methods

We require information from authors about some types of materials, experimental systems and methods used in many studies. Here, indicate whether each material, system or method listed is relevant to your study. If you are not sure if a list item applies to your research, read the appropriate section before selecting a response.

Materials & experimental systems

n/a	Involved in the study
☒	☐ Antibodies
☒	☐ Eukaryotic cell lines
☒	☐ Palaeontology and archaeology
☒	☐ Animals and other organisms
☒	☐ Clinical data
☒	☐ Dual use research of concern

Methods

n/a	Involved in the study
☒	☐ ChIP-seq
☒	☐ Flow cytometry
☒	☐ MRI-based neuroimaging
